# Self-Assembly of Disorazole C_1_ through a One-Pot Alkyne Metathesis Homodimerization Strategy**

**DOI:** 10.1002/anie.201501922

**Published:** 2015-04-29

**Authors:** Kevin J Ralston, H Clinton Ramstadius, Richard C Brewster, Helen S Niblock, Alison N Hulme

**Affiliations:** EaStCHEM School of Chemistry, University of Edinburgh, David Brewster RoadEdinburgh, EH9 3FJ (UK)

**Keywords:** alkyne metathesis, cytotoxicity, natural products, self-assembly, total synthesis

## Abstract

Alkyne metathesis is increasingly explored as a reliable method to close macrocyclic rings, but there are no prior examples of an alkyne-metathesis-based homodimerization approach to natural products. In this approach to the cytotoxic *C_2_*-symmetric marine-derived bis(lactone) disorazole C_1_, a highly convergent, modular strategy is employed featuring cyclization through an ambitious one-pot alkyne cross-metathesis/ring-closing metathesis self-assembly process.

The use of ring-closing alkyne metathesis (RCAM) in the synthesis of natural products[1] has gained prevalence with the development of reliable, bench-stable catalysts, which have improved substrate scope over their predecessors.[2] These catalysts open up options for the application of RCAM-based strategies to the cyclization of terminal methyl-substituted alkynes,[1] terminal alkynes,[3] and even combinations of the two substitution patterns[4] in the synthesis of natural products. Partial hydrogenation of the resultant macrocyclic alkyne can now be effected under a range of conditions and allows access to either *E*- or *Z*-alkene geometry.[1a,^5^] Yet despite these advances, and in sharp contrast to their alkene counterparts,[6] the development of alkyne cross-metathesis (ACM) reactions remains comparatively underdeveloped.[7] Combining ACM and RCAM reactions to allow self-assembly processes is limited to a handful of examples, including the formation of aryleneethynylene macrocycles and a tetrameric cage structure with 4*D*_2*h*_ symmetry.[7a,^8^] There are no prior examples of such a self-assembly approach to the synthesis of complex natural products.

In order to investigate the application of a combined ACM and RCAM strategy to the synthesis of natural products, our chosen target was the cytotoxic, *C*_2_-symmetric bis(lactone) disorazole C_1_ (**1**; Figure 1),[9] which was first isolated in 1994 from the fermentation broth of the myxobacterium *Sorangium cellulosum*.[10] As a family, the disorazoles have been shown to possess cytotoxicity in the nm to pm range and anti-tubulin activity,[11] and current pharmaceutical interest focuses on their potential for the treatment of drug-resistant solid tumors.[12,13] However, much remains to be discovered about the mode of action of these natural products, including how and where they bind to tubulin,[14] as this has been demonstrated to be orthogonal to the binding sites of vincristine and taxol.[14] Until recently, only one total synthesis of this challenging target was reported.[15,16]

**Figure 1:**
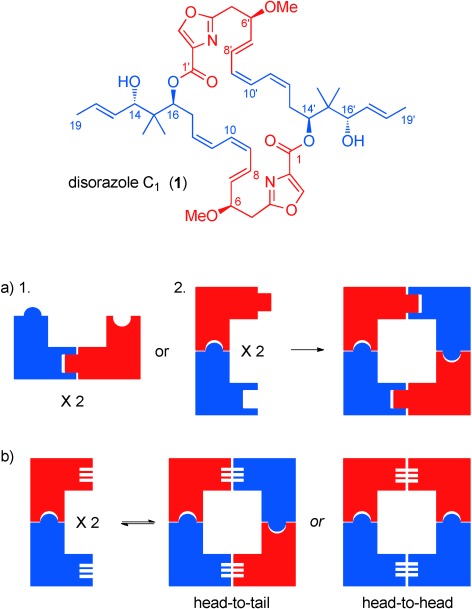
Structure of disorazole C_1_ and overview of synthetic approaches to disorazole C_1_. a) Previous approaches to dimerization using 1. lactonization, or 2. cross-coupling reactions. b) This work, self-assembly using alkyne metathesis.

Pioneering synthetic work was undertaken by Meyers and co-workers before the relative and absolute stereochemistry of disorazole C_1_ had been fully established.[17] Although the total synthesis of disorazole C_1_ was not achieved, this work did identify several pivotal issues to be addressed in any future synthesis; most notably that strategies based on the homodimerization of a fully formed seco-acid precursor (strategy 1 in Figure 1 a) were not likely to be successful because of the competing formation of the monomeric 15-membered lactone, and that the correct choice of protecting group(s) would be crucial to the successful completion of the synthesis.[17b These results are mirrored by the recent work from Hoveyda and co-workers on a seco-acid precursor, which has the correct absolute stereochemistry as well as the *E*,*Z*,*Z*-alkene geometry.[16] Hoffmann et al. also explored a direct dimerization approach, but in this instance with the C9=C10 *Z*-alkene masked as an alkyne.[18] Although an in silico analysis predicted the preferential formation of the desired bis(lactone) over the monomer,[19] neither was formed under a range of conditions. A stepwise coupling to generate the bis(lactone) was achieved by both the Meyers and Hoffmann groups (using C11=C12 and C9=C10 alkyne-masked precursors, respectively) through sequential esterification, unmasking of the second acid and alcohol components, and subsequent lactonization.[17a,^18^]

Avoiding direct dimerization enabled the first successful total synthesis of disorazole C_1_ by Wipf and co-workers in 2004.[15] In their approach, the sequential coupling of components to a C9=C10 alkyne-masked seco-acid through esterification, Sonogashira coupling at C8′=C9′, and subsequent lactonization gave the tetradehydro precursor **2**, which was converted to the natural product in two further steps through PMB deprotection and Lindlar hydrogenation (Scheme 1).[15] Remarkably, the very recent synthesis of disorazole C_1_ by Hoveyda et al. shows that by switching the coupling positions to C10=C11/C10′=C11′ and relying on cross-coupling reactions rather than esterification and lactonization reactions, dimerization is possible even on an alkene, rather than an alkyne-masked, precursor (strategy 2 in Figure 1 a).[16]

**Scheme 1:**
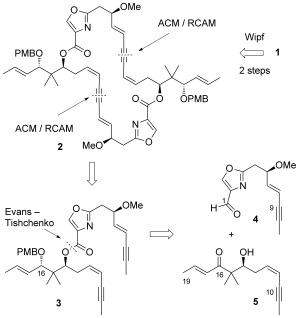
Retrosynthetic analysis of disorazole C_1_ (1).

Our approach to the construction of the 30-membered bis(lactone) differs markedly from the previously reported approaches in that it relies on self-assembly of the bis(lactone) (strategy in Figure 1 b). To this end, we targeted the interception of the Wipf tetradehydro intermediate **2** through metathesis of the bis(alkyne) precursor **3** (Scheme 1). This direct metathesis approach is unprecedented in the synthesis of complex polyketide-derived natural products, but has some foundation in the formation of cyclophanes.[20] However, it does provide an additional challenge over other approaches in that both head-to-tail and head-to-head coupling products could be formed from the nonsymmetrical bis(alkyne) precursor.

Following on from prior work in our group,[21] we planned the formation of bis(alkyne) **3** through the coupling of aldehyde **4** and β-hydroxy ketone **5** using a heteroaryl Evans–Tishchenko (ET) reaction to set the stereochemistry at C16 (Scheme 1).[22,23] Our initial route to C1–C9 oxazole aldehyde **4** relied upon a lateral lithiation of protected 4-hydroxymethyl-2-methyl-oxazole **6**[24] and coupling to enyne aldehyde **7**[25] to generate **8** (Scheme 2). Methylation of the resulting free hydroxy group at C6 gave **9**, and deprotection of the hydroxy group at C1, followed by oxidation generated racemic aldehyde (±)-**4** (Scheme 2). This approach was successful when the protecting group allowed coordination (e.g. PG=MOM), but not for silyl protecting groups, thus suggesting an initial lithiation of the 5-position of the oxazole, with subsequent equilibration to the 2-(lithiomethyl)oxazole species facilitated by diethylamine.[26] Although a few options were explored for converting this route to an asymmetric one,[27] several steps were low-yielding, and we thus pursued an alternative route to a single enantiomer of the desired oxazole aldehyde **4**, which had the additional advantage that it would allow a rapid variation of the heterocycle in future studies.

**Scheme 2:**
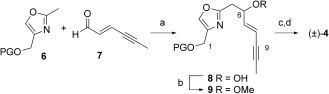
Synthesis of the racemic C1–C9 fragment. Reagents and conditions (PG=MOM): a) 1. *n*BuLi, THF, −78 °C, 1 h; 2. HNEt_2_, −78 °C, 45 min; 3. 7, −78 °C, 45 min, 40 %; b) 1. NaH, THF, 0 °C; 2. MeI, 0 °C to RT, 18 h, 29 %; c) HCl, MeOH, RT, 18 h, 74 %; d) DMP, NaHCO_3_, CH_2_Cl_2_, 0 °C, 2 h, 43 %. PG=protecting group.

Commercially available mannitol derivative **10** (Scheme 3) was converted into alkyne **11** by periodate cleavage, Seyferth–Gilbert homologation with the Ohira–Bestmann reagent,[28] and acetal hydrolysis. Vinyl iodide **12** was accessed by a highly (*E*)-selective palladium-catalyzed hydrostannylation (**13**, *E*/*Z*=30:1) and subsequent iodonolysis.[29] Negishi coupling and selective monotosylation in the presence of catalytic dibutyltin oxide[30] furnished secondary alcohol **14**, which then smoothly underwent methylation to give key tosylate **15**. Attempts to couple this tosylate directly with the 2-position of a 4-substituted oxazole using either C=H activation or lithiation strategies were unsuccessful.[27] Instead, a step-wise construction of the oxazole was achieved through conversion of **15** into the corresponding acid **16** by cyanide displacement and hydrolysis. Subsequent coupling to serine methyl ester, cyclization, and oxidation provided the fully functionalized oxazole **17**, which model studies suggest might be readily converted to the desired oxazole aldehyde **4** through DIBAL-H reduction.

**Scheme 3:**
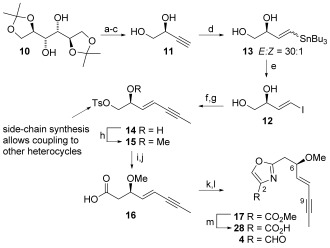
Synthesis of the C1–C9 oxazole fragment. Reagents and conditions: a) NaIO_4_, NaHCO_3_ (satd. aq.), MgSO_4_, CH_2_Cl_2_, 0 °C to RT, 2 h 20 min, 88 %; b) dimethyl-1-diazo-2-oxopropylphosphonate, K_2_CO_3_ (s), MeOH, 0 °C to RT, 17 h; c) HCl (conc.), MeOH, Et_2_O, THF, 6 h 15 min, 66 % over 2 steps; d) [PdCl_2_(PPh_3_)_2_], Bu_3_SnH, Et_2_O, −30 °C, 30 min, 60 %; e) I_2_, Et_2_O, 0 °C, 5 min, 92 %; f) 1. ZnCl_2_, BrMgC=CCH_3_, THF, 0 °C, 15 min; 2. 12, [PdCl_2_(PPh_3_)_2_], 0 °C to RT, 25 h; g) Bu_2_SnO, TsCl, NEt_3_, CH_2_Cl_2_, 0 °C to RT, 11 h, 60 % over 2 steps; h) Me_3_OBF_4_, 1,8-bis(dimethylamino)naphthalene; i) KCN, Bu_4_NI, NaHCO_3_, DMSO, 60 °C to 70 °C, 4 h, 63 % over 2 steps; j) H_2_O_2_, LiOH⋅H_2_O, EtOH, RT, 34 h, 75 %; k) HBTU, EtN(*i*Pr)_2_, SerOMe⋅HCl, CH_3_CN, 0 °C to RT, 10 h, 82 %; l) 1. XtalFluor-E, K_2_CO_3_, CH_2_Cl_2_, −78 °C to 0 °C, 50 min; 2. BrCCl_3_, DBU, −20 °C to RT, 4 h 45 min, 76 %; m) LiOH (aq.), THF, RT, 8 h, 96 %.

A number of routes were pursued to the second key fragment, the C10–C19 β-hydroxy ketone **5**,[31] but its preparation on a gram scale was eventually achieved as shown in Scheme 4. Thus, known aldehyde **18** was subjected to an enantioselective organoborane-mediated Mukaiyama aldol reaction[32] with the silyl ketene acetal of methyl 2-methylpropionate to afford β-hydroxyketone **19** in 85 % yield and 89 % *ee*.[19] Silyl protection of the secondary alcohol **20**, deprotection (DDQ) of the PMB-ether, Swern oxidation of the primary alcohol and a Stork–Zhao–Wittig reaction[33] gave the key vinyl iodide **21** (*Z*/*E*>99:1). A Negishi coupling was used to install the enyne portion of the target fragment **5** in high yield. Subsequent conversion of the ester **22** to the Weinreb amide **24** was best performed on the free alcohol **23**, which was reprotected (**25**) for the following allyl Grignard addition and DBU-mediated isomerization to the enone. Deprotection (with HF) gave the required β-hydroxyketone **5** and completed the fragment in 11 steps and 28 % overall yield from **18**.

**Scheme 4:**
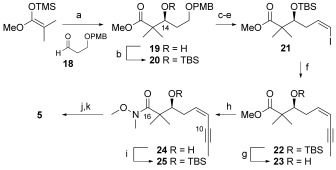
Synthesis of the C10–C19 β-hydroxy ketone fragment 3. Reagents and conditions: a) 1. *N*-Ts-D-Valine, BH_3_⋅THF, CH_2_Cl_2_, −78 °C, 5 h, 2. HCl, THF:H_2_O (1:1), 85 %, 89 % *ee*; b) TBSOTf, 2,6-lutidine, CH_2_Cl_2_, −78 °C, 2.5 h, 94 %; c) DDQ, CH_2_Cl_2_:H_2_O (18:1), RT, 1.5 h, quant.; d) Swern, 95 %; e) ICH_2_PPh_3_^+^I^−^, NaHMDS, HMPA, THF, −78 °C, 2 h, 75 %; f) 1. BrMgC≡CCH_3_, ZnCl_2_, THF, 0 °C, 30 min, 2. 20, [PdCl_2_(PPh_3_)_2_], 0 °C to RT, 16 h, 92 %; g) HF (40 % aq.), MeCN, 0 °C to RT, 1 h, 99 %; h) 1. HNMe(OMe)⋅HCl, *n*BuLi, −78 °C to RT, 30 min, 2. 22, THF:hexane (1:1), −78 °C to RT, 1.5 h, 82 %; i) TBSOTf, 2,6-lutidine, CH_2_Cl_2_, −78 °C to RT, 2 h, 96 %; j) 1. allyl-MgBr, Et_2_O −20 to −78 °C, 2 h, 2. DBU, Et_3_N, 50 °C, 18 h, 81 %; k) HF (40 % aq.), MeCN, 0 °C to RT, 45 min, 85 %.

The preparation of β-hydroxyketone **5** on a gram scale allowed the examination of the scope of the ET reaction with model aryl and heteroaryl aldehydes. Electron-deficient aldehydes (in particular pyridines and nitrobenzaldehydes) were shown to undergo a Sm^III^-catalyzed ET reaction, giving 1,3-*anti* diol monoester products in good to excellent yields (>95:5 d.r.). Unfortunately, indoles, pyrroles, and pyrazoles,[23] even those containing electron-withdrawing substituents at the heteroatom, gave poor yields or failed to react. Thus, the critical *anti* stereorelationship at C14–C16 was set using an ET coupling with 3-nitrobenzaldehyde to give **26** (Scheme 5); a PMB protecting group was installed, and the hydroxy group at C14 was unmasked through subsequent ester hydrolysis to give **27**. Yamaguchi coupling, through the portionwise addition of the activated acid derived from the C1–C9 fragment **28** (Scheme 3) to fragment **27** gave the PMB-protected bis(alkyne) **3** spanning the C1–C9/C10′–C19′ portion of disorazole C_1_. This bis(alkyne) could be readily deprotected under the controlled conditions used in the total synthesis reported by Wipf[15] to give the bis(alkyne) alcohol **29**.

**Scheme 5:**
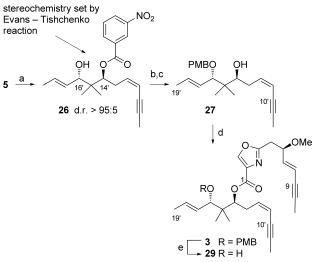
Coupling of the C1–C9 and C10′–C19′ fragments to give bis(alkyne) intermediate 3. Reagents and conditions: a) 3-NO_2_PhCHO, SmI_2_, THF, −20 °C, 4 h, 94 %; b) PMB-TCA, Sc(OTf)_3_, PhCH_3_, 0 °C to RT, 1 h, 79 %; c) LiOH, MeOH:H_2_O (10:1), reflux, 18 h, 91 %; d) 1. 2,4,6-trichlorobenzoyl chloride, Et_3_N, 28, PhCH_3_, RT, 30 min, added portionwise to 27 (0.05 m in toluene), DMAP, 40 °C, 30 min each addition, 2. 40 °C, 18 h 71 %; e) DDQ, CH_2_Cl_2_:phosphate buffer (1:1), RT, 30 min, 77 %.

With bis(alkynes) **3** and **29** in hand, we could now explore the ambitious ACM/RCAM self-assembly process (Scheme 6). We were encouraged by the computational studies of Hoffmann et al., which suggested that dimer formation was favored for a C9–C10 based bis(alkyne),[19] and by our own preliminary modelling studies, which indicated a thermodynamic preference for the desired head-to-tail coupling mode for the parent disorazole C_1_ structure. For the metathesis reaction, we chose the recently published molybdenum alkylidyne catalyst **30** from the Fürstner group.[2a The reaction was initiated in a glovebox and we found that the overall yields and conversion were considerably improved if the substrate was stirred with a mixture of 4 Å and 5 Å activated molecular sieves prior to the addition of the catalyst.[4] The outcome seemed to depend on the initial amount of catalyst added and could not be improved by the later addition of further portions of catalyst. The reaction was best followed by reverse-phase LC-MS (*λ*=254 nm), which allowed distinct peaks corresponding to each of the linear (**31**, **32**, **33**) and cyclic (**2**, **34**) dimers to be identified. Under optimum conditions (Scheme 6), cyclic dimers **2** and **34** could be isolated in an overall yield of 62 %; the ratio of the desired head-to-tail-coupled product **2** to its head-to-head-coupled regioisomer **34** was approximately 5:1. The formation of the unwanted cyclic monomer was not discernible by LC-MS, thus supporting computational predictions.[34] Data for tetradehydrido disorazole C_1_ (**2**) was found to be identical in all regards to that reported by the Wipf group in 2004, and this intermediate could be converted in two steps via the known alcohol **35** to the target natural product, disorazole C_1_ (**1**), using the previously reported procedures.[15,35] When we attempted the self-assembly reaction with the deprotected bis(alkyne) **29**, a complex mixture of products was produced. While LC-MS indicated a possible correlation with the predicted linear (**36**, **37**, **38**) and cyclic (**35**, **39**) dimers, isolation of the major peak with the desired *m*/*z*[36] gave material which had distinctly different NMR data to that which we had already determined for compound **35**, thus indicating a more complex process (perhaps with accompanying double bond isomerism) for the deprotected material.

**Scheme 6:**
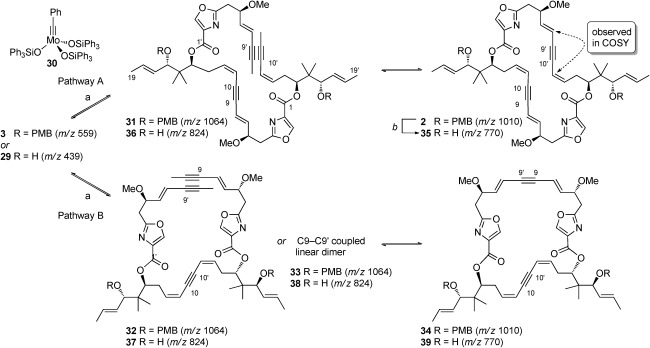
ACM/RCAM self-assembly reaction products. Pathway A: desired head-to-tail bis(alkyne) coupling proceeding via a C9=C10 [or C9′=C10′] coupled linear dimer intermediate. Pathway B: undesired head-to-head coupling proceeding via a C10=C10′ (shown) or C9=C9′ coupled linear dimer intermediate. Reagents and conditions: a) 1. 4 Å/5 Å MS (1:1), PhCH_3_, RT, 20 min; 2. 30 (20 mol %), RT, 16 h; b) DDQ, CH_2_Cl_2_:phosphate buffer (1:1), RT, 30 min, 61 %.

In conclusion, we have demonstrated the first example of an alkyne metathesis self-assembly process, using cross-metathesis and ring-closing metathesis reactions, to give the *C*_2_-symmetric bis(lactone) disorazole C_1_. The synthetic results give an intriguing glimpse into the possibility of using such an approach in the synthesis of complex natural product architectures and open up new routes to the synthesis of analogues of this fascinating natural product.
