# Supplementary material for: Self-Assembly of Disorazole C1 through a One-Pot Alkyne Metathesis Homodimerization Strategy
Source: Angew Chem Int Ed Engl. 2015 Apr 29;54(24):7086–90. doi: 10.1002/anie.201501922 (PMC4517162; doi:10.1002/anie.201501922)
Supplement: Supplementary file 1 [file anie0054-7086-sd1.pdf]

Angewandte Chemie
Eine Zeitschrift der Gesellschaft Deutscher Chemiker

Supporting Information

German Edition: DOI:

Self-Assembly of Disorazole C_1 through a One-Pot Alkyne Metathesis Homodimerization Strategy**

*Kevin J. Ralston, H. Clinton Ramstadius, Richard C. Brewster, Helen S. Niblock, and Alison N. Hulme**

anie_201501922_sm_miscellaneous_information.pdf

Page **Contents**

Page	Contents
S1	General Methods
S2-S4	Synthesis of racemic oxazole-containing aldehyde **4**
S5-S10	Synthesis of oxazole-containing acid **28**
S10-S16	Synthesis of β-hydroxy ketone fragment **5**
S17-S20	Evans-Tishchenko and fragment coupling to give bis-alkynes **3** and **29**
S21-S22	Self-assembly metathesis reaction to give di-lactones **2**, **34**; PMB deprotection to give **35** and Lindlar hydrogenation to give **1**
S23	References

1.1 General Methods

All non-aqueous reactions were carried out under an atmosphere of nitrogen or argon using oven-dried glassware that was cooled in a desiccator prior to use. Unless otherwise noted, starting materials and reagents were obtained from commercial suppliers and were used without further purification. Toluene, THF, CH_2Cl_2, and Et_2O were dried and purified by passage through activated alumina columns using a Glass Contour Solvent Purification System. *N*,*N*-Diethylaniline was distilled from calcium hydride and stored over molecular sieves under a nitrogen atmosphere. Saturated aqueous solutions of inorganic salts are represented as (volume, sat aq). ^{1}H and ^{13}C NMR spectra were obtained on Bruker instruments at the stated frequency. Infra-red spectra were recorded neat on Shimadzu IRAffinity-1 unless otherwise stated. Electrospray (ESI) and electron ionisation (EI) mass spectra were obtained on a Bruker 12 T SolariX, Bruker microTOF II or Kratos MS50TC mass spectrometer. Melting points were determined on a Gallenkamp Electrothermal Melting Point apparatus and are uncorrected. Flash chromatography was carried out using Merck Kieselgel 60 (Merck 9385) under positive pressure. Eluent compositions are quoted as v/v ratios. Optical rotations were performed on an Optical Activity POLAAR 20 polarimeter. Chiral HPLC analysis was performed on an Agilent 1100 instrument using a CHIRALPAK® IC, 5 μm amylose tris (3,5-dichlorophenylcarbamate) coated silica, 4.6 x 250 mm column. R_t values were recorded by analytical reverse phase HPLC analysis using a Waters 600E (100 μL) gradient pump using a 717plus autosampler and a Waters 996 PDA equipped with a Phenomenex Luna C18(2), 5 μm, 250 x 4.6 mm column at a flow rate of 1 mL min^{-1}. Semi-preparative reverse phase HPLC was performed using a Waters 600 (225 μL) system using a Waters 486 tuneable absorbance detector recording at 254 nm equipped with a Phenomonex Luna C18(2), 5 μm, 250 x 21.2 mm column at a flow rate of 21.2 mL min^{-1}.

Synthesis of racemic oxazole-containing aldehyde 4

4-(Methoxymethoxy)methyl-2-methyl-1,3-oxazole 6 (P = MOM)

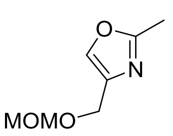

Sodium hydride (0.53 g, 60 % in mineral oil, 13.4 mmol) was added to a solution of 4-hydroxymethyl-2-methyloxazole[1] (1.37 g, 12.2 mmol) in THF (25 mL) at 0 °C, and the mixture was stirred at 0 °C for 10 min and at rt for an additional 1 h. The mixture was cooled to 0 °C and chloromethyl methyl ether (1.10 mL, 14.6 mmol) was added dropwise. The reaction mixture was stirred at 0 °C for 10 min and at rt for an additional 3 h. The mixture was poured into water (50 mL) and extracted with EtOAc (2 × 100 mL). The combined organic extracts were washed with brine (50 mL), dried, and concentrated *in vacuo*. The crude material was purified by flash chromatography (hexane:EtOAc, 3:2) to give the ether **6** as an orange oil (1.41 g, 73 %). **R$_f$** (Hexane:EtOAc, 1:1) = 0.21; **IR** (neat, cm^{-1}) 1581 (C=C); **^{1}H NMR** δ (500 MHz, $CDCl_3$) 7.52 (1H, s, Ar*H*), 4.72 (2H, s, OC*H*$_2$O), 4.48 (2H, s, OC*H*$_2$), 3.41 (3H, s, OC*H*$_3$), 2.46 (3H, s, ArC*H*$_3$); **^{13}C NMR** δ (126 MHz, $CDCl_3$) 162.1 (C), 137.2 (C), 136.0 (CH), 95.8 (CH_2), 60.83 (CH_2), 55.46 (CH_3), 13.99 (CH_3); ***m/z*** (EI) 157 ([M]$^+$, 3 %), 127 (8), 97 (31), 45 (100); **HRMS** (EI) [M]$^+$ found 157.0735, $C_7H_{11}NO_3$ requires 157.0739.

4-(Methoxymethoxy)methyl-2-[(3'*E*)-2-hydroxy-hept-3'-en-5'-yn-1'-yl]-1,3-oxazole 8

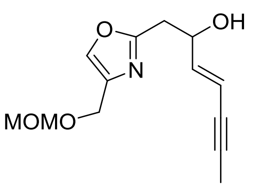

MOM protected oxazole **6** (480 mg, 3.06 mmol) was dissolved in dry THF (10 mL) and was cooled with stirring to −78 °C. To this solution nBuLi (3.83 mL, 1.6 M in hexanes, 6.12 mmol) was added dropwise and the solution allowed to stir for 1 h, after which diethylamine (1.90 mL, 18.36 mmol) was added. After a further 45 min aldehyde **7**[2] (431 mg, 4.58 mmol) was added and the solution was stirred for 45 min at −78 °C. The reaction mixture was quenched with NH_4Cl (10 mL, sat aq) and allowed to warm to rt, the reaction mixture was then extracted with DCM (3 × 20 mL). The organic extracts were combined, dried and concentrated *in vacuo* to give the alcohol. The product was purified by flash chromatography (1:20 MeOH:DCM) twice to give the alcohol **8** as a brown gum (192 mg, 25 %; 40 % yield based on recovered starting material from 2nd column). **R$_f$** (DCM:MeOH, 11:1) = 0.47; **IR** (neat, cm^{-1}) 3383 (OH), 2200 (C≡C), 1610 (C=N), 1570 (C=C), 1149; **^{1}H NMR** δ (400 MHz, $CDCl_3$) 7.55 (1H, s, Ar*H*), 6.08 (1H, dd, *J* = 15.8, 5.7 Hz, CHOHC*H*=CH), 5.78 (1H, dhex, *J* = 15.8, 2.3 Hz, CH=C*H*C), 4.71 (2H, s, OC*H*$_2$O), 4.66-4.64 (1H, m, C*H*OH), 4.49 (2H, s, ArC*H*$_2$O), 3.45 (1H, br s, OH), 3.41 (3H, s, OCH_3), 2.99 (1H, dd, *J* = 16.0, 4.0 Hz, ArC*H*$_X$CH$_Y$CH), 2.90 (1H, dd, *J* = 16.0, 8.3 Hz, ArCH$_X$C*H*$_Y$CH), 1.95 (3H, d, *J* = 2.3 Hz, CC*H*$_3$); **^{13}C NMR** δ (126 MHz, $CDCl_3$) 162.6 (C), 141.6 (CH), 137.3 (C), 136.3 (CH), 111.6 (CH), 95.8 (CH_2), 87.5 (C),* 69.2 (CH), 60.7 (CH_2), 55.5 (CH_3), 35.4 (CH_2), 4.4 (CH_3); ***m/z*** (EI) 251 ([M]$^+$, 22 %), 157 (66), 125 (57), 95 (100); **HRMS** (EI) [M]$^+$ found 251.1152, $C_{13}H_{17}NO_4$ requires 251.1158.
* one alkyne C obscured by $CDCl_3$

4-(Methoxymethoxy)methyl-2-[(3'*E*)-2'-methoxyhept-3'-en-5'-yn-1'-yl]-1,3-oxazole 9

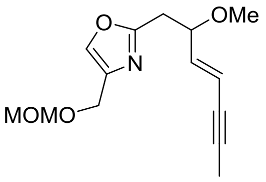

To a solution of alcohol **8** (100 mg, 0.40 mmol) in THF (3 mL) at 0 °C was added NaH (39 mg, 0.99 mmol, 60 % dispersion in mineral oil), and the suspension was stirred for 30 min. Iodomethane (0.05 mL, 0.8 mmol) was then added and the mixture stirred at 0 °C for 40 min and then at rt for ~18 h. The excess NaH was quenched by dropwise addition of $NaHCO_3$ (5 mL, sat aq) and diethyl ether was added (10 mL). The two layers were separated and the organic layer was washed with $Na_2S_2O_3$ (10 mL, 0.1 M), water (10 mL) and brine (10 mL), then

dried and concentrated *in vacuo*. The product was purified by flash chromatography (1:1 EtOAc:hexane) to give the methyl ether **9** as a brown oil (30 mg, 29 %). $\mathbf{R_f}$ (EtOAc:hexane, 1:3) = 0.28; **IR** (neat, cm^{-1}) 2220 (C≡C), 1680 (C=N), 1571 (C=C); ^{1}**H NMR** δ (500 MHz, $CDCl_3$) 7.56 (1H, s, Ar*H*), 5.95 (1H, dd, *J* = 15.9, 7.7 Hz, CHC*H*=CH), 5.70 (1H, dd, *J* = 15.9, 2.0 Hz, CH=C*H*C), 4.74 (2H, s, OCH_2O), 4.53 (2H, s, ArCH_2O), 4.12 (1H, q, *J* = 7.6 Hz, C*H*OMe), 3.43 (3H, s, OCH_3), 3.29 (3H, s, OCH_3), 3.07 (1H, dd, *J* = 15.0, 7.6 Hz, ArCH_XCH_YCH), 2.96 (1H, dd, *J* = 15.0, 5.8 Hz, ArCH_XCH_YCH), 1.97 (3H, d, *J* = 2.0 Hz, CCH_3). ^{13}**C NMR** δ (126 MHz, $CDCl_3$) 162.0 (C), 140.1 (CH), 137.6 (C), 136.2 (CH), 113.7 (CH), 95.9 (CH_2), 87.5 (C), 79.4 (CH),* 61.1 (CH_2), 56.7 (CH_3), 55.5 (CH_3), 34.8 (CH_2), 4.3 (CH_3); ***m/z*** (EI) 265 ($[M]^+$, 4 %), 234 (3), 109 (100), **HRMS** (EI) $[M]^+$ found 265.1318, $C_{14}H_{19}NO_4$ requires 265.1314.
* one alkyne C obscured by $CDCl_3$

4-Hydroxymethyl-2-[(3'*E*)-2'-methoxyhept-3'-en-5'-yn-1'-yl]-1,3-oxazole S1

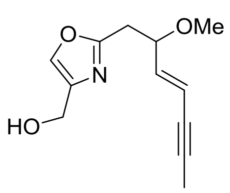

To a solution of MOM protected alcohol **9** in methanol was added HCl (5 drops, conc. aq) and the solution was stirred for ~18 h at rt. The solvent was removed *in vacuo* and the residue was taken into EtOAc, washed with $NaHCO_3$ (10 mL, sat aq). The aqueous phase was extracted with EtOAc (2 × 10 mL). The organic layers were combined, dried and concentrated *in vacuo*, to give the alcohol **S1** as a brown oil 42 mg (74 %). $\mathbf{R_f}$ (MeOH:DCM, 1:9) = 0.50; **IR** (neat, cm^{-1}) 3392 (OH), 2223 (C≡C), 1680 (C=C), 1570 (C=C); ^{1}**H NMR** δ (500 MHz, $CDCl_3$) 7.50 (1H, s, Ar*H*), 5.90 (1H, dd, *J* = 15.9, 7.7 Hz, CHC*H*=CH), 5.67 (1H, dq, *J* = 15.9, 2.2 Hz, CH=C*H*C), 4.55 (2H, s, CH_2OH), 4.06 (1H, q, *J* = 7.4 Hz, C*H*OMe), 3.25 (3H, s, OCH_3), 3.02 (1H, dd, *J* = 15.1, 7.4 Hz, ArCH_XCH_YCH), 2.91 (1H, dd, *J* = 15.1, 5.7 Hz, ArCH_XCH_YCH), 1.93 (3H, d, *J* = 2.2 Hz, CCH_3). ^{13}**C NMR** δ (126 MHz, $CDCl_3$) 162.0 (C), 140.3 (C), 139.9 (CH), 135.1 (CH), 113.9 (CH), 87.6 (C), 79.3 (CH),* 56.7 (CH_2), 56.6 (CH_3), 34.7 (CH_2), 4.3 (CH_3); ***m/z*** (EI) 221 ($[M]^+$, 26 %), 167 (27), 149 (34), 139 (44), 109 (100); **HRMS** (EI) $[M]^+$ found 221.1054, $C_{12}H_{15}NO_3$ requires 221.1052.
* one alkyne C obscured by $CDCl_3$

2-[(3'*E*)-2'-Methoxyhept-3'-en-5'-yn-1'-yl]-1,3-oxazole-4-carbaldehyde (±)-4

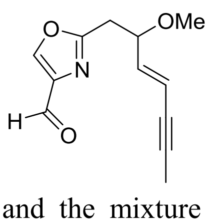

$NaHCO_3$ (201 mg, 2.4 mmol) was suspended in DCM (5 mL) and Dess-Martin periodinane (100 mg, 0.024 mmol) was added and the suspension stirred for 5 min at rt. The mixture was cooled to 0 °C and alcohol **S1** (35 mg, 0.16 mmol) was added in DCM (1 mL) and the suspension was stirred at 0 °C for 2 h, after which a 1:1 mixture of $NaHCO_3$ (5 mL, sat aq) and $Na_2S_2O_3$ (5 mL, 0.1 M aq) was added and the mixture stirred for 10 min. The biphasic mixture was separated and the aqueous layer extracted with DCM (2 × 10 mL), the organic layers were combined, dried and evaporated *in vacuo*. The product was purified by flash chromatography (1:1 EtOAc:hexane) to give the aldehyde **(±)-4** as a brown oil (15 mg, 43 %). $\mathbf{R_f}$ (EtOAc:hexane, 1:1) = 0.38; **IR** (neat, cm^{-1}) 2220 (C≡C), 1699 (C=O), 1583 (C=C); ^{1}**H NMR** δ (500 MHz, $CDCl_3$) 9.91 (1H, s, C*H*O) 8.19 (1H, s, Ar*H*), 5.92 (1H, dd, *J* = 15.9, 7.7 Hz, CHC*H*=CH), 5.70 (1H, dqd, *J* = 15.9, 2.1, 0.9 Hz, CH=C*H*C), 4.15-4.11 (1H, m, C*H*OMe), 3.27 (3H, s, OCH_3), 3.10 (1H, dd, *J* = 15.2, 7.7 Hz, ArCH_XCH_YCH), 3.01 (1H, dd, *J* = 15.2, 5.5 Hz, ArCH_XCH_YCH), 1.95 (3H, d, *J* = 2.1 Hz, CCH_3). ^{13}**C NMR** δ (126 MHz, $CDCl_3$) 184.0 (CH), 163.1 (C), 144.4 (CH), 140.9 (C), 139.4 (CH), 113.3 (CH), 88.0 (C), 79.1 (CH),* 56.7 (CH_3), 34.6 (CH_2), 4.3 (CH_3); ***m/z*** (EI) 219 ($[M]^+$, 23 %), 204 (11), 188 (17), 167 (20), 149 (41), 109 (100), **HRMS** (EI) $[M]^+$ found 219.0898, $C_{12}H_{13}NO_3$ requires 219.0895.
* one alkyne C obscured by $CDCl_3$

Synthesis of oxazole-containing acid 28

D-(*R*)-Glyceraldehyde acetonide S2

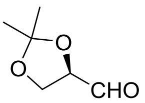

1,2:5,6-di-*O*-isopropylidene-D-mannitol **10** (4.06 g, 15.5 mmol) was dissolved in CH_2Cl_2 (40 mL), $NaHCO_3$ (1.9 mL; sat aq) was added and the resulting biphasic mixture was cooled to 0 °C. After 10 min, $NaIO_4$ (6.67 g, 31.2 mmol) was added in one portion and after a further 2 h and 20 min, TLC (hexane:EtOAc, 1:1) indicated the complete consumption of starting material (R_f 0.1) and the formation of a major product R_f (0.2). Anhydrous $MgSO_4$ (2.55 g) was added and after a further 40 min, the reaction mixture was filtered. The solid filter cake was transferred back to the reaction flask, CH_2Cl_2 (40 mL) was added and the resulting suspension stirred for an additional 30 min, after which it was filtered. The combined organic filtrates were carefully concentrated *in vacuo* and the resulting colourless liquid was purified by distillation under reduced pressure (approximately 80 mbar) and at a temperature of 80-85 °C, to yield aldehyde **S2** (3.56 g, 88%) as a colourless liquid; **$[\alpha]_D$** +40.0 (c 1.0 , $CHCl_3$), lit.[3] $[\alpha]_D$ +53.8 (c 2.0 , $CHCl_3$); **IR** (neat, cm^{-1}) 2891 (CHO), 1734 (C=O); **^{1}H NMR** δ (500 MHz, $CDCl_3$) 9.72 (1H, d, *J* = 1.9 Hz, C*H*O), 4.38 (1H, ddd, *J* = 7.3, 4.7, 1.9 Hz, OCH_2C*H*O), 4.20 (1H, dd, *J* = 8.8, 7.3 Hz, OCH_aH_b (H_a)), 4.10 (1H, dd, *J* = 8.8, 4.7 Hz, OCH_aH_b (H_b)), 1.49 (3H, s, CH_3), 1.42 (3H, s, CH_3); **^{13}C NMR** δ (126 MHz, $CDCl_3$) 202.0 (CO), 111.4 (C), 80.0 (CH), 65.7 (CH_2), 26.4 (CH_3), 25.3 (CH_3). ^{1}H NMR spectroscopic data in good agreement with the literature.[4]

(2*S*)-3-Butyn-1,2-diol 11

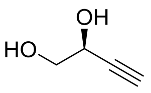

Method 1: Dimethyl (1-diazo-2-oxypropyl) phosphonate (0.78 mL, 5.2 mmol) was dissolved in MeOH (6 mL) and cooled to 0 °C. K_2CO_3 (728 mg, 5.3 mmol) was added and the resulting light yellow suspension was stirred for 10 min at 0 °C before a solution of aldehyde **S2** (450 mg, 3.46 mmol) in MeOH (3 + 1 mL) was added by cannula. After 2 h, the reaction mixture was allowed to warm to rt and after a further 15 h, TLC (hexane:EtOAc, 9:1) indicated the presence of small amounts of starting material ($R_f \approx 0$) and the formation of a major product (R_f 0.5). The reaction mixture was diluted with ether (60 mL) and washed with $NaHCO_3$ (2 x 25 mL; sat aq). The combined aqueous phases were re-extracted with ether (2 x 20 mL). [For characterisation a sample of the volatile (*S*)-3-butyne-1,2-di-*O*-isopropylidene acetal intermediate was purified by column chromatography (hexane:diethyl ether, 25:1→ 20:1 → 15:1→ 10:1) to yield a colourless liquid; **^{1}H NMR** δ (500 MHz, $CDCl_3$) 4.71 (1H, appt td, *J* = 6.3, 2.1 Hz, OCH_2C*H*O), 4.18 (1H, dd, *J* = 8.1, 6.4 Hz, OCH_aH_b (H_a)), 3.95 (1H, dd, *J* = 8.1, 6.2 Hz, OCH_aH_b (H_b)), 2.50 (1H, d, *J* = 2.1 Hz, C≡CH), 1.50 (3H, s, CH_3), 1.39 (3H, s, CH_3); **^{13}C NMR** δ (126 MHz, $CDCl_3$) 110.7 (C), 81.5 (C), 74.1 (CH), 70.0 (CH_2), 65.4 (CH), 26.3 (CH_3), 26.1 (CH_3).] The combined ether fractions were transferred to a round-bottomed flask, to which was added MeOH (5 mL), THF (5 mL) and HCl (1.8 mL; conc). After 6 h 15 min, TLC (hexane:EtOAc, 1:3) indicated the complete consumption of the starting acetal ($R_f \approx 1$) and the formation of a major product (R_f 0.4). Triethylamine (3 mL, 21.6 mmol) was added and the resulting mixture was concentrated *in vacuo*. The crude product was purified by column chromatography (dry load, hexane:EtOAc, 1:1 → 1:2 → 1:3 → 0:1) to yield diol **11** (198 mg, 66%) as an off-white solid; **mp** 37–39 °C (hexane/EtOAc), lit.[5] 34–35 °C; **$[\alpha]_D$** +37.0 (c 1.0, $CHCl_3$), lit.[5] $[\alpha]_D$ +35.5 (c 1.07, $CHCl_3$); **IR** (neat, cm^{-1}) 3437 (br, OH), 3285 (s, C≡C-H); **^{1}H NMR** δ (500 MHz, $CDCl_3$) 4.47 (1H, ddd, *J* = 6.1, 3.8, 2.1 Hz, OCH_2C*H*O), 3.78 (1H, dd, *J* = 11.4, 3.8 Hz, OCH_aH_b (H_a)), 3.70 (1H, dd, *J* = 11.4, 6.1 Hz, OCH_aH_b (H_b)), 2.51 (1H, d, *J* = 2.1 Hz, C≡C*H*), 2.45 (1H, br s, OH), 2.14 (1H, br s, OH); **^{13}C NMR** δ (126 MHz, $CDCl_3$) 81.6 (C), 74.4 (CH), 66.4 (CH_2), 63.1 (CH); ***m/z*** (ESI+) 109 ($[M+Na]^+$, 38%), 87 ($[M+H]^+$, 5); **HRMS** (ESI+) $[M+H]^+$; found 87.0440, $C_4H_7O_2$ requires 87.0441.

Method 2: 1,2,5,6-di-*O*-isopropylidene-D-mannitol **10** (3.64 g, 13.9 mmol) was dissolved in CH_2Cl_2 (35 mL), $NaHCO_3$ (1.75 mL; sat aq) was added and the resulting biphasic mixture was cooled to 0 °C. After 15 min, $NaIO_4$ (5.89 g, 27.5 mmol) was added in one portion. After 20 min, the cooling was removed and the reaction mixture was allowed to warm to rt. After a further 2 h, anhydrous $MgSO_4$ (3.1 g) was added and after a further 20 min, the reaction mixture was filtered. The solid filter cake was transferred back to the reaction flask and CH_2Cl_2 (30 mL) was added and the resulting suspension stirred for an additional 20 min, after which it was filtered. The combined organic filtrates were carefully concentrated *in vacuo* and the resulting pale yellow liquid was used without any further purification.

Dimethyl (1-diazo-2-oxypropyl) phosphonate (4.0 mL, 25.9 mmol) was dissolved in MeOH (15 mL) and cooled to 0 °C. K_2CO_3 (3.93 mg, 28.4 mmol) was added and the resulting light yellow suspension was stirred for 15 min at 0 °C before a solution of the crude aldehyde **S2** from above was dissolved in THF (7 mL) and added by cannula (rinsing with 3 mL THF). The reaction mixture was slowly allowed to warm to rt and after a further 19 h, TLC (hexane:EtOAc, 9:1) indicated the presence of small amounts of starting material ($R_f \approx 0$) and the formation of a major product (R_f 0.5). The reaction mixture was diluted with ether (60 mL) and washed with $NaHCO_3$ (2 x 60 mL; sat aq). The combined aqueous phases were re-extracted with ether (2 x 60 mL). The combined ether fractions were transferred to a round-bottomed flask, to which was also added MeOH (15 mL) and HCl (4.0 mL; conc). After 19 h, TLC (hexane:EtOAc, 1:3) indicated the complete consumption of the starting acetal ($R_f \approx 1$) and the formation of a major product (R_f 0.4). Triethylamine (6.6 mL, 47.5 mmol) was added and the resulting mixture was concentrated *in vacuo*. The crude product was purified by column chromatography (dry load, hexane:EtOAc, 1:2 → 0:1) to yield diol **11** (1.23 g, 51%) as an off-white solid with characterisation data identical to that above.

(2*S*,3*E*)-4-Tributylstannyl-3-buten-1,2-diol 13

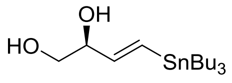

Alkyne **11** (1.23 g, 14.3 mmol) was dissolved in diethyl ether (10 mL) under Ar. After 10 min, the resulting solution was transferred to a flame-dried 3-necked round-bottomed flask by cannula, rinsing with diethyl ether (5 mL). $PdCl_2(PPh_3)_2$ (490 mg, 0.70 mmol) was added and the resulting pale yellow suspension was cooled to -30 °C. Further diethyl ether (6 mL) was added to alleviate thickening of the suspension upon cooling. Bu_3SnH (5.20 mL, 18.7 mmol) was dissolved in diethyl ether (60 mL) in a 100 mL dropping funnel and the resulting solution was added dropwise to the alkyne suspension over a period of 30 min while keeping the temperature at -30 °C. TLC (hexane:EtOAc, 1:1) indicated the complete consumption of starting material (R_f 0.2) and the formation of a minor (R_f 0.8) and a major product (R_f 0.6). The reaction mixture was purified directly (without concentration) by flash column chromatography (hexane:EtOAc, 1:1) to yield approximately 6 g of crude product contaminated with tributyltin residues, which was purified twice by flash column chromatography (hexane:EtOAc, 8:1 → 6:1 → 3:1→ 1:1) to yield an inseparable mixture of vinylstannanes **13*Z***:**S3** (431 mg, 1:4, 8%) as a colourless oil; and vinylstannane **13*E*** (3.25 g, 60%) as a colourless oil; **$[\alpha]_D$** +10.0 (c 1.0, $CHCl_3$); **IR** (neat, cm^{-1}) 3348 (br, OH); **^{1}H NMR** δ (500 MHz, $CDCl_3$) 6.32 (1H, dd, *J* = 19.3, 1.4 Hz, CH=C*H*$SnBu_3$), 5.99 (1H, dd, *J* = 19.3, 5.1 Hz, C*H*=CH$SnBu_3$), 4.27 (1H, appt qd, *J* = 5.0, 1.4 Hz, C*H*OH), 3.68 (1H, ddd, *J* = 11.1, 7.6, 3.6 Hz, C$H_a$$H_b$OH), 3.51 (1H, ddd, *J* = 11.1, 7.4, 4.8 Hz, C$H_a$$H_b$OH), 2.11 (1H, d, *J* = 4.3 Hz, CHO*H*), 1.88 (1H, dd, *J* = 7.2, 4.8 Hz, CH_2O*H*), 1.61-1.37 (6H, m, 3 × CH_2), 1.30 (6H, sxt, *J* = 7.2 Hz, 3 × $CH_2CH_2CH_3$) 1.00-0.79 (15H, m, 3 × CH_2, 3 × CH_3); **^{13}C NMR** δ (126 MHz, $CDCl_3$) 146.1 (CH), 131.0 (CH), 75.5 (CH), 66.4 (CH_2), 29.1 (3 × CH_2), 27.3 (3 × CH_2), 13.7 (3 × CH_3), 9.5 (3 × CH_2); ***m/z*** (ESI+) 401 ($[M+Na]^+$, 21%); **HRMS** (ESI+) $[M+Na]^+$ found 401.1478, $C_{16}H_{34}O_2SnNa$ requires 401.1473.

Selected data for minor stereoisomer **13*Z***:

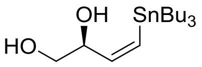

^{1}H NMR δ (400 MHz, $CDCl_3$) 6.50 (1H, dd, J = 13.0, 6.7 Hz, C*H*=$CHSnBu_3$), 6.19 (1H, dd, J = 13.0, 1.0 Hz, CH=C*H*$SnBu_3$), 4.13 (1H, m, C*H*OH), 3.66 (1H, ddd, J = 11.0, 7.2, 4.0 Hz, CH_aH_bOH), 3.54 (1H, ddd, J = 11.0, 5.6, 2.0 Hz, CH_aH_bOH), 2.05 (1H, br s, CHO*H*).

Selected data for major regioisomer **S3**:

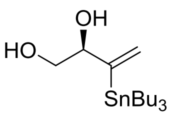

^{1}H NMR δ (400 MHz, $CDCl_3$) 5.93 (1H, dd, J = 2.0, 1.6 Hz, $CSnBu_3$=CH_xH_y), 5.35 (1H, dd, J = 2.0, 1.5 Hz, $CSnBu_3$=CH_xH_y), 4.38 (1H, m, C*H*OH), 3.60 (1H, ddd, J = 11.0, 7.2, 3.8 Hz, CH_aH_bOH), 3.46 (1H, ddd, J = 11.1, 8.2, 4.9 Hz, CH_aH_bOH), 2.21 (1H, br s, CHO*H*).

(2*S*,3*E*)-4-Iodo-3-buten-1,2-diol 12

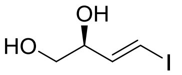

Vinyl stannane **13*E*** (3.24 g, 8.59 mmol) was dissolved in ether (20 mL) and cooled to 0 °C. Iodine (2.84 g, 11.2 mmol) was suspended in ether (17 mL) and then sonicated in an ultrasonic bath for 5 min before being added to the vinyl stannane solution by cannula. After 5 min, TLC (hexane:EtOAc, 2:1) indicated the complete consumption of starting material (R_f 0.3) and the formation of a major product (R_f 0.1). $Na_2S_2O_3$ (2.74 g, 17.3 mmol) and KF (1.01 g, 17.4 mmol) were added and after 2 min, MeOH (10 mL) was added. After a further 10 min, the reaction mixture was concentrated *in vacuo*. The crude product was purified by flash column chromatography (dry load, silica doped with 10% KF, hexane:EtOAc, 2:1 → 1:1 → 0:1) to yield a brown solid (1.85 g). This material was taken up in EtOH (10 mL) and treated with $Na_2S_2O_3$ (13 mL; sat aq), which rapidly removed the brown colour. The mixture was concentrated *in vacuo* and the material was purified again by flash column chromatography (dry load, EtOAc) to yield vinyl iodide **12** (1.68 g, 92%) as a colourless solid; mp 57-59 °C (EtOAc); **$[\alpha]_D$** +27.0 (c 0.27, $CHCl_3$); **IR** (neat, cm^{-1}) 3348 (br s, OH), 1607 (C=C); **^{1}H NMR** δ (500 MHz, $CDCl_3$) 6.62 (1H, dd, J = 14.5, 5.8 Hz, C*H*=CHI), 6.47 (1H, d, J = 14.5, 1.3 Hz, CH=C*H*I), 4.13-4.04 (1H, m, C*H*OH), 3.51 (1H, dd, J = 10.2, 4.3 Hz, CH_aH_bOH), 3.51 (1H, dd, J = 10.2, 5.2 Hz, CH_aH_bOH); **^{13}C NMR** δ (126 MHz, $CDCl_3$) 147.5 (CH), 78.1 (CH), 75.8 (CH), 66.3 (CH_2); ***m/z*** (EI) 214 ($[M]^+$, 2%); **HRMS** (EI) $[M]^+$ found 213.9490, $C_4H_7O_2I$ requires 213.9491.

(2*S*,3*E*)-1-*p*-Methylbenzenesulfonyl-3-hepten-5-yn-2-ol 14

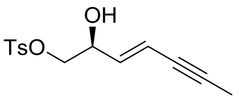

$ZnCl_2$ (1037 mg, 7.46 mmol) was dried under high vacuum using a heat gun (3 blasts over a period of 40 min) and then suspended in THF (4 mL) under Ar and cooled to 0 °C. After 15 min, 1-propynylmagnesium bromide (13.0 mL, 6.50 mmol) was added portionwise which resulted in a white suspension. After a further 15 min, vinyl iodide **12** (259 mg, 1.21 mmol) was dissolved in THF (5 mL) and added to the $ZnCl_2$ suspension by cannula (rinsing with 2 mL THF), followed by $PdCl_2(PPh_3)_2$ (53 mg, which resulted in a pale yellow suspension. After 5 min, the cooling was removed and the reaction was allowed to warm to rt. After an additional 24 h 50 min, NH_4Cl (350 mg, 6.54 mmol) was added and the reaction mixture was concentrated *in vacuo*. The crude product was purified by flash column chromatography (dry load, hexane:EtOAc, 1:1 → 0:1) to yield enyne diol **S4** as a black oil (800 mg), which was used without further purification; **^{1}H NMR** δ (500 MHz, CD_3OD) 5.99 (1H, ddd, J = 15.9, 5.9, 0.5 Hz, CHOHC*H*=), 5.77-5.69 (1H, m, C*H*C≡CCH_3), 4.11 (1H, appt q, J = 5.2 Hz, C*H*OH), 3.50 (1H, dd, J = 11.1, 5.0 Hz, CH_aH_bOH), 3.46 (1H, dd, J = 11.1, 6.6 Hz, CH_aH_bOH), 1.90 (3H, d, J = 2.3 Hz, C≡CCH_3); **^{13}C NMR** δ (126 MHz, $CDCl_3$) 142.1 (CH), 112.6 (CH), 87.0 (C), 78.5 (C), 73.7 (CH),

66.9 (CH_2), 3.7 (CH_3); ***m/z*** (ESI+) 149 ($[M+Na]^+$, 87%); **HRMS** (ESI+) $[M+Na]^+$ found 149.0568, requires $C_7H_{10}O_2Na$ 149.0573.

Diol **S4** (800 mg, assumed 1.21 mmol) was suspended in CH_2Cl_2 (8 mL) under Ar and cooled to 0 °C. Bu_2SnO (30 mg, 0.12 mmol) was added and after 15 min, tosyl chloride (230 mg, 1.21 mmol) was added. After an additional 2 min, NEt_3 (170 µL, 1.23 mmol) was added portionwise over a period of 5 min. The reaction vessel was covered in aluminium foil to shut out light, cooling was removed and the reaction was allowed to warm to rt. After 11 h, TLC (hexane:EtOAc, 1:1) indicated the complete consumption of starting material (R_f 0.1) and the formation of a major product (R_f 0.8). The reaction mixture was concentrated *in vacuo* and the resulting crude product was purified by column chromatography (dry load, hexane:EtOAc, 6:1 → 5:1→ 4:1 → 2:1 → 1:1) to yield mono-tosylate **14** (205 mg, 60% over 2 steps) as a yellow oil; **[α]D** +21.0 (*c* 1.0, $CHCl_3$); **IR** (neat, cm^{-1}) 3501 (br, OH), 1597 (C=C); **^{1}H NMR** δ (500 MHz, $CDCl_3$) 7.82 (2H, d, *J* = 8.2 Hz, 2 × Ar*H*), 7.38 (2H, d, *J* = 8.2 Hz, 2 × Ar*H*), 5.89 (1H, dd, *J* = 16.0, 5.4 Hz, CHOHC*H*=), 5.83-5.76 (1H, m, C*H*C≡CCH_3), 4.48-4.40 (1H, m, C*H*OH), 4.06 (1H, dd, *J* = 10.4, 3.4 Hz, CH_aH_bOTs), 3.91 (1H, dd, *J* = 10.4, 7.5 Hz, CH_aH_bOTs), 2.48 (3H, s, ArC*H₃*), 1.96 (3H, d, *J* = 2.2 Hz, C≡CCH_3), 1.94 (1H, br s, O*H*); **^{13}C NMR** δ (126 MHz, $CDCl_3$) 145.2 (C), 136.9 (CH), 132.6 (C), 130.0 (2 × CH), 128.0 (2 × CH), 113.9 (CH), 88.4 (C),* 72.6 (CH_2), 69.8 (CH), 21.7 (CH_3), 4.3 (CH_3); ***m/z*** (ESI+) 583 ($[2M+Na]^+$, 26%), 303 ($[M+Na]^+$, 100); **HRMS** (ESI+) $[M+Na]^+$ found 303.0662, $C_{14}H_{16}O_4SNa$ requires 303.0662.
* one alkyne C obscured by $CDCl_3$

(3*R*,4*E*)-3-Methoxy-4-oct-en-6-ynenitrile S5

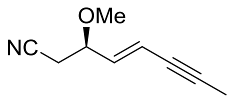

Alcohol **14** (205 mg, 0.73 mmol) was dissolved in CH_2Cl_2 (15 mL) under Ar and trimethyloxoniumtetrafluroborate (284 mg, 1.82 mmol) was added, which resulted in the solution changing from pale yellow to brown. After 1 min, 1,8-bis(dimethylamino)naphthalene, proton sponge, (472 mg, 2.20 mmol) was added and the reaction mixture turned yellow again. After 1 h 25 min, TLC (hexane:EtOAc, 2:1) indicated the presence of some starting material (R_f 0.4) and the formation of a major product (R_f 0.6). After an additional 35 min, the reaction mixture was filtered and concentrated *in vacuo*. The resulting crude product was purified by column chromatography (dry load, hexane:EtOAc, 6:1 → 5:1 → 4:1→ 2:1) to yield slightly impure methyl ether **15** (211 mg) as a light yellow oil, which solidified in the freezer; **[α]D** +68.0 (*c* 1.0, $CHCl_3$); **IR** (neat, cm^{-1}) 1597 (C=C); **^{1}H NMR** δ (500 MHz, $CDCl_3$) 7.80 (2H, *J* = 8.1 Hz, 2 × Ar*H*), 7.36 (2H, d, *J* = 8.1 Hz, 2 × Ar*H*), 5.75 (1H, dd, *J* = 16.0, 5.8 Hz, CHOHC*H*=), 5.70 (1H, dq, *J* = 16.0, 1.8 Hz, C*H*C≡CCH_3), 3.99 (1H, dd, *J* = 10.4, 4.5 Hz, CH_aH_bOTs), 3.96 (1H, dd, *J* = 10.4, 6.4 Hz, CH_aH_bOTs), 3.88 (1H, appt dt, *J* = 10.7, 5.4 Hz, C*H*OMe), 3.26 (3H, s, OCH_3), 2.46 (3H, s, ArCH_3), 1.95 (3H, d, *J* = 1.8 Hz, C≡CCH_3); **^{13}C NMR** δ (126 MHz, $CDCl_3$) 145.0 (C), 136.1 (CH), 133.0 (C), 129.9 (2 × CH), 128.1 (2 × CH), 115.5 (CH), 88.4 (C), 79.1 (CH),* 71.1 (CH_2), 57.1 (CH_3), 21.7 (CH_3), 4.3 (q, CH_3); ***m/z*** (ESI+) 591 ($[2M+H]^+$, 7%), 317 ($[M+Na]^+$, 100), 295 ($[M+H]^+$, 5); **HRMS** (ESI+) $[M+H]^+$ found 295.0994, $C_{15}H_{19}O_4S$ requires 295.0999.
* one alkyne C obscured by $CDCl_3$

WARNING: The next reaction involves the highly toxic cyanide ion. The potassium cyanide should be weighed out in a fumehood with a HCN detector and a respirator close by. All relevant glassware and aqueous solutions were treated with bleach (NaClO) overnight to render harmless any remaining cyanide.

Tosylate **15** (211 mg) was dissolved in DMSO (2 mL) under Ar and $NaHCO_3$ (187 mg, 2.23 mmol), tetrabutylammonium iodide (55 mg, 0.15 mmol) and potassium cyanide (100 mg, 1.54 mmol) were

added in sequence and the resulting yellow suspension was heated to 60 °C. After 2 h 20 min, TLC (toluene:EtOAc, 10:1) indicated the presence of starting material (R_f 0.5) and the formation of product (R_f 0.4). The reaction mixture was heated to 70 °C and after a further 1 h 40 min, TLC now indicated complete consumption of starting material and the formation of a major product. The reaction mixture was allowed to cool to rt and then diluted with ether (50 mL) and washed with $NaHCO_3$ (50 mL; sat aq). The aqueous phase was re-extracted with ether (3 × 25 mL). The organic phases were combined, washed with brine (50 mL), dried ($MgSO_4$), filtered and concentrated *in vacuo*. The resulting crude product was purified by flash column chromatography (toluene:EtOAc, 1:0 → 30:1→ 20:1→ 10:1) to afford nitrile **S5** (69 mg, 63% over 2 steps) as a pale yellow oil; **[α]D** +46.0 (c 1.0, $CHCl_3$); **IR** (neat, cm^{-1}) 2251 (C≡N), 2224 (C≡C), 1634 (C=C); **^{1}H NMR** δ (500 MHz, $CDCl_3$) 5.88 (1H, ddd, *J* = 16.2, 7.4, 0.4 Hz, CHOMeC*H*=), 5.79 (1H, dq, *J* = 16.2, 2.1 Hz, C*H*C≡CCH_3), 3.88 (1H, appt q, *J* = 6.5 Hz, C*H*OMe), 3.33 (3H, s, OCH_3), 2.57 (2H, d, *J* = 6.0 Hz, CH_2CN),1.96 (3H, d, *J* = 2.1 Hz, $CCCH_3$); **^{13}C NMR** δ (126 MHz, $CDCl_3$) 137.8 (CH), 116.8 (CN), 115.6 (CH), 88.8 (C), 77.0 (CH), 76.7 (C), 56.9 (CH_3), 24.6 (CH_2), 4.3 (CH_3); ***m/z*** (EI) 149 ($[M]^+$, 6%); **HRMS** (EI) $[M]^+$ found 149.0836, $C_9H_{11}NO$ requires 149.0841.

(3*R*,4*E*)-3-methoxy-oct-4-en-6-ynoic acid 16

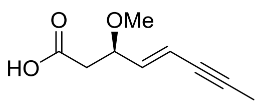

Nitrile **S5** (310 mg, 2.08 mmol) was dissolved in EtOH (8 mL) and H_2O_2 (9 mL; 30% aq) and $LiOH•H_2O$ (378 mg, 9.0 mmol) were added. After 34 h, TLC (EtOAc with 5% AcOH) indicated the complete consumption of starting material (R_f 0.9) and the formation of a major (R_f 0.7) and minor product (R_f 0.5). The reaction mixture was cooled to 0 °C and sodium bisulfite (10 mL, sat aq) was added carefully in portions to quench the remaining H_2O_2. After 15 min, the resulting mixture was poured into a separatory funnel containing sodium bisulfite (20 mL, sat aq) and extracted with EtOAc (6 × 30 mL). The combined organic phases were dried ($MgSO_4$), filtered and concentrated *in vacuo*. The resulting crude product was purified by flash column chromatography (dry load, hexane:EtOAc, 1:1 → 1:3 → 0:1→ EtOAc:EtOH, 10:1 with 0.5% AcOH) to afford carboxylic acid **16** (264 mg, 75%) as a colourless solid; mp 55-57 °C (CH_2Cl_2); **[α]D** +64.0 (c 1.0, $CHCl_3$); **IR** (neat, cm^{-1}) 3339 (br, OH), 1667 (C=O); **^{1}H NMR** δ (400 MHz, $CDCl_3$) 9.69 (1H, br s, CO_2H), 5.91 (1H, dd, *J* = 15.9, 7.6 Hz, CHOMeC*H*=), 5.74 (1H, dq, *J* = 15.9, 2.1 Hz, C*H*C≡CCH_3), 4.07 (1H, appt td, *J* = 7.9, 4.9 Hz, C*H*OMe), 3.33 (3H, s, OCH_3), 2.63 (1H, dd, *J* = 15.7, 8.4 Hz, $HO_2CCH_aH_b$), 2.54 (1H, dd, *J* = 15.7, 4.9 Hz, $HO_2CCH_aH_b$), 1.95 (3H, d, *J* = 2.1 Hz, $CCCH_3$); **^{1}H NMR** δ (500 MHz, CD_3OD) δ 5.85 (1H, dd, *J* = 15.9, 7.5 Hz, CHOMeC*H*=), 5.73 (1H, dq, *J* = 15.9, 2.2 Hz, C*H*$CCCH_3$), 4.06 (1H, td, *J* = 7.7, 5.4 Hz, C*H*OMe), 3.28 (3H, s, OCH_3), 2.47 (1H, dd, *J* = 14.3, 8.2 Hz, $HO_2CCH_aH_b$), 2.45 (dd, *J* = 14.3, 5.4 Hz, $HO_2CCH_aH_b$), 1.93 (3H, d, *J* = 2.2 Hz, C≡CCH_3); **^{13}C NMR** δ (126 MHz, $CHCl_3$) 175.9 (CO), 139.4 (CH), 113.9 (CH), 87.8 (C), 77.8 (CH),* 56.7 (CH_3), 40.6 (CH_2), 4.3 (CH_3); **^{13}C NMR** δ (126 MHz, CD_3OD) 175.6 (CO), 141.1 (CH), 114.5 (CH), 87.8 (C), 79.8 (CH), 78.0 (C), 56.9 (CH_3), 42.8 (CH_2), 3.7 (CH_3); ***m/z*** (ESI+) 190 ($[M+Na]^+$, 63%); *m/z* (ESI–) 167 ($[M-H]^-$, 49%); **HRMS** (ESI–) $[M-H]^-$ found 167.0716, $C_9H_{11}O_3$ requires 167.0714.

* one alkyne C obscured by $CDCl_3$

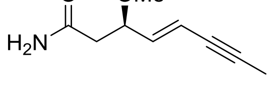

and amide **S6** (28 mg, 8%) as a colourless solid; **^{1}H NMR** δ (500 MHz, $CDCl_3$) 6.18 (1H, br s, N*H*), 5.90 (1H, dd, *J* = 15.9, 7.6 Hz, CHOMeC*H*=), 5.70 (1H, dq, *J* = 15.9, 2.2 Hz, C*H*C≡CCH_3), 5.55 (1H, br s, N*H*), 4.02 (1H, appt td, *J* = 7.8, 4.1 Hz, C*H*OMe), 3.33 (3H, s, OCH_3), 2.48 (1H, dd, *J* = 15.1, 8.1 Hz, H_2NCOCH_aH-b), 2.43 (1H, dd, *J* = 15.1, 4.0 Hz, $H_2NCOCH_aH_b$), 1.97 (3H, d, *J* = 2.2 Hz, C≡CCH_3); **^{13}C NMR** δ (126 MHz, $CDCl_3$) 172.6 (CO), 139.6 (CH), 113.7 (CH), 87.7 (C), 78.4 (CH),* 56.7 (CH_3), 42.2 (CH_2), 4.3 (CH_3). * one alkyne C obscured by $CDCl_3$

(2*S*)-3-hydroxy-2-[(3'*R*,4'*E*)-3'-methoxy-4'-octen-6'-ynamido]propionic acid methyl ester S7

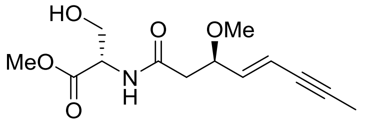

Carboxylic acid **16** (25 mg, 0.15 mmol) was dissolved in CH_3CN (2 mL) and cooled to 0 °C. After 10 min, *N,N,N',N'*-tetramethyl-*O*-(1*H*-benzotriazol-1-yl)uronium hexafluorophosphate (25 mg, 0.16 mmol) and ethyldiisopropylamine (50 µL, 0.29 mmol) were added. After an additional 25 min, serine methyl ester hydrochloride (25 mg, 0.16 mmol) was added and the reaction mixture was slowly allowed to warm to rt. After 9.5 h, TLC (hexane:EtOAc, 1:2 with ~ 2% AcOH) indicated the complete consumption of starting acid (R_f 0.6) and the formation of a major product (R_f 0.3). The reaction mixture was transferred to a flask containing silica and concentrated *in vacuo*. The crude product was purified twice by flash column chromatography (dry load, hexane:EtOAc, 1:2→ 0:1) and (dry load, hexane:EtOAc, 1:1→ 1:2 → 0:1) and filtered through a plug of cotton wool using chloroform as the solvent to yield **S7** (33 mg, 82%) as a colourless solid; **mp** 73-75 °C ($CDCl_3$); **[α]D** +62.0 (c 1.0, $CHCl_3$); **IR** (neat, cm^{-1}) 3347 (OH), 1742 (s, C=O), 1649 (s, C=ON), 1530 (C=O); **^{1}H NMR** δ (400 MHz, $CDCl_3$) 7.21 (1H, d, *J* = 7.2 Hz, N*H*), 5.90 (1H, ddd, *J* = 15.9, 7.5, 0.6 Hz, CHOMeC*H*=), 5.72 (1H, dqd, *J* = 15.9, 2.1, 0.8 Hz, C*H*C≡CCH3), 4.68 (1H, dt, *J* = 7.2, 3.7 Hz, C*H*CH2OH), 4.03 (1H, appt td, *J* = 8.0, 4.1 Hz, C*H*OMe), 3.96 (2H, br s, C*H2*OH), 3.82 (3H, s, CO2C*H3*), 3.35 (3H, s, OC*H3*), 2.65 (1H, br s, O*H*), 2.55 (1H, dd, *J* = 14.9, 8.3 Hz, HNCOC*Ha*Hb), 2.45 (1H, dd, *J* = 14.9, 4.1 Hz, HNCOCHa*Hb*), 1.97 (3H, d, *J* = 2.1 Hz, C≡CCH3); **^{13}C NMR** δ (126 MHz, $CDCl_3$)170.9 (CO), 170.8 (CO), 139.6 (CH), 113.6 (CH), 87.8 (C), 78.5 (CH),* 63.2 (CH_2), 56.7 (CH_3), 54.8 (CH), 52.7 (CH_3), 42.3 (CH_2), 4.2 (CH_3); ***m/z*** (ESI+) 561 ($[2M+Na]^+$, 5%), 291 ($[M+Na]^+$, 100); **HRMS** (ESI+) $[M+Na]^+$ found 292.1183, $C_{13}H_{19}NO_5Na$ requires 292.1155.
* one alkyne C obscured by $CDCl_3$

2-[(2'*R*,3'*E*)-2'-methoxy-3'-hepten-5'-yn-1'-yl]oxazole-4-carboxylic acid methyl ester 17

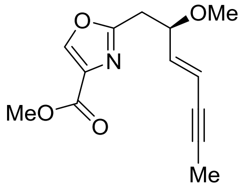

Hydroxyamide **S7** (71 mg, 0.26 mmol) was dissolved in CH_2Cl_2 (5 mL) under Ar and cooled to -78 °C. After 10 min, XtalFluor (71 mg, 0.31 mmol) was added and after a further 30 min, anhydrous K_2CO_3 (51 mg, 0.37 mmol) was added. After an additional 40 min, the reaction mixture was warmed to 0 °C in an ice-water bath. TLC (EtOAc) indicated the complete consumption of starting material (R_f 0.6) and the formation of a major product (R_f 0.8). $NaHCO_3$ (15 mL; sat aq) was added portionwise to the reaction mixture, which was allowed to warm to rt. The mixture was then transferred to a separatory funnel and extracted with CH_2Cl_2 (3 × 15 mL). The combined organic phases were dried ($MgSO_4$), filtered and concentrated *in vacuo*. The crude product was dried under high vacuum for 30 min, re-dissolved in CH_2Cl_2 (5 mL) and cooled to -20 °C. After 15 min, $BrCCl_3$ (40 µL, 0.41 mmol) and DBU (45 µL, 0.32 mmol) were added and the resulting mixture was slowly allowed to warm to rt. After 4h 45 min, TLC (hexane:EtOAc, 3:1) indicated the complete consumption of the intermediate oxazoline (R_f 0.1) and the formation of a major product (R_f 0.3). NH_4Cl (20 mL; sat aq) was added to the reaction mixture, which was then poured into a separatory funnel and extracted with CH_2Cl_2 (3 × 20 mL). The combined organic phases were dried ($MgSO_4$), filtered and concentrated *in vacuo*. The crude product was purified by flash column chromatography (hexane:EtOAc, 3:1→ 2:1 → 1:1) to yield **17** (50 mg, 76%) as a colourless oil; **[α]D** –37.0 (c 1.0, $CHCl_3$); **IR** (neat, cm^{-1}) 1744 (s, C=O), 1584 (C=C); **^{1}H NMR** δ (500 MHz, $CDCl_3$) 8.17 (1H, s, Ar*H*), 5.91 (1H, dd, *J* = 15.9, 7.7 Hz, CHOMeC*H*=), 5.69 (1H, dq, *J* = 15.9, 2.0 Hz, C*H*C≡CCH3), 4.13 (1H, appt td, *J* = 7.7, 5.8 Hz, C*H*OMe), 3.91 (3H, s, CO2C*H3*), 3.26 (3H, s, OC*H3*), 3.09 (1H, dd, *J* = 15.0, 7.7 Hz, C*Ha*HbCHOMe), 2.98 (1H, dd, *J* = 15.0, 5.8 Hz, CHa*Hb*CHOMe), 1.94 (3H, d, *J* = 2.0 Hz, C≡CC*H3*); **^{13}C NMR** δ (126 MHz, $CDCl_3$) 162.5 (C), 161.7

(C), 144.0 (CH), 139.6 (CH), 133.4 (C), 114.2 (CH), 87.8 (C), 79.3 (CH),* 56.7 (CH_3), 52.1 (CH_3), 34.6 (CH_2), 4.3 (CH_3); ***m/z*** (ESI+) 521 ($[2M+Na]^+$, 78%), 272 ($[M+Na]^+$, 100), 250 ($[M+H]^+$, 43); **HRMS** (ESI+) $[M+Na]^+$ found 272.0916, $C_{13}H_{15}NO_4Na$ requires 272.0893; $[M+H]^+$ found 250.1088, $C_{13}H_{16}NO_4$ requires 250.1074.
* one alkyne C obscured by $CDCl_3$

2-[(2'*R*,3'*E*)-2'-methoxy-3'-hepten-5'-yn-1'-yl]oxazole-4-carboxylic acid 28

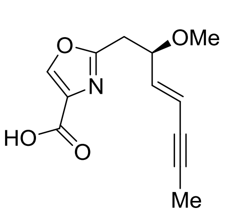

Methyl ester **16** (22 mg, 0.088 mmol) was dissolved in THF (1.5 mL) and LiOH (0.25 mL, 0.25 mmol; 1 M aq) was added. After 8 h, TLC (hexane:EtOAc, 1:1) indicated the complete consumption of starting material (R_f 0.7) and the formation of a minor ($R_f \sim$ 0.9) and major product ($R_f \sim$ 0). The reaction mixture was diluted with EtOAc (10 mL) and was poured into a separatory funnel containing HCl (0.1 M, 10 mL). The aqueous phase was re-extracted with EtOAc (5 × 10 mL) and the combined organic phases were dried ($MgSO_4$), filtered and concentrated *in vacuo* to yield acid **28** (20 mg, 96%) as a colourless solid; R_f 0.6 (EtOAc:AcOH, 2:1); mp 117-120 °C; **[α]D** −33.0 (c 1.0, $CHCl_3$); **IR** (neat, cm^{-1}) 1722 (s, C=O), 1587 (C=N); **^{1}H NMR** δ (500 MHz, $CDCl_3$) 8.30 (1H, s, Ar*H*), 7.31 (1H, br s, CO_2*H*), 5.93 (1H, dd, *J* = 15.8, 7.4 Hz, CHOMeC*H*=), 5.72 (1H, dq, *J* = 15.8, 2.1 Hz, C*H*C≡CCH_3), 4.27-4.13 (1H, m, C*H*OMe), 3.26 (3H, s, OCH_3), 3.21-2.96 (2H, m, CH_2CHOMe), 1.96 (3H, d, *J* = 2.1 Hz, C≡CCH_3); **^{13}C NMR** δ (126 MHz, $CDCl_3$) 164.8 (C), 162.9 (C), 145.0 (CH), 139.5 (CH), 132.8 (C), 114.3 (CH), 87.9 (C), 79.2 (CH),* 56.7 (CH_3), 34.5 (CH_2), 4.3 (CH_3); ***m/z*** (ESI+) 493 ($[2M+Na]^+$, 13%), 258 ($[M+Na]^+$, 100), 236 ($[M+H]^+$, 38); **HRMS** (ESI+) $[M+Na]^+$ found 258.0739, $C_{12}H_{13}NO_4Na$ requires 258.0737; $[M+H]^+$ found 236.0913, $C_{12}H_{14}NO_4$ requires 236.0917.
* one alkyne C obscured by $CDCl_3$

Synthesis of β-hydroxy ketone fragment 5

(*S*)-3-Hydroxy-5-(4-methoxybenzyloxy)-2,2-dimethylpentanoic acid methyl ester 19

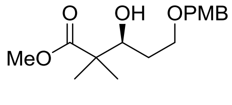

Alcohol **19** was prepared according to a modification of the procedure described by Hartung.[6b] BH_3•THF (106 mL, 106 mmol; 1.0 M in THF) was added dropwise over 15 min to a suspension of *N*-Ts-*D*-valine (28.8 g, 106 mmol) [CAUTION: gases evolved] in DCM (500 mL) at rt and the solution was stirred until gas evolution ceased (~1.5 h) before cooling of the reaction mixture to –78 °C. Aldehyde **18** (19.4 g, 100 mmol) in DCM (300 mL) was added at such a rate as to maintain the internal temperature below –70 °C (~15 min) before dropwise addition (30 min) of 1-methoxy-1-(trimethylsilyloxy)-2-methylprop-1-ene (30.5 g, 175 mmol) in DCM (100 mL). The solution was stirred for 5.5 h, quenched by addition of phosphate buffer solution (500 mL; pH 7) and the reaction mixture was warmed to rt. The aqueous layer was extracted with DCM (3 × 100 mL), and the combined organic layers were washed with brine (400 mL), dried ($MgSO_4$) and the solvent was removed under reduced pressure. The yellow oil thus obtained was dissolved in THF (100 mL) and HCl (100 mL; 5.0 M aq), the solution was stirred for 3 h at rt, and the aqueous layer was extracted with Et_2O (4 × 100 mL). The combined organic layers were washed with NaOH (2 × 100 mL; 1.0 M aq), $NaHCO_3$ (100 mL; sat aq), brine (100 mL) and dried ($MgSO_4$), and the solvent was removed under reduced pressure. The combined aqueous layers from both aqueous workups were acidified to pH 1 by addition of HCl (200 mL; conc aq),

extracted with EtOAc (3 × 400 mL), washed with brine (400 mL), dried ($MgSO_4$) and the solvent was removed under reduced pressure. Two recrystallisations (EtOH/H_2O) of the resulting solid gave *N*-Ts-*D*-valine as colourless needles (17.2 g, 60% recovery). Purification of the resulting pale-yellow oil (obtained from the Et_2O layers) by flash chromatography (Hexane:EtOAc, 3:1) gave β-hydroxyester **19** as a light-yellow oil (24.0 g, 85%, 89 %*ee*). N.B. Repeated runs typically gave comparable yield (75–85%) and enantioselectivity (88–89 %*ee*). $\mathbf{R_f}$ (Hexane:EtOAc, 3:1) = 0.36; $\mathbf{R_t}$ (Method B) = 30.5 [(*S*)-isomer], 33.5 [(*R*)-isomer]; $\mathbf{[\alpha]_D}$ = –1.50 (*c* 2.52, $CHCl_3$), lit[6] $[\alpha]_D$ = –1.04 (*c* 1.15, $CHCl_3$); **IR** (neat, cm^{-1}) 3516 (OH), 1726 (C=O), 1612 (C=C), 1585 (C=C), 1512 (C=C); $\mathbf{^1H}$ **NMR** δ (500 MHz, $CDCl_3$) 7.28–7.27 (2H, m, 2Ar*H*), 6.91–6.88 (2H, m, 2Ar*H*), 4.49 (1H, d, *J* = 11.4 Hz, OCH_AH_BAr), 4.46 (1H, d, *J* = 11.4 Hz, OCH_AH_BAr), 3.93 (1H, dt, *J* = 8.7, 3.8 Hz, C*H*OH), 3.83 (3H, s, $ArOCH_3$), 3.75–3.72 (1H, m, $CH_XH_YOCH_2Ar$), 3.72 (3H, s, CO_2CH_3), 3.69–3.65 (1H, m, $CH_XH_YOCH_2Ar$), 3.23 (1H, d, *J* = 3.8 Hz, O*H*), 1.74–1.68 (2H, m, CH_2CHOH), 1.21 (3H, s, CCH_3), 1.20 (3H, s, CCH_3); $\mathbf{^{13}C}$ **NMR** δ (125 MHz, $CDCl_3$) 177.8 (C), 159.3 (C), 130.1 (C), 129.4 (2CH), 113.8 (2CH), 75.7 (CH), 73.0 (CH_2), 69.1 (CH_2), 55.3 (CH_3), 51.9 (CH_3), 47.0 (C), 31.4 (CH_2), 21.7 (CH_3), 20.4 (CH_3); ***m/z*** (EI) 296 ($[M]^+$, 1%), 142 (15), 137 (47), 135 (19), 121 (100). The spectroscopic data are in good agreement with the literature.[6]

(*S*)-3-(*tert*-Butyldimethylsilanyloxy)-5-(4-methoxybenzyloxy)-2,2-dimethylpentanoic acid methyl ester 20

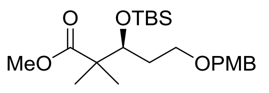

Cautiously, TBSOTf (13.9 mL, 56.5 mmol) was added to a solution of β-hydroxyester **19** (10.9 g, 38.3 mmol) and 2,6-lutidine (8.92 mL, 76.6 mmol) in DCM (120 mL), and the reaction mixture was stirred for 2.5 h at –78 °C, quenched with $NaHCO_3$ (200 mL; sat aq) [CAUTION: gases evolved] and warmed to rt. The aqueous layer was extracted with DCM (3 × 50 mL), and the combined organic layers were washed with $NaHCO_3$ (100 mL; sat aq), H_2O (100 mL), brine (100 mL), and dried ($MgSO_4$). The solvent and residual 2,6-lutidine were removed under reduced pressure (bath temperature = 70 °C), and the crude material was purified by flash chromatography (Hexane:EtOAc, 3:1) to give TBS-ether **20** as a colourless oil (14.8 g, 94%). N.B. Provided residual 2,6-lutidine is completely removed, the crude product may be taken forward to the subsequent PMB-deprotection step without chromatography. $\mathbf{R_f}$ (Hexane:EtOAc, 3:1) = 0.72; $\mathbf{[\alpha]_D}$ = –4.20 (*c* 1.17, $CHCl_3$), lit[6] $[\alpha]_D$ = –4.20 (*c* 1.02, $CHCl_3$); **IR** (neat, cm^{-1}) 1732 (C=O), 1612 (C=C), 1585 (C=C), 1512 (C=C); $\mathbf{^1H}$ **NMR** δ (500 MHz, $CDCl_3$) 7.30–7.27 (2H, m, 2Ar*H*), 7.92–7.90 (2H, m, 2Ar*H*), 4.46 (1H, d, *J* = 11.5 Hz, CH_AH_BAr), 4.43 (1H, d, *J* = 11.5 Hz, CH_AH_BAr), 4.07 (1H, dd, *J* = 7.7, 3.1 Hz, C*H*OSi), 3.83 (3H, s, $ArOCH_3$), 3.66 (3H, s, CO_2CH_3), 3.54–3.46 (2H, m, CH_2OCH_2Ar), 1.84–1.78 (1H, m, CH_XH_YCHOSi), 1.70–1.63 (1H, m, CH_XH_YOSi), 1.18 (3H, s, CCH_3), 1.11 (3H, s, CCH_3), 0.88 (9H, s, $3SiC(CH_3)_3$), 0.06 (3H, s, $SiCH_3$), 0.03 (3H, s, $SiCH_3$); $\mathbf{^{13}C}$ **NMR** δ (125 MHz, $CDCl_3$) 177.5 (C), 159.2 (C), 130.6 (C), 129.2 (2CH), 113.8 (2CH), 73.9 (CH), 72.5 (CH_2), 67.4 (CH_2), 55.3 (CH_3), 51.7 (CH_3), 48.4 (C), 33.9 (CH_2), 26.0 ($3CH_3$), 21.5 (CH_3), 20.5 (CH_3), 18.3 (C), –4.0 (CH_3), –4.3 (CH_3); ***m/z*** (ESI+, MeOH) 433 ($[M+Na]^+$, 100%). The spectroscopic data are in good agreement with the literature.[6]

(*S*)-3-(*tert*-Butyldimethylsilanyloxy)-5-hydroxy-2,2-dimethylpentanoic acid methyl ester S8

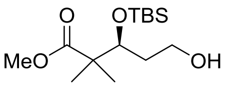

DDQ (25.7 g, 113 mmol) was added in 5 portions (5 × 5.14 g) over 10 min to a solution of PMB-ether **20** (31.0 g, 75.5 mmol; telescoped) in DCM (302 mL) and H_2O (17 mL) at 0 °C and the red-brown mixture was stirred for 1.5 h. The reaction was quenched by cautious addition of $NaHCO_3$ (400 mL; sat aq) [CAUTION: potential for HCN gas evolution; careful, controlled extrusion is required] and stirred until gas evolution ceased (~1 h). The black emulsion was poured into H_2O (3 dm^3), and the aqueous layer was extracted with DCM (5 × 500 mL). The combined organic layers were washed with $NaHCO_3$ (1 dm^3; sat aq), H_2O (1 dm^3), brine (1 dm^3) and dried ($MgSO_4$). The solvent was removed under reduced pressure and the brown oil thus obtained was purified by flash chromatography (DCM:MeOH, 50:1) to give alcohol **S8** as a brown oil (21.9 g, quant). $\mathbf{R_f}$ (DCM:MeOH, 50:1) = 0.32; $[\alpha]_D$ = –1.80 (*c* 1.10, $CHCl_3$); **IR** (neat, cm^{-1}) 3468 (OH), 1720 (C=O); **^{1}H NMR** δ (500 MHz, $CDCl_3$) 4.11 (1H, dd, *J* = 6.9, 4.3 Hz, C*H*OSi), 3.75–3.66 (2H, m, CH_2OH), 3.70 (3H, s, OCH_3), 1.75–1.66 (2H, m, CH_2CHOSi), 1.52 (1H, br s, O*H*), 1.21 (3H, s, CCH_3), 1.14 (3H, s, CCH_3), 0.91 (9H, s, 3SiC(CH_3)$_3$), 0.13 (3H, s, SiCH_3), 0.08 (3H, s, SiCH_3); **^{13}C NMR** δ (125 MHz, $CDCl_3$) 177.8 (C), 73.6 (CH), 60.1 (CH_2), 51.8 (CH_3), 48.1 (C), 36.9 (CH_2), 26.0 (3CH_3), 22.4 (CH_3), 19.8 (CH_3), 18.3 (C), –4.0 (CH_3), –4.3 (CH_3); ***m/z*** (ESI+, MeOH/DCM) 314 ($[M+Na]^+$, 100%), 292 ($[M+H]^+$, 4%); **HRMS** (ESI+, MeOH/DCM) $[M+H]^+$ found 291.1985, $C_{14}H_{31}O_4Si$ requires 291.1986.

(*S*)-3-(*tert*-Butyldimethylsilanyloxy)-2,2-dimethyl-5-oxo-pentanoic acid methyl ester S9

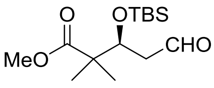

DMSO (63.9 mL, 900 mmol) was added dropwise over 15 min to a solution of $(COCl)_2$ (11.4 mL, 134 mmol) in DCM (100 mL) and the solution was stirred for 15 min at –78 °C [CAUTION: gases evolved]. Alcohol **S8** (19.5 g, 67.1 mmol) in DCM (100 mL) was added dropwise over 25 min [CAUTION: gases evolved] and the mixture was stirred for 1 h before cautious addition (~15 min) of freshly distilled triethylamine (74.8 mL, 537 mmol). The slurry thus obtained was warmed to rt (~1 h) and the reaction was quenched with H_2O (250 mL). The aqueous layer was extracted with DCM (3 × 50 mL), and the combined organic layers were washed with H_2O (3 × 250 mL), brine (250 mL) and dried ($MgSO_4$). The solvent and the Me_2S byproduct were removed under reduced pressure [CAUTION: toxic, malodorous] and the orange residual oil thus obtained was purified by flash chromatography (Hexane:EtOAc, 6:1) to give gave aldehyde **S9** as a yellow oil (18.4 g, 95%). $\mathbf{R_f}$ (Hexane:EtOAc, 6:1) = 0.47; $[\alpha]_D$ = +11.2 (*c* 0.80, $CHCl_3$); **IR** (neat, cm^{-1}) 2723 (CHO), 1725 (C=O); **^{1}H NMR** δ (500 MHz, $CDCl_3$) 9.83 (1H, dd, *J* = 2.4, 1.6 Hz, C*H*O), 4.57 (1H, dd, *J* = 5.8, 4.7 Hz, C*H*OSi), 3.69 (3H, s, OCH_3), 2.62 (1H, ddd, *J* = 17.2, 4.7, 1.6 Hz, CH_AH$_B$), 2.57 (1H, ddd, *J* = 17.2, 5.8, 2.4 Hz, CH$_A$$H_B$), 1.22 (3H, s, CC$H_3$), 1.14 (3H, s, CC$H_3$), 0.88 (9H, s, 3SiC(C$H_3$)$_3$), 0.10 (3H, s, SiC$H_3$), 0.05 (3H, s, SiC$H_3$); **^{13}C NMR** δ (125 MHz, $CDCl_3$) 201.1 (CH), 176.8 (C), 71.5 (CH), 51.9 (CH_3), 48.4 (CH_2), 48.1 (C), 25.8 (3CH_3), 21.3 (CH_3), 20.7 (CH_3), 18.1 (C), –4.1 (CH_3), –4.9 (CH_3); ***m/z*** (ESI+, MeOH/DCM) 343 ($[M+MeOH+Na]^+$, 100%), 311 ($[M+Na]^+$, 62%); **HRMS** (ESI+, MeOH/DCM) $[M+Na]^+$ found 311.1643, $C_{14}H_{28}O_4NaSi$ requires 311.1649.

(3*S*,5*Z*)-3-(*tert*-Butyldimethylsilanyloxy)-6-iodo-2,2-dimethylhex-5-enoic acid methyl ester 21

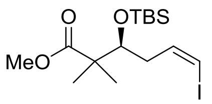

Vinyl iodide **21** was prepared according to a modification of Stork and Zhao's iodo-olefination procedure.[7] NaHMDS (58.4 mL, 126 mmol; 2.0 M in THF) was added to a suspension of freshly prepared $[Ph_3PCH_2I]^+I^-$ (67.0 g, 126 mmol) in THF (365 mL) and the red solution was stirred for 5 min before cooling to –78 °C. HMPA (43.9 mL, 243 mmol) was added, and the solution was stirred for a further 5 min before aldehyde **S9** (18.2 g, 63.2 mmol) in THF (60 mL) was added dropwise (15 min). The reaction mixture was stirred for 2 h and quenched with H_2O (200 mL); and the suspension thus obtained was warmed to rt and filtered through celite, rinsing the filter cake with Et_2O (5 × 100 mL). The filtrate was further diluted with Et_2O (500 mL), and the combined organic layers were washed with $Na_2S_2O_3$ (200 mL; sat aq), H_2O (3 × 200 mL), brine (3 × 200 mL) and dried ($MgSO_4$). The solvent was removed under reduced pressure and the residue was purified by flash chromatography (Hexane:EtOAc, 15:1) to give vinyl iodide **21** as an orange oil (19.5 g, 75%, >99:1 *Z*:*E*). **R_f** (Hexane:EtOAc, 15:1) = 0.50; **$[\alpha]_D$** = +12.0 (*c* 1.25, $CHCl_3$); **IR** (neat, cm^{-1}) 1730 (C=O); **^{1}H NMR** δ (500 MHz, $CDCl_3$) 6.33–6.27 (2H, m, *H*C=C*H*), 4.15 (1H, t, *J* = 5.7 Hz, SiOC*H*), 3.69 (3H, s, OCH_3), 2.35–2.33 (2H, m, CH_2), 1.22 (3H, s, CCH_3), 1.17 (3H, s, CCH_3), 0.91 (9H, s, 3SiC(CH_3)$_3$), 0.10 (3H, s, SiCH_3), 0.07 (3H, s, SiCH_3); **^{13}C NMR** δ (125 MHz, $CDCl_3$) 177.3 (C), 138.7 (CH), 83.6 (CH), 75.3 (CH), 51.8 (CH_3), 48.4 (C), 39.4 (CH_2), 25.9 (3CH_3), 22.0 (CH_3), 20.3 (CH_3), 18.2 (C), –3.6 (CH_3), –4.6 (CH_3); ***m/z*** (ESI+, MeOH/DCM) 639 ($[3M+H+K]^{2+}$, 100%), 413 ($[M+H]^+$, 49), 400 (34), 372 (29); **HRMS** (ESI+, MeOH/DCM) $[M+H]^+$ found 413.1010, $C_{15}H_{30}IO_3Si$ requires 413.1003.

(3*S*,5*Z*)-3-(*tert*-Butyldimethylsilanyloxy)-2,2-dimethyl-5-nonen-7-ynoic acid methyl ester 22

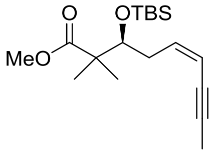

To a suspension of $ZnCl_2$ (23.3 g, 171 mmol) in THF (180 mL) was added 1-propynylmagnesium bromide (244 mL, 142 mmol; 0.5 M in THF) over 15 min and the pale-yellow slurry was stirred for 15 min at 0 °C. Vinyl iodide **21** (19.5 g, 47.4 mmol) in THF (30 mL) and $PdCl_2(PPh_3)_2$ (1.66 g, 2.37 mmol, 5 mol%) rinsed into the flask with THF (10 mL) were added in quick succession, and the pale-yellow mixture was warmed to rt and stirred overnight (~16 h). The solvent was removed under reduced pressure and NH_4Cl (300 mL; sat aq) was added to the resulting brown paste to quench the reaction. The aqueous layer was extracted with DCM (4 × 100 mL), and the combined organic layers were washed with brine (100 mL) and dried ($MgSO_4$). The solvent was removed under reduced pressure to give a brown residue that was purified by flash chromatography (Hexane:DCM, 3:1; dry loaded) to afford enyne **22** as an orange oil (14.2 g, 92%). **R_f** (Hexane:DCM, 3:1) = 0.24; **$[\alpha]_D$** = +2.90 (*c* 1.01, $CHCl_3$); **IR** (neat, cm^{-1}) 2218 (C≡C), 1732 (C=O); **^{1}H NMR** δ (500 MHz, $CDCl_3$) 5.88 (1H, dt, *J* = 10.7, 7.4 Hz, CH_2C*H*=CH), 5.47 (1H, dqt, *J* = 10.7, 2.4, 1.4 Hz, C*H*C≡CCH_3), 4.09 (1H, t, *J* = 5.7 Hz, C*H*OSi), 3.67 (3H, s, OCH_3), 2.55–2.43 (2H, m, CH_2), 2.01 (3H, d, *J* = 2.4 Hz, C≡CCH_3), 1.21 (3H, s, CCH_3), 1.16 (3H, s, CCH_3), 0.90 (9H, s, 3SiC(CH_3)), 0.10 (3H, s, SiCH_3), 0.06 (3H, s, SiCH_3); **^{13}C NMR** δ (125 MHz, $CDCl_3$) 177.5 (C), 139.1 (CH), 110.7 (CH), 90.7 (C), 76.5 (C), 76.2 (CH), 51.6 (CH_3), 48.4 (C), 34.8 (CH_2), 25.9 (3CH_3), 21.9 (CH_3), 20.2 (CH_3), 18.1 (C), 4.4 (CH_3), –3.7 (CH_3), –4.7 (CH_3); ***m/z*** (ESI+, MeOH/DCM) 347 ($[M+Na]^+$, 70%), 325 ($[M+H]^+$, 34), 165 ($[M+2H]^{2+}$, 100); **HRMS** (ESI+, MeOH/DCM) $[M+H]^+$ found 325.2195, $C_{18}H_{33}O_3Si$ requires 325.2193.

(3*S*,5*Z*)-3-Hydroxy-2,2-dimethyl-non-5-en-7-ynoic acid methyl ester 23

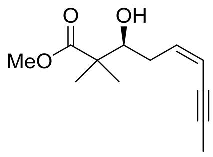

HF (37.7 mL, 1.05 mol; 48% aq) was added to a solution of TBS-ether **22** (11.3 g, 34.9 mmol) in MeCN (70 mL) at 0 °C and the solution was stirred for 15 min, warmed to rt, stirred for 1 h and cooled back down to 0 °C. A suspension of $NaHCO_3$ (84.0 g, 1.00 mol) in $NaHCO_3$ (600 mL; sat aq) was added in small portions over ~15 min [CAUTION: vigorous gas evolution] and the mixture was warmed to rt and stirred for 30 min. The aqueous layer was extracted with DCM (4 × 200 mL) and the combined organic layers were washed with $NaHCO_3$ (200 mL; sat aq), brine (200 mL) and dried ($MgSO_4$). The solvent was removed under reduced pressure and the resulting oil was purified by flash chromatography (Hexane:EtOAc, 3:1) to give β-hydroxyester **23** as a yellow oil (7.28 g, 99%). $\mathbf{R_f}$ (Hexane:EtOAc, 3:1) = 0.44; $\mathbf{[\alpha]_D}$ = –29.7 (*c* 1.01, $CHCl_3$); **IR** (neat, cm^{-1}) 3503 (OH), 2220 (C≡C), 1726 (C=O); $\mathbf{^1H}$ **NMR** δ (400 MHz, $CDCl_3$) 6.00 (1H, dt, *J* = 10.6, 7.3 Hz, CH_2CH=CH), 5.59 (1H, dqt, *J* = 10.6, 2.3, 1.0 Hz, C*H*C≡CH_3), 3.80–3.76 (1H, m, C*H*OH), 3.74 (3H, s, OCH_3), 2.56–2.50 (2H, m, O*H* and CH_AH_B), 2.40–2.31 (1H, m, CH_AH_B), 2.00 (3H, d, *J* = 2.3 Hz, C≡CCH_3), 1.28 (3H, s, CCH_3), 1.27 (3H, s, CCH_3); $\mathbf{^{13}C}$ **NMR** δ (100 MHz, $CDCl_3$) 177.9, (C), 138.8 (CH), 111.7 (CH), 90.7 (C), 76.4 (CH), 76.3 (C), 52.0 (CH_3), 47.1 (C), 32.7 (CH_2), 22.3 (CH_3), 20.3 (CH_3), 4.4 (CH_3); ***m/z*** (EI) 210 ($[M]^+$, 1%), 192 (34), 177 (26), 131 (95), 102 (100); **HRMS** (EI) $[M]^+$ found 210.1259, $C_{12}H_{18}O_3$ requires 210.1256.

(3*S*,5*Z*)-3-Hydroxy-2,2-dimethyl-non-5-en-7-ynoic acid methoxy-methyl amide 24

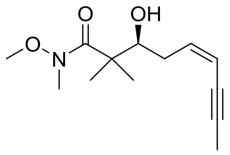

To a suspension of HNMe(OMe)•HCl (13.5 g, 138 mmol) in THF (130 mL) was added ^{n}BuLi (173 mL, 277 mmol; 1.6 M in hexanes) at –78 °C, and the mixture was stirred for 15 min, warmed to rt [CAUTION: butane gas evolution], stirred for 20 min and re-cooled to –78 °C to give a light-yellow solution of the lithium amide of Weinreb's amine. A solution of ester **23** (7.28 g, 34.6 mmol) in THF (50 mL) was added, and the reaction mixture was stirred for 30 min, warmed to rt (~1 h) and stirred for 5 min. NH_4Cl (400 mL; 50% sat aq) was added to quench the reaction, the solution was stirred vigorously until a clean phase separation was visible (~30 min), and the biphasic mixture was separated. The aqueous layer was extracted with Et_2O (5 × 100 mL), and the combined organic layers were washed with H_2O (100 mL), brine (100 mL) and dried ($MgSO_4$). The solvent was removed under reduced pressure and the crude oil thus obtained was purified by flash chromatography (Hexane:EtOAc, 1:1) to give Weinreb amide **24** as an orange-red oil (6.81 g, 82%). $\mathbf{R_f}$ (Hexane:EtOAc, 1:1) = 0.33; $\mathbf{[\alpha]_D}$ = –45.2 (*c* 1.15, $CHCl_3$); **IR** (neat, cm^{-1}) 3453 (OH), 2220 (C≡C), 1624 (C=O); $\mathbf{^1H}$ **NMR** δ (400 MHz, $CDCl_3$) 6.08 (1H, dt, *J* = 10.6, 7.2 Hz, CH_2CH=CH), 5.56 (1H, dtq, *J* = 10.6, 2.1, 1.0 Hz, C*H*C≡CH_3), 3.79 (1H, ddd, *J* = 10.2, 6.7, 2.6 Hz, C*H*OH), 3.73 (3H, s, OCH_3), 3.29 (1H, d, *J* = 6.7 Hz, O*H*), 3.22 (3H, s, NCH_3), 2.61–2.55 (1H, m, CH_AH_B), 2.45–2.37 (1H, m, CH_AH_B), 2.00 (3H, d, *J* = 2.1 Hz, C≡CCH_3), 1.33 (3H, s, CCH_3), 1.32 (3H, s, CCH_3); $\mathbf{^{13}C}$ **NMR** δ (100 MHz, $CDCl_3$) 178.5 (C), 140.1 (CH), 110.8 (CH), 90.3 (C), 77.6 (CH), 76.5 (C), 60.7 (CH_3), 47.2 (C), 33.7 (CH_3), 32.5 (CH_2), 21.5 (CH_3), 20.0 (CH_3), 4.4 (CH_3); ***m/z*** (ESI+, MeOH/DCM) 501 ($[2M+Na]^+$, 41%), 262 ($[M+Na]^+$, 100), 240 ($[M+H]^+$, 34); **HRMS** (ESI+, MeOH/DCM) $[M+Na]^+$ found 262.1407, $C_{13}H_{21}NO_3Na$ requires 262.1414.

(3*S*,5*Z*)-3-(*tert*-Butyldimethylsilanyloxy)-2,2-dimethyl-5-nonen-7-ynoic acid methoxy-methyl-amide 25

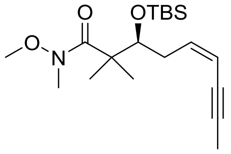

Cautiously, TBSOTf (8.50 mL, 37.0 mmol) was added to a solution of alcohol **24** (6.81 g, 28.5 mmol) and 2,6-lutidine (6.62 mL, 56.9 mmol) in DCM (30 mL) and the reaction mixture was stirred for 1.5 h at –78 °C, warmed to rt (~30 min), and quenched with $NaHCO_3$ (100 mL; sat aq) [CAUTION: gases evolved] and warmed to rt. The aqueous layer was extracted with DCM (3 × 50 mL), and the combined organic layers were washed with brine (100 mL) and dried ($MgSO_4$). The solvent and residual 2,6-lutidine were removed under under reduced pressure (bath temperature = 70 °C), and the crude material was purified by flash chromatography (Hexane:EtOAc, 6:1) to give TBS-ether **25** as an orange oil (9.67 g, 96%). **R_f** (Hexane:EtOAc, 9:1) = 0.15; **$[\alpha]_D$** = +3.30 (*c* 0.54, $CHCl_3$); **IR** (neat, cm^{-1}) 2218 (C≡C), 1651 (C=O); **^{1}H NMR** δ (500 MHz, $CDCl_3$) 5.88 (1H, dt, *J* = 10.6, 7.6 Hz, CH_2CH=CH), 5.46 (1H, br d, *J* = 10.6 Hz, C*H*C≡CCH_3), 4.37 (1H, t, *J* = 5.8 Hz, C*H*OSi), 3.70 (3H, s, OCH_3), 3.17 (3H, s, NCH_3), 2.51–2.41 (2H, m, CH_2), 1.99 (3H, br s, C≡CCH_3), 1.27 (3H, s, CCH_3), 1.23 (3H, s, CCH_3), 0.93 (9H, s, $3SiC(CH_3)_3$), 0.11 (3H, s, $SiCH_3$), 0.08 (3H, s, $SiCH_3$); **^{13}C NMR** δ (125 MHz, $CDCl_3$) 177.6 (C), 139.7 (CH), 110.6 (CH), 90.3 (C), 76.7 (C), 74.3 (CH), 60.4 (CH_3), 49.1 (C), 35.4 (CH_2), 33.9 (CH_3), 26.0 ($3CH_3$), 23.0 (CH_3), 19.4 (CH_3), 18.2 (C), 4.5 (CH_3), –3.6 (CH_3), –4.3 (CH_3); ***m/z*** (ESI+, MeOH/DCM) 729 ($[2M+Na]^+$, 47%), 376 ($[M+Na]^+$, 100), 354 ($[M+H]^+$, 32); **HRMS** (ESI+, MeOH/DCM) $[M+H]^+$ found 354.2459, $C_{19}H_{36}NO_3Si$ requires 354.2459.

(2*E*,6*S*,8*Z*)-6-(*tert*-Butyl-dimethyl-silanyloxy)-5,5-dimethyl-2,8-dodecadien-10-yn-4-one S10

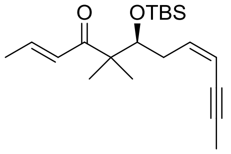

TBS-protected β-hydroxyketone **S10** was prepared according to a modification of the procedure described by Scheidt[8] for the preparation of an analogous enone from a Weinreb amide. A solution of allyl bromide (6.30 mL, 72.8 mmol) in Et_2O (100 mL) was added dropwise to a suspension of magnesium turnings (1.98 g, 81.3 mmol) in Et_2O (46 mL) and the solution was stirred until spontaneous boiling ceased (~30 min), heated to reflux for a further 15 min, cooled to rt and stirred for 1 h to give allylmagnesium bromide (146 mL, 73.0 mmol; 0.50 M in Et_2O) as a grey solution. The Grignard reagent thus obtained was transferred *via* cannula over ~20 min to a solution of Weinreb amide **25** (3.22 g, 9.10 mmol) in Et_2O (20 mL) at –20 °C and the reaction mixture was stirred for 2 h, cooled to –78 °C and quenched by cautious addition (~10 min) of NH_4Cl (100 mL; sat aq). After warming to rt, the aqueous layer was extracted with Et_2O (3 × 100 mL) and the combined organic layers were washed with brine (100 mL) and dried ($MgSO_4$). The solvent was removed under reduced pressure to give the crude allylic ketone **S11** as an orange oil to which triethylamine (25.4 mL, 182 mmol) and DBU (5.44 mL, 36.4 mmol) were added, and the solution was heated to 50 °C overnight (~18 h). The reaction mixture was cooled to rt, diluted with Et_2O (100 mL) and washed with HCl (3 × 50 mL; 1.0 M aq) [CAUTION: exothermic], $NaHCO_3$ (50 mL; sat aq) and brine (50 mL). Drying ($MgSO_4$), removal of the solvent under reduced pressure and purification of the orange oil thus obtained by flash chromatography (Hexane:EtOAc, 10:1) gave the product TBS-protected β-hydroxyketone **S10** as a yellow oil (2.45 g, 81%, 98:2 *E*:*Z*). N.B. Repeated runs typically gave comparable stereoselectivity in the product enone (greater than 96:4 *E*:*Z*). **R_f** (Hexane:EtOAc, 10:1) = 0.53; **$[\alpha]_D$** = –5.90 (*c* 1.01, $CHCl_3$); **IR** (neat, cm^{-1}) 2220 (C≡C), 1687 (C=O), 1626 (C=C); **^{1}H NMR** δ (500 MHz, $CDCl_3$) 6.91 (1H, dq, *J* = 15.1, 6.9 Hz, CH_3CH=CH), 6.58 (1H, dq, *J* = 15.1, 2.2, 1.6 Hz, CH_3CH=C*H*), 5.88 (1H, dt, *J* = 10.7, 7.4 Hz, CH_2CH=CH), 5.44 (1H, br d, *J* = 10.7 Hz, C*H*C≡CCH_3), 4.05 (1H, dd, *J* = 5.8, 5.6 Hz, SiOC*H*), 2.52–2.41 (2H, m, CH_2), 1.99 (3H, d, *J* = 2.2 Hz, C≡CCH_3),

1.90 (3H, dd, *J* = 6.9, 1.6 Hz, C=CHC*H*$_3$), 1.16 (3H, s, CC*H*$_3$), 1.14 (3H, s, CC*H*$_3$), 0.91 (9H, s, 3SiC(C*H*$_3$)$_3$), 0.10 (3H, s, SiC*H*$_3$), 0.06 (3H, s, SiC*H*$_3$); **^{13}C NMR** δ (125 MHz, $CDCl_3$) 203.1 (C), 142.2 (CH), 139.4 (CH), 127.4 (CH), 110.9 (CH), 90.7 (C), 76.5 (CH), 76.4 (C), 52.1 (C), 35.0 (CH_2), 26.0 ($3CH_3$), 21.6 (CH_3), 20.3 (CH_3), 18.2 (CH_3), 18.1 (C), 4.4 (CH_3), –3.7 (CH_3), –4.5 (CH_3); ***m/z*** (ESI+, MeOH/DCM) 357 ($[M+Na]^+$, 100%); **HRMS** (ESI+, MeOH/DCM) $[M+H]^+$ found 335.2395, $C_{20}H_{35}O_2Si$ requires 335.2401.

(6*S*,8*Z*)-6-(*tert*-Butyl-dimethyl-silanyloxy)-5,5-dimethyl-1,8-dodecadien-10-yn-4-one S11

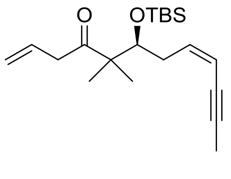

R$_f$ (Hexane:EtOAc, 10:1) = 0.50; **^{1}H NMR** δ (600 MHz, $CDCl_3$) 5.96 (1H, ddt, *J* = 17.1, 10.3, 6.7 Hz, CH_2=C*H*), 5.84 (1H, dt, *J* = 10.6, 7.4 Hz, CH_2C*H*=CH), 5.45 (1H, br d, *J* = 10.6 Hz, C*H*C≡CCH_3), 5.17 (1H, br d, *J* = 10.3, 2.9 Hz, C$H_{cis}H_{trans}$=CH), 5.10 (1H, br d, *J* = 17.1, 2.9 Hz, C$H_{cis}H_{trans}$=CH), 4.06 (1H, t, *J* = 5.7 Hz, C*H*OSi), 3.40 (1H, br dd, *J* = 18.1, 6.8 Hz H_2C=CHC$H_A$$H_B$), 3.29 (1H, br dd, *J* = 18.1, 6.8 Hz, H_2C=CHC$H_A$$H_B$), 2.53–2.39 (2H, m, C*H*$_2$CH=CH), 2.00 (3H, d, *J* = 2.3 Hz, C≡CC*H*$_3$), 1.17 (3H, s, CC*H*$_3$), 1.15 (3H, s, CC*H*$_3$), 0.92 (9H, s, 3SiC(C*H*$_3$)$_3$), 0.11 (3H, s, SiC*H*$_3$), 0.07 (3H, s, SiC*H*$_3$); **^{13}C NMR** δ (150 MHz, $CDCl_3$) 213.0 (C), 139.0 (CH), 131.7 (CH), 117.8 (CH_2), 111.0 (CH), 91.0 (C), 76.5 (CH), 76.4 (C), 53.3 (C), 43.5 (CH_2), 35.1 (CH_2), 26.0 ($3CH_3$), 22.2 (CH_3), 20.2 (C), 18.2 (CH_3), 4.4 (CH_3), –3.8 (CH_3), –4.5 (CH_3).

(2*E*,6*S*,8*Z*)-6-Hydroxy-5,5-dimethyl-2,8-dodecadien-10-yn-4-one 5

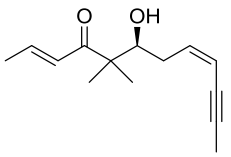

HF (18.3 mL, 510 mmol; 48% aq) was added to a solution of TBS-ether **S10** (5.71 g, 17.0 mmol) in MeCN (40 mL) at 0 °C and the solution was stirred for 15 min, warmed to rt, stirred for 30 min and cooled back down to 0 °C. A suspension of $NaHCO_3$ (50.4 g, 600 mmol) in $NaHCO_3$ (300 mL; sat aq) was added in small portions [CAUTION: vigorous gas evolution] and the mixture was warmed to rt and stirred for 30 min. The aqueous layer was extracted with DCM (4 × 100 mL) and the combined organic layers were washed with $NaHCO_3$ (100 mL; sat aq), brine (100 mL) and dried ($MgSO_4$). The solvent was removed under reduced pressure and the resulting orange oil was purified by flash chromatography (Hexane:EtOAc, 6:1) to give β-hydroxyketone **5** as a light-yellow oil (3.18 g, 85%). **R$_f$** (Hexane:EtOAc, 3:1) = 0.44; **R$_f$** (Hexane:EtOAc, 6:1) = 0.25; **R$_f$** (DCM:Et_2O, 25:1) = 0.63; **$[\alpha]_D$** = –56.1 (*c* 1.05, $CHCl_3$); **IR** (neat, cm^{-1}) 3487 (OH), 2218 (C≡C), 1682 (C=O), 1620 (C=C); **^{1}H NMR** δ (500 MHz, $CDCl_3$) 7.01 (1H, dq, *J* = 15.1, 6.9 Hz, CH_3C*H*=CH), 6.56 (1H, dq, *J* = 15.1, 1.7 Hz, CH=C*H*CO), 6.01 (1H, dt, *J* = 10.6, 7.3 Hz, CH_2C*H*=CH), 5.57 (1H, br dq, *J* = 10.6, 2.2 Hz, C*H*C≡CCH_3), 3.84 (1H, dd, *J* = 10.2, 2.5 Hz, C*H*OH), 2.68 (1H, br s, O*H*), 2.53–2.48 (1H, m, C$H_A$$H_B$), 2.39–2.33 (1H, m, C$H_A$$H_B$), 1.99 (3H, d, *J* = 2.2 Hz, C≡CC*H*$_3$), 1.93 (3H, dd, *J* = 6.9, 1.7 Hz, C=CHC*H*$_3$), 1.23 (3H, s, CC*H*$_3$), 1.20 (3H, s, CC*H*$_3$); **^{13}C NMR** δ (125 MHz, $CDCl_3$) 204.5 (C), 143.9 (CH), 139.3 (CH), 126.4 (CH), 111.5 (CH), 90.6 (C), 76.3 (C), 76.1 (CH), 50.3 (C), 32.5 (CH_2), 21.5 (CH_3), 19.3 (CH_3), 18.4 (CH_3), 4.4 (CH_3); ***m/z*** (ESI+, MeOH/DCM) 463 ($[2M+Na]^+$, 7%), 243 ($[M+Na]^+$, 100), 221 ($[M+H]^+$, 7); **HRMS** (ESI+, MeOH/DCM) $[M+Na]^+$ found 243.1356, $C_{14}H_{20}O_2Na$ requires 243.1356.

Evans-Tishchenko and fragment coupling to give bis-alkynes 3 and 29

(2*E*,4*S*,6*S*,8*Z*)-5,5-dimethyl-6-(3-nitrobenzoyloxy)-2,8-dodecadien-10-yn-4-ol 26

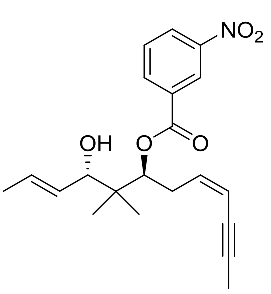

To a solution of β-hydroxyketone **5** (353 mg, 1.60 mmol) and 3-nitrobenzaldehyde (1.36 g, 9.00 mmol) in THF (2 mL) at –20 °C was added freshly prepared samarium diiodide (15.0 mL, 1.50 mmol; 0.1 M in THF) and the solution was stirred for 4 h before quenching of the reaction with sodium potassium tartrate (20 mL; sat aq). The aqueous layer was extracted with DCM (3 × 20 mL) and the combined organic layers were washed with a mixture of water (10 mL) and $Na_2S_2O_3$ (10 mL; sat aq), brine (20 mL) and dried ($MgSO_4$). The solvent was removed under reduced pressure and the brown residue thus obtained was dissolved in EtOH (20 mL). $NaHSO_3$ (4.00 mL; 4.5 M aq) was added, and the mixture was stirred for 3 h. The precipitate was filtered, washed with Et_2O (5 × 10 mL) and the solvent was removed under reduced pressure from the filtrate. The resulting oil was dissolved in Et_2O (50 mL), and the organic layer was washed with water (10 mL), brine (10 mL) and dried ($MgSO_4$). The solvent was removed under reduced pressure and the yellow residual oil thus obtained was purified by flash chromatography (Hexane:EtOAc, 6:1) to give 1,3-*anti* diol monoester **26** as a yellow syrup (558 mg, 94%). $\mathbf{R_f}$ (Hexane:DCM, 20:1) = 0.27; $\mathbf{R_f}$ (Hexane:EtOAc, 6:1) = 0.17; $[\alpha]_D$ = +44.0 (*c* 0.50, $CHCl_3$); **IR** (neat, cm^{-1}) 3528 (OH), 2218 (C≡C), 1721 (C=O), 1690 (C=O), 1616 (C=C), 1531 (NO); **^{1}H NMR** δ (500 MHz, $CDCl_3$) 8.90 (1H, appt t, *J* = 1.8 Hz, Ar*H*), 8.46 (1H, ddd, *J* = 8.2, 2.2, 1.1 Hz, Ar*H*), 8.41 (1H, ddd, *J* = 8.2, 1.3, 1.1 Hz, Ar*H*), 7.69 (1H, appt t, *J* = 8.0 Hz, Ar*H*), 5.81 (1H, ddd, *J* = 10.6, 8.9, 6.3 Hz, CH_2CH=CH), 5.70 (1H, dq, *J* = 15.3, 6.3 Hz CH_3CH=CH), 5.60 (1H, ddq, *J* = 15.3, 7.3, 1.3 Hz, CH_3CH=C*H*), 5.46 (1H, br d, *J* = 10.6 Hz, C*H*C≡CCH_3), 5.42 (1H, dd, *J* = 10.6, 2.7 Hz, C*H*OCOAr), 3.86 (1H, d, *J* = 7.3 Hz, C*H*OH), 2.93-2.87 (1H, m, CH_AH_B), 2.66-2.61 (1H, m, CH_AH_B), 2.34 (1H, br s, O*H*), 2.04 (3H, d, *J* = 2.3 Hz, C≡CCH_3), 1.73 (3H, dd, *J* = 6.3, 1.3 Hz, CH_3CH=CH), 1.05 (3H, s, $OCCCH_3$), 1.02 (3H, s, $OCCCH_3$); **^{13}C NMR** δ (125 MHz, $CDCl_3$) 164.9 (C), 148.3 (C), 137.2 (CH), 135.6 (CH), 131.9 (C) 129.6 (CH), 129.5 (CH), 129.4 (CH), 127.5 (CH), 124.8 (CH), 112.8 (CH), 91.2 (C), 78.8 (CH), 76.3 (CH), 76.1 (C), 41.9 (C), 30.6 (CH_2), 18.9 (CH_3), 18.5 (CH_3), 17.9 (CH_3), 4.3 (CH_3); ***m/z*** (EI) 371 ($[M]^+$, 1%), 257 (6), 177 (14), 150 (100), 134 (26), 133 (14), 119 (86); **HRMS** (EI) $[M]^+$ found 371.1738, $C_{21}H_{25}NO_5$ requires 371.1733.

(2*E*,4*S*,6*S*,8*Z*)-4-(4-methoxybenzyloxy)-5,5-dimethyl-6-(3-nitrobenzoyloxy)-2,8-dodecadien-10-yne S12

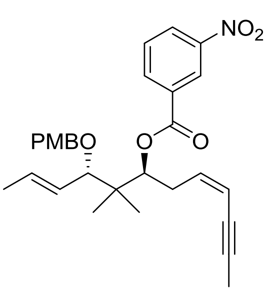

PMB-protected 1,3-*anti* diol monoester **S12** was prepared according to a modification of the *O*-PMB-protection procedure described by Rai and Basu.[9] To a solution of 1,3-*anti* diol monoester **26** (186 mg, 0.50 mmol) and 4-methoxybenzyl-2,2,2-trichloroacetimidate (424 mg, 1.50 mmol) in toluene (25 mL) was added scandium(III) trifluoromethanesulfonate (24.6 mg, 0.050 mmol) at 0 °C and the reaction mixture was warmed to rt and stirred for 1 h, after which time a yellowing of the solution had occurred. Water (50 mL) was added to quench the reaction, and the aqueous layer was extracted with DCM (3 × 20 mL). The combined organic layers were washed with brine (20 mL) and dried ($MgSO_4$). Removal of the solvent under reduced pressure gave a yellow residue which was purified by flash chromatography (Hexane:EtOAc, 6:1) to give an inseparable mixture (~1.8:1) of PMB-protected 1,3-*anti* diol monoester **S12** and PMB-dimer **S13** (242 mg, 0.39 mmol **S12**, 79% by ^{1}H NMR) as a yellow syrup which was used as a 0.05 M solution in methanol for subsequent reactions. N.B.: Ratios of

product **S12** to PMB_2O **S13** obtained after purification varied from 6.7:1 to 1.8:1. **R_f** (Hexane:EtOAc, 6:1) = 0.40; **[α]$_D$** = +82.0 (*c* 1.00, $CHCl_3$); **IR** (neat, cm^{-1}) 2216 (C≡C), 1722 (C=O), 1612 (C=C), 1585 (C=C), 1533 (C=C), 1512 (C=C); **^{1}H NMR** δ (500 MHz, $CDCl_3$) 8.79 (1H, ddd, *J* = 2.4, 1.5, 0.3 Hz, NO_2Ar*H*), 8.39 (1H, ddd, *J* = 8.2, 2.4, 1.2 Hz, NO_2Ar*H*), 8.29 (1H, ddd, *J* = 7.7, 1.5, 1.2 Hz, NO_2Ar*H*), 7.56 (1H, ddd, *J* = 8.2, 7.7, 0.3 Hz, NO_2Ar*H*), 7.22-7.19 (2H, m, 2CH_3OAr*H*), 6.78-6.75 (2H, m, 2CH_3OAr*H*), 5.84 (1H, ddd, *J* = 10.6, 8.4, 6.8 Hz, CH_2C*H*=CH), 5.69 (1H, dq, *J* = 15.4, 6.5 Hz, CH_3C*H*=CH), 5.51 (1H, ddq, *J* = 15.4, 8.7, 1.7 Hz, CH_3CH=C*H*), 5.47 (1H, dd, *J* = 9.9, 3.2 Hz, C*H*OCOAr), 5.42 (1H, dqt, *J* = 10.6, 2.2, 1.2 Hz, C*H*C≡CCH_3), 4.38 (1H, d, *J* = 11.0 Hz, C$H_A$$H_B$Ar), 4.07 (1H, d, *J* = 11.0 Hz, CH$_A$$H_B$Ar), 3.78 (3H, s, OC$H_3$), 3.52 (1H, d, *J* = 8.7 Hz, C*H*OCH_2Ar), 2.84-2.77 (1H, m, C$H_C$$H_D$CH=CH), 2.66-2.61 (1H, m, CH$_C$$H_D$CH=CH), 1.98 (3H, d, *J* = 2.2 Hz, C≡CCH_3), 1.80 (3H, dd, *J* = 6.5, 1.7 Hz, CH_3CH=CH), 1.07 (3H, s, CCH_3), 1.04 (3H, s, CCH_3); **^{13}C NMR** δ (125 MHz, $CDCl_3$) 163.9 (C), 158.8 (C), 148.1 (C), 137.9 (CH), 135.4 (CH), 132.6 (C), 131.4 (CH), 130.7 (C), 129.6 (2CH), 129.3 (CH), 127.9 (CH), 127.0 (CH), 124.6 (CH), 113.4 (2CH), 112.0 (CH), 90.7 (C), 84.4 (CH), 77.8 (CH), 76.2 (C), 69.7 (CH_2), 55.1 (CH_3), 41.8 (C), 31.0 (CH_2), 19.8 (CH_3), 19.7 (CH_3), 17.9 (CH_3), 4.3 (CH_3); ***m/z*** (EI) 491 ([M]$^+$, 10%), 348 (75), 332 (47), 255 (67), 228 (55), 227 (63), 211 (39), 197 (50), 151 (100); **HRMS** (EI) [M]$^+$ found 491.2306, $C_{29}H_{33}NO_6$ requires 491.2308.

***p*-Methoxy{[(*p*-methoxyphenyl)methoxy]methyl}benzene (PMB_2O) S13**

R_f (Hexane:EtOAc, 6:1) = 0.40; **^{1}H NMR** δ (500 MHz, $CDCl_3$) 7.32-7.30 (4H, m, 4Ar*H*), 6.93-6.90 (4H, m, 4Ar*H*), 4.49 (4H, s, 2CH_2), 3.84 (6H, s, 2OCH_3); **^{13}C NMR** δ (125 MHz, $CDCl_3$) 159.2 (2C), 130.5 (2C), 129.4 (4CH), 113.8 (4CH), 71.5 (2CH_2), 55.3 (2CH_3).

(2*E*,4*S*,6*S*,8*Z*)-4-(4-methoxybenzyloxy)-5,5-dimethyl-2,8-dodecadien-10-yn-6-ol 27

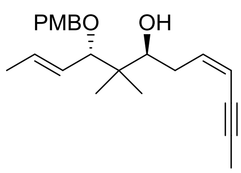

A solution of PMB-protected 1,3-*anti* diol monoester **S12** (6.80 mL, 0.39 mmol; 0.05 M in MeOH), LiOH (196 mg, 4.68 mmol) and water (0.68 mL) was heated to reflux overnight (~18 h) and cooled to rt. The reaction mixture was concentrated under reduced pressure and partitioned between Et_2O (20 mL) and NaOH (20 mL; 2.0 M aq) and the aqueous layer was extracted with Et_2O (3 × 10 mL). The combined organic layers were washed with NaOH (3 × 10 mL; 3.0 M aq), water (10 mL), brine (10 mL) and dried ($MgSO_4$). The solvent was removed under reduced pressure to give yellow oil which was purified by flash chromatography (Hexane:EtOAc, 6:1) to give an inseparable mixture (~2:1) of alcohol **27** contaminated with PMB-dimer **S13** as a light-yellow oil (167.8 mg, 0.35 mmol **27**, 91% by ^{1}H NMR) which was used as a 0.05 M solution in toluene in subsequent reactions without further purification. N.B.: Ratios of product **27** to PMB_2O **S13** obtained after purification varied from 7.4:1 to 2:1. **R_f** (Hexane:EtOAc, 6:1) = 0.40; **[α]$_D$** = –6.00 (*c* 0.50, $CHCl_3$); **IR** (neat, cm^{-1}) 3487 (OH), 1610 (C=C), 1585 (C=C), 1510 (C=C); **^{1}H NMR** δ (500 MHz, $CDCl_3$) 7.26-7.23 (2H, m 2Ar*H*), 6.90-6.87 (2H, m, 2Ar*H*), 6.07 (1H, dt, *J* = 10.7, 7.2 Hz, CH_2C*H*=CH), 5.71 (1H, dq, *J* = 15.4, 6.4 Hz, CH_3C*H*=CH), 5.54-5.49 (2H, m, CH_3CH=C*H* and C*H*C≡CCH_3), 4.54 (1H, d, *J* = 11.2 Hz, C$H_A$$H_B$Ar), 4.22 (1H, d, *J* = 11.2 Hz, CH$_A$$H_B$Ar), 3.82 (3H, s, OC$H_3$), 3.74 (1H, br s, OH), 3.68 (1H, d, *J* = 8.6 Hz, C*H*OCH_2Ar), 3.61 (1H, dd, *J* = 10.1, 2.1 Hz, C*H*OH), 2.57-2.52 (1H, m, C$H_C$$H_D$CH=CH), 2.31-2.25 (1H, m, CH$_C$$H_D$CH=CH), 1.98 (3H, d, *J* = 2.3 Hz, C≡CCH_3), 1.82 (3H, dd, *J* = 6.4, 1.6 Hz, CH_3CH=CH), 0.94 (3H, s, CCH_3), 0.92 (3H, s, CCH_3); **^{13}C NMR** δ (125 MHz, $CDCl_3$) 159.2 (C), 140.8 (CH), 131.4 (CH), 130.1 (C), 129.5 (2CH), 127.6 (CH), 113.8 (2CH), 110.2 (CH), 90.0 (C), 87.8 (CH), 76.7 (C), 69.9 (CH_2), 55.3 (CH_3), 40.9 (C), 32.8 (CH_2), 21.7 (CH_3), 21.0 (CH_3), 17.9 (CH_3), 4.4 (CH_3); ***m/z*** (ESI+, MeOH) 707 ([2M+Na]$^+$, 100%), 685 ([2M+H]$^+$, 7), 381 ([M+K]$^+$, 14), 365 ([M+Na]$^+$, 89), 343 ([M+H]$^+$, 4); **HRMS** (ESI+, MeOH) [M+H]$^+$ found 343.2272, $C_{22}H_{31}O_3$ requires 343.2268.

* one CH obscured by $CDCl_3$

2-[(2'*R*,3'*E*)-2'-Methoxy-hept-3'-en-5'-yn-1'-yl]-oxazole-4-carboxylic acid (1*S*,3*Z*)-1-[(2'*S*,3'*E*)-2'-(4-methoxy-benzyloxy)-1',1'-dimethylpent-3'-en-1'-yl]-3-hepten-5-yn-1-yl ester 3

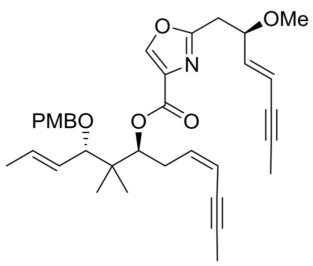

Two stock solutions, the first [Solution 1] containing acid **28** (11.8 mg, 0.05 mmol) in toluene (2 mL; ultrasound assisted dissolution); and the second [Solution 2] containing 2,4,6-trichlorobenzoyl chloride (48.6 mg, 0.20 mmol) and triethylamine (0.03 mL, 0.29 mmol) in toluene (3 mL) were prepared for the purpose of generating portionwise an activated ester. Solution 2 (0.6 mL) was added to Solution 1 (0.4 mL) and the mixture was stirred for 30 min at rt, before addition of the resulting activated ester solution to a heated (40 °C) solution of alcohol **27** (1.00 mL, 0.05 mmol; 0.05 M in toluene) and 4-dimethylaminopyridine (19.0 mg, 0.16 mmol) and stirring of the pale yellow reaction mixture for 30 min. This procedure was repeated until all activated ester solutions were added to alcohol **27** (5 additions, 2.5 h) and stirring was continued overnight (~18 h). The reaction mixture was cooled to rt, diluted with DCM (30 mL), poured into NH_4Cl (10 mL; sat aq) and the aqueous layer was extracted with DCM (3 × 10 mL). The combined organic layers were washed with HCl (10 mL, 1.0 M aq), $NaHCO_3$ (10 mL; sat aq), brine (10 mL), dried ($MgSO_4$) and the solvent was removed under reduced pressure. Flash chromatography with gradient elution (Hexane:EtOAc, 6:1 → 3:1, dry loaded) of the resulting brown residue gave oxazole ester bis-alkyne **3** as a yellow syrup (20.0 mg, 71%).

Large-scale preparation: The above procedure was repeated commencing from acid **28** (106 mg, 0.45 mmol) in toluene (10 mL) [Solution 1]; 2,4,6-trichlorobenzoyl chloride (444 mg, 1.80 mmol) and triethylamine (0.36 mL, 2.57 mmol) in toluene (10 mL) [Solution 2]; and alcohol **27** (4.50 mL, 0.45 mmol; 0.1 M in toluene) and 4-dimethylaminopyridine (171 mg, 3.11 mmol) in toluene (0.5 mL); with Solution 3 generated portionwise from proportionate volumes of Solution 1 and Solution 2 [2 mL of each *per* Solution 3 preparation] and added to the alcohol **27** solution with a toluene rinse (0.2 mL) on each of the 5 additions. Quenching with NH_4Cl (10 mL, sat aq) and subsequent concentration of the reaction mixture under reduced pressure; workup with DCM (3 × 20 mL), HCl (25 mL, 0.2 M aq), $NaHCO_3$ (10 mL, sat aq), H_2O (10 mL) and brine (10 mL); and drying ($MgSO_4$) followed by removal of the solvent under reduced pressure and submission of the residue thus obtained to the same conditions of flash chromatography gave oxazole ester bis-alkyne **3** as a yellow syrup (158 mg, 63%). **R_f** (Hexane:EtOAc, 3:1) = 0.51; **$[\alpha]_D$** = +30.2 (*c* 1.09, $CHCl_3$); **IR** (neat, cm^{-1}) 2224 (C≡C), 1738 (C=O), 1717 (C=N), 1667 (C=C), 1612 (C=C), 1584 (C=C), 1514 (C=C); **^{1}H NMR** δ (600 MHz, $CDCl_3$) 8.00 (1H, s, NC=C*H*), 7.28 (2H, d, *J* = 8.6 Hz, 2ArC*H*), 6.85 (2H, d, *J* = 8.6 Hz, 2ArC*H*), 5.94 (1H, dd, *J* = 15.9, 7.7 Hz, $CH(OCH_3)CH$=CH), 5.85 (1H, dt, *J* = 10.3, 7.3 Hz, CH_2CH=CH), 5.71 (1H, br dq, *J* = 15.9, 2.1 Hz, $CH(OCH_3)CH=CH$), 5.67 (1H, dq, *J* = 15.3, 6.4 Hz, CH_3CH=CH), 5.46 (1H, ddq, *J* = 15.3, 8.6, 1.3 Hz, $CH_3C=CH$), 5.44 (1H, br dq, *J* = 10.3, 2.1 Hz, $CH_2CH=CH$), 5.40 (1H, dd, *J* = 9.5, 3.5 Hz, C*H*OCOAr), 4.39 (1H, d, *J* = 10.8 Hz, CH_AH_BAr), 4.17 (1H, ddd, *J* = 7.7, 7.3, 5.6 Hz, $CHOCH_3$), 4.12 (1H, d, *J* = 10.8 Hz, CH_AH_BAr), 3.81 (3H, s, $ArOCH_3$), 3.51 (1H, d, *J* = 8.6 Hz, $CHOCH_2Ar$), 3.29 (3H, s, $CHOCH_3$), 3.10 (1H, dd, *J* = 15.1, 7.7 Hz, $CH_CH_DCHOCH_3$), 2.99 (1H, dd, *J* = 15.1, 5.6 Hz, $CH_CH_DCHOCH_3$), 2.71-2.62 (2H, m, CH_2CH=CH), 1.98 (3H, d, *J* = 2.1 Hz, $CH_2CH=CHC≡CCH_3$), 1.96 (3H, d, *J* = 2.1 Hz, $CH(OCH_3)CH=CHC≡CCH_3$), 1.78 (3H, dd, *J* = 6.4, 1.3 Hz, CH_3CH), 1.00 (3H, s, CCH_3), 0.97 (3H, s, CCH_3); **^{13}C NMR** δ (150 MHz, $CDCl_3$) 162.3 (C), 160.7 (C), 158.9 (C), 143.5 (CH), 139.8 (CH), 138.3 (CH), 133.6 (C), 131.1 (C), 131.0 (CH), 129.5 (2CH), 127.9 (CH), 114.0 (CH), 113.5 (2CH), 111.5 (CH), 90.4 (C), 87.7 (C), 84.1 (CH), 79.2 (CH),* 76.5 (C), 69.8 (CH_2), 56.7 (CH_3), 55.2 (CH_3), 41.8 (C), 34.6 (CH_2), 30.9 (CH_2), 19.5

(CH_3), 19.4 (CH_3), 17.9 (CH_3), 4.3 (CH_3), 4.2 (CH_3); ***m/z*** (ESI+, MeOH) 1142 ($[2M+Na]^+$, 62%), 598 ($[M+K]^+$, 7), 582 ($[M+Na]^+$, 100), 560 ($[M+H]^+$, 17); **HRMS** (ESI+, MeOH) $[M+Na]^+$ found 582.2802, $C_{34}H_{41}NO_6Na$ requires 582.2826.
* one alkyne C and one CH obscured by $CDCl_3$

2-[(2’*R*,3’*E*)-2’-Methoxy-hept-3’-en-5’-yn-1’-yl]-oxazole-4-carboxylic acid (1*S*,3*Z*)-1-[(2’*S*,3’*E*) - 1’,1’-dimethyl-2’-hydroxypent-3’-en-1’-yl]-3-hepten-5-yn-1-yl ester 29

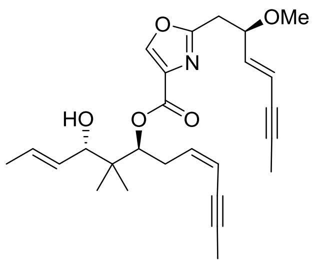

To a stirred biphasic solution of **3** (14.0 mg, 25 µmol) in DCM (1.5 mL) and aqueous phosphate buffer (1.5 mL, 0.5 M in $NaHPO_4$ and NaH_2PO_4) was added DDQ (25.0 mg, 110.0 µmol). The solution was stirred for 25 minutes then $NaHCO_3$ (1.5 mL, sat aq) and $Na_2S_2O_3$ (1.5 mL, sat aq) were added. The solution was diluted with H_2O (5 mL) and extracted with EtOAc (3 × 10 mL) and the organic phase washed with brine (10 mL, sat. aq.), dried (Na_2SO_4) and solvent removed under reduced pressure. Reverse phase HPLC (isocratic 30:70 H_2O:MeCN 0.1% TFA) gave the deprotected bis-alkyne **29** as a slightly yellow oil (10.2 mg, 93%). $\mathbf{R_f}$ (Hexane:EtOAc 3:1) = 0.20; $\mathbf{R_t}$ (H_2O:MeCN 30:70 0.1% TFA) = 10 min; **^{1}H NMR** δ (600 MHz, $CDCl_3$) 8.15 (1H, s, NC=C*H*), 5.91 (1H, dd, *J* = 15.9, 7.7 Hz, CH(OCH_3)C*H*=CH), 5.80 (1H, dt, *J* = 10.6, 7.3 Hz, CH_2C*H*=CH), 5.70 (1H, br dq, *J* = 15.9, 2.1 Hz, CH(OCH_3)CH=C*H*), 5.68 (1H, dq, *J* = 15.3, 5.8 Hz, CH_3C*H*=CH), 5.53 (1H, ddq, *J* = 15.3, 7.3, 1.3 Hz, CH_3HC=C*H*), 5.46 (1H, br dq, *J* = 10.6, 2.2 Hz, CH_2CH=C*H*), 5.26 (1H, dd, *J* = 9.9, 3.2 Hz, C*H*OCOAr), 4.13 (1H, br td, *J* = 7.7, 5.6 Hz, C*H*OCH_3), 3.88 (1H, d, *J* = 7.3, C*H*OH), 3.26 (3H, s, CHOCH_3), 3.10 (1H, dd, *J* = 15.1, 7.7 Hz, CH_AH_BCHOCH_3), 3.00 (1H, dd, *J* = 15.1, 5.6 Hz, CH_AH_BCHOCH_3), 2.76-2.68 (1H, m, CH_CH_DCH=CH), 2.68-2.61 (1H, m, CH_CH_DCH=CH), 1.99 (3H, d, *J* = 2.2 Hz, CH_2CH=CHC≡CCH_3), 1.94 (3H, d, *J* = 2.1 Hz, CH(OCH_3)CH=CHC≡CCH_3), 1.70 (3H, dd, *J* = 6.4, 1.3 Hz, CH_3CH), 0.98 (3H, s, CCH_3), 0.94 (3H, s, CCH_3); **^{13}C NMR** δ (126 MHz, $CDCl_3$) 163.0 (C), 161.5 (C), 144.3 (CH), 139.6 (CH), 137.7 (CH), 133.1 (C), 129.5 (CH), 129.3 (CH), 114.4 (CH), 112.4 (CH), 91.1 (C), 88.0 (C), 79.2 (CH), 79.0 (CH),* 76.6 (C), 76.4 (CH), 56.8 (CH_3), 42.0 (C), 34.7 (CH_2), 30.6 (CH_2), 19.4 (CH_3), 18.9 (CH_3), 18.0 (CH_3), 4.5 (CH_3), 4.4 (CH_3); ***m/z*** (ESI+, MeCN) 901 ($[2M+Na]^+$, 21.8%), 478 ($[M+K]^+$, 10.5), 462 ($[M+Na]^+$, 100) 440 ($[M+H]^+$, 0.57); **HRMS** (ESI+, MeCN) $[M+Na]^+$ found 462.2257; $C_{26}H_{33}NO_5Na$ requires 462.2251.
* one alkyne C obscured by $CDCl_3$

Self-assembly metathesis reaction to give di-lactones 2 and 34

(16,16')-Bis(4-methoxybenzyloxy)-(9,10,9',10')-tetradehydrido-disorazole C_1 2

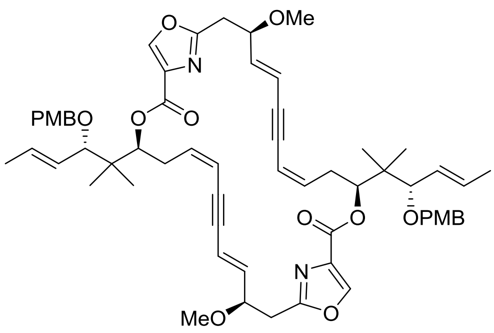

In a glove box, **3** (11.2 mg, 20 µmol) was dissolved in toluene (0.5 mL) and was stirred with a mixture (1:1) of 4 and 5 Å activated powdered molecular sieves (20 mg) for 20 minutes. Catalyst **30** (4.16 mg, 4.0 µmol) was then added, the reaction flask sealed, removed from the glovebox and stirred for 16 h at rt. The solution was filtered through a silica plug, washed with EtOAc (10 mL) and concentrated *in vacuo*. Purification by RP HPLC (isocratic 15:85 H_2O:MeCN 0.1% TFA) gave products **2** (5.1 mg, 51 %) and **34** (1.1 mg, 11 %). $\mathbf{R_f}$ (Hexane:EtOAc 1:1) = 0.50; $\mathbf{R_t}$ (15:85 H_2O:MeCN 0.1% TFA) = 25.7 min; $[\alpha]_D$ = +125.0 (*c* 0.80, $CHCl_3$); **IR** (neat, cm^{-1}) 1734 (C=O), 1718 (C=N), 1612 (C=C), 1584 (C=C) 1513 (C=C); ^{1}**H NMR** δ (500 MHz, $CDCl_3$) 7.97 (2H, s, NC=C*H*), 7.26 (4H, d, *J* = 8.6 Hz, 2ArC*H*) 6.83 (4H, d, *J* = 8.6 Hz, 2ArC*H*), 5.96 (2H, dd, *J* = 15.8, 7.7 Hz, CH(OCH_3)C*H*=CH), 5.98-5.92 (2H, m, CH_2C*H*=CH), 5.65 (2H, br dq, *J* = 15.3, 6.5 Hz, CH_3C*H*=CH), 5.63 (2H, br dd, *J* = 15.8, 1.9 Hz, CH(OCH_3)CH=C*H*), 5.50 (2H, br d, *J* = 10.0 Hz, CH_2CH=C*H*), 5.49 (2H, dd, *J* = 10.9, 3.0 Hz, C*H*OCOAr), 5.45 (2H, ddq, *J* = 15.3, 8.6, 1.4 Hz, CH_3HC=C*H*), 4.37 (2H, d, *J* = 10.7 Hz, C$H_A$$H_B$Ar), 4.16-4.12 (2H, m, C*H*OCH_3), 4.10 (2H, d, *J* = 10.7 Hz, C$H_A$$H_B$Ar), 3.79 (6H, s, ArOC$H_3$), 3.46 (2H, d, *J* = 8.6 Hz, C*H*OCH_2Ar), 3.35 (6H, s, CHOCH_3), 3.31 (2H, dd, *J* = 14.2, 4.1 Hz, C$H_C$$H_DCHOCH_3$), 3.01 (2H, dd, *J* = 14.2, 9.6 Hz, C$H_C$$H_DCHOCH_3$), 2.94 (2H, dt, *J* = 13.5, 10.9 Hz, C$H_E$$H_F$CH=CH), 2.36 (2H, br d, *J* = 13.5 Hz, C$H_E$$H_F$CH=CH), 1.77 (6H, dd, *J* = 6.5, 1.4 Hz, CH_3CH=CH), 0.98 (12H, s, CCH_3); ^{13}**C NMR** δ (126 MHz, $CDCl_3$) δ 161.9 (C), 160.6 (C), 159.0 (C), 143.5 (CH), 141.4 (CH), 140.6 (CH), 133.7 (C), 131.3 (CH), 131.1 (C), 129.8 (2CH), 128.0 (CH), 113.8 (CH), 113.7 (2CH), 112.1 (CH), 90.9 (C), 88.0 (C), 84.2 (CH), 79.8 (CH), 76.4 (CH), 70.0 (CH_2), 57.0 (CH_3), 55.4 (CH_3), 41.8 (C), 34.5 (CH_2), 31.5 (CH_2), 19.5 (CH_3), 19.4 (CH_3), 18.0 (CH_3); ***m/z*** (ESI+, MeCN) 1049 ($[M+K]^+$, 2%), 1033 ($[M+Na]^+$, 100), 1011 ($[M+H]^+$, 5); **HRMS** (ESI+, MeCN) $[M+Na]^+$ found 1033.4862, $C_{60}H_{70}N_2O_{12}Na$ requires 1033.4827. ^{1}H and ^{13}C NMR spectroscopic data in good agreement with the literature.[10]

Head-to-head coupled analogue of (16,16')-bis(4-methoxybenzyloxy)-(9,10,9',10')-tetradehydrido-disorazole C_1 34

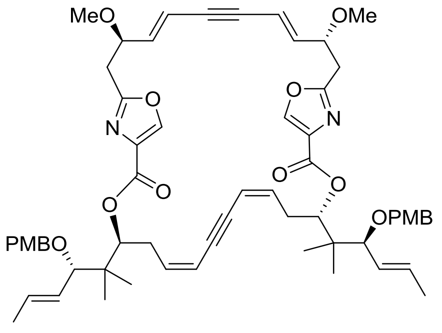

$\mathbf{R_f}$ (Hexane:EtOAc 2:1) = 0.13; $\mathbf{R_t}$ (15:85 H_2O:MeCN 0.1% TFA) = 40 min; $[\alpha]_D$ = 181.8 (*c* 0.22, $CHCl_3$); **IR** (neat, cm^{-1}) 1735 (C=O), 1718 (C=N), 1612 (C=C), 1584 (C=C), 1514 (C=C); ^{1}**H NMR** δ (600 MHz, $CDCl_3$) 7.83 (2H, s, NC=C*H*), 7.25 (4H, m, 2ArC*H* (doublet obscured by solvent peak)) 6.81 (4H, d, *J* = 8.7 Hz, 2ArC*H*), 5.92 (2H, td, *J* = 10.2, 5.2 Hz, CH_2C*H*=CH), 5.88 (2H, dd, *J* = 15.3, 7.9 Hz, CH(OCH_3)C*H*=CH) 5.63 (2H, dq, *J* = 15.3, 6.5 Hz, CH_3C*H*=CH), 5.61 (2H, br d, *J* = 15.3 Hz, CH(OCH_3)CH=C*H*), 5.51 (2H, d, *J* = 10.2 Hz, CH_2CH=C*H*), 5.48 (2H, dd, *J* = 10.7, 2.7 Hz, C*H*OCOAr) 5.43 (2H, ddd, *J* = 15.3, 8.6, 1.5 Hz, CH_3CH=C*H*), 4.34 (2H, d, *J* = 10.6 Hz, C$H_A$$H_B$Ar), 4.12 (2H, ddd, *J* = 10.4, 7.9, 4.2 Hz, C*H*OCH_3), 4.08 (2H, d, *J* = 10.6 Hz, C$H_A$$H_B$Ar), 3.78 (6H, s, ArOC$H_3$), 3.46 (2H, d, *J* = 8.6 Hz, C*H*OCH_2Ar), 3.32 (6H, s, CHOCH_3), 3.25 (2H, dd, *J* = 14.2, 4.2

Hz, $CH_CH_DCHOCH_3$), 2.93 (2H, dd, J = 14.2, 10.4 Hz, $CH_CH_DCHOCH_3$), 2.91-2.84 (2H, m, CH_EH_FCH=CH), 2.41 (2H, br d, J = 14.2 Hz, CH_EH_FCH=CH), 1.76 (6H, dd, J = 6.5, 1.5 Hz, CH_3CH=CH), 0.96 (6H, s, CCH_3), 0.93 (6H, s, CCH_3); **^{13}C NMR** δ (126 MHz, $CDCl_3$) δ 161.4 (C), 160.7 (C), 159.0 (C), 143.7 (CH), 141.4 (CH), 139.3 (CH), 133.6 (C), 131.2 (CH), 131.0 (C), 129.9 (2CH), 128.0 (CH), 113.7 (CH), 113.6 (2CH), 112.3 (CH), 90.8 (C), 87.9 (C), 84.0 (CH), 80.0 (CH), 76.5 (CH), 70.1 (CH_2), 56.9 (CH_3), 55.6 (CH_3), 41.6 (C), 34.1 (CH_2), 31.7 (CH_2), 19.5 (CH_3), 19.3 (CH_3), 18.1 (CH_3); ***m/z*** (ESI+, MeCN)1049 ($[M+K]^+$, 3%) 1033 ($[M+Na]^+$, 100), 1011 ($[M+H]^+$, 8); **HRMS** (ESI+, MeCN) $[M+Na]^+$ found 1033.4856, $C_{60}H_{70}N_2O_{12}Na$ requires 1033.4827.

(9,10,9',10')-Tetradehydrido-disorazole C_1 35

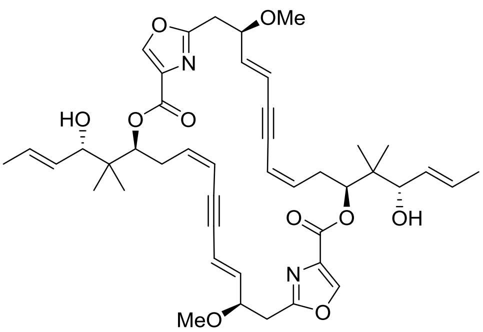

To a biphasic mixture of **2** (4.0 mg, 4.5 μmol), CH_2Cl_2 (0.5 mL) and aqueous phosphate buffer (0.5 mL, 0.5 M in NaH_2PO_4 and Na_2HPO_4) was added DDQ (9 mg, 40 μmol). After 15 min the reaction mixture was quenched with $NaHCO_3$ (2 mL sat aq) and $Na_2S_2O_4$ (2 mL, sat aq) and extracted with ethyl acetate. The organic phase was washed with brine, dried over Na_2SO_4 and concentrated *in vacuo*. Purification by RP HPLC (isocratic 40:60 H_2O:MeCN 0.1% TFA) gave product **35** (2.1 mg, 61 %). **R_f** (Hexane:EtOAc 1:1) = 0.28; **R_t** (40:60 H_2O:MeCN 0.1% TFA) = 15.6 min; **$[\alpha]_D$** = 175.0 (*c* 0.40, $CHCl_3$); **^{1}H NMR** δ (500 MHz, $CDCl_3$) 8.11 (2H, s, NC=C*H*), 6.04 (2H, dd, J = 15.9, 7.3 Hz, $CH(OCH_3)CH$=CH), 5.94 (2H, td, J = 10.5, 5.0 Hz, CH_2CH=CH), 5.70 (2H, br d, J = 15.9 Hz, $CH(OCH_3)CH$=C*H*), 5.68 (2H, dq, J = 15.1, 5.9 Hz, CH_3CH=CH), 5.58 (2H, br d, J = 10.5 Hz, CH_2CH=C*H*), 5.54 (2H, dd, J = 15.1, 6.9 Hz, CH_3CH=C*H*), 5.35 (2H, br d, J = 10.2 Hz, C*H*OCOAr), 4.12 (2H, br s, $CHOCH_3$), 3.87 (2H, d, J = 6.9 Hz, C*H*OH), 3.36 (6H, s, $CHOCH_3$), 3.29 (2H, dd, J = 14.1, 3.3 Hz, $CH_CH_DCHOCH_3$), 3.09-2.95 (4H, m, $CH_CH_DCHOCH_3$ and CH_EH_FCH=CH), 2.41 (2H, br d, J = 12.4 Hz, CH_EH_FCH=CH), 1.71 (6H, d, J = 5.9, CH_3CH), 0.98 (6H, s, CCH_3), 0.96 (6H, s, CCH_3); **^{13}C NMR** δ (126 MHz, $CDCl_3$) δ 162.1 (C), 161.5 (C), 144.1 (CH), 141.5 (CH), 139.9 (CH), 133.3 (C), 129.6 (CH), 129.4 (CH), 113.4 (CH), 112.6 (CH), 91.2 (C), 87.9 (C), 79.6 (CH),* 76.5 (CH), 57.1 (CH_3), 41.9 (C), 34.4 (CH_2), 31.1 (CH_2), 19.3 (CH_3), 18.7 (CH_3), 18.1 (CH_3); ***m/z*** (EI) 770 ($[M]^+$, 10%), 647 (84), 258 (69), 69 (100); **HRMS** (EI) $[M]^+$ found 770.3800, $C_{44}H_{54}N_2O_{10}$ requires 770.3773. ^{1}H and ^{13}C NMR spectroscopic data in good agreement with the literature.[10]
* one CH obscured by $CDCl_3$

Disorazole C_1 1

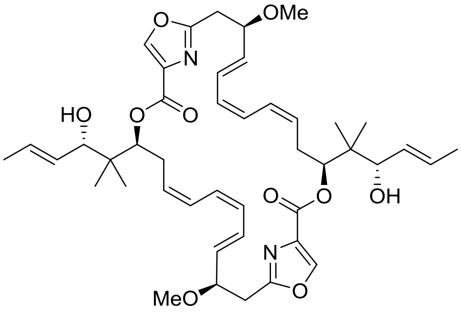

A flask containing a mixture of (9,9',10,10')-tetradehydrido-disorazole C_1 (1.8 mg, 2.3 μmol), quinoline (8.0 μL, 85.0 μmol) and Lindlar catalyst (5% Pd on $CaCO_3$ poisoned with Pb, 1.0 mg) in EtOAc (0.5 mL) was evacuated and flushed with H_2 (3 cycles). The reaction mixture was stirred at rt under H_2 (1 atm) for 6 h and then filtered through a plug of Florisil/Celite (1:1). The plug was rinsed with EtOAc (5 mL) and the combined filtrates were concentrated *in vacuo*. Purification by RP HPLC (isocratic 40:60 H_2O:MeCN) gave recovered starting material (~0.8 mg, R_t = 12.6 min), a peak corresponding to partial reduction of the alkyne (identified by *m/z*, R_t = 17.0 min), and product **1** (~0.2 mg). **R_t** (40:60 H_2O:MeCN) = 20.5 min; **^{1}H NMR** δ (800 MHz, CD_3OD)* 8.24 (2H, s, NC=C*H*), 6.52 (2H, dd, J = 15.2, 11.2 Hz,

CH(OCH_3)CH=C*H*), 6.42 (2H, t, *J* = 11.2 Hz, CH_2CH=C*H*), 6.31 (2H, t, *J* = 11.2 Hz, CHC*H*=CHCH), 5.94 (2H, t, *J* = 11.2 Hz, CHCH=C*H*CH), 5.69 (2H, dq, *J* = 15.0, 6.4 Hz, CH_3C*H*=CH), 5.60 (2H, ddq, *J* = 15.0, 8.1, 1.5 Hz, CH_3CH=C*H*), 5.57 (2H, dd, *J* = 15.2, 8.6 Hz, CH(OCH_3)C*H*=CH), 5.51 (2H, br td, *J* = 10.3, 6.4Hz, CH_2C*H*=CH), 5.28 (2H, dd, *J* = 11.3, 2.3 Hz, C*H*OCOAr), 4.14 (2H, td, *J* = 7.7, 5.6 Hz, C*H*OCH_3), 3.86 (2H, d, *J* = 8.1 Hz, C*H*OH),** 3.00 (2H, dd, *J* = 15.4, 7.3 Hz, C$H_C$$H_DCHOCH_3$), 2.77 (2H, dd, *J* = 15.4, 5.6 Hz, C$H_C$$H_DCHOCH_3$), 2.71 (2H, dt, *J* = 13.9, 10.7 Hz, C$H_E$$H_F$CH=CH), 2.41 (2H, dd, *J* = 13.9, 6.4 Hz, C$H_E$$H_F$CH=CH), 1.72 (6H, dd, *J* = 6.4, 1.5, C*H*$_3$CH), 1.01 (6H, s, CC*H*$_3$), 0.95 (6H, s, CC*H*$_3$); ***m/z*** (ESI+, MeOH/H_2O)* 829 ($[M+MeOH+Na]^+$, 21%), 811 (39), 797 ($[M+Na]^+$, 100), 508 (12), 425 (46), 409 (8); **HRMS** (ESI, MeOH/H_2O) $[M+H]^+$ found 797.3976, $C_{44}H_{58}N_2O_{10}Na$ requires 797.3984. ^1H NMR spectroscopic data in good agreement with the literature.[10]

* ^1H and MS spectra contaminated with solvents, and unknown plastizer[11] or components of the detergent Triton X101R.[12]

** two CH_3 obscured by CD_3OD

References and Notes:

[1] G.-E. Benoit, J. S. Carey, A. M. Chapman, R. Chima, N. Hussain, M. E. Popkin, G. Roux, B. Tavassoli, C. Vaxelaire, M. R. Webb, D. Whatrup, *Org. Process Res. Dev.* **2008**, *10*, 2813-2816.

[2] a) W. Boland, N. Schroer, C. Sieler, M. Feigel, *Helv. Chim. Acta* **1987**, *70*, 1025-1040; b) U. Koop, G. Handke, N. Krause, *Liebigs Ann. Chem.* **1996**, 1487-1499.

[3] K. C. Babu, R. Vysabhattar,K. S. V. Srinivas, S. Nigam, G. Madhusudhan, K. Mukkanti, *Tetrahedron: Asymm.* **2010**, *21*, 2619-2624.

[4] S. Bresciani, A. M. Z. Slawin, D. O'Hagan, *J. Fluorine Chem.* **2009**, *130*, 537-543.

[5] O. W. Gooding, C. C. Beard, D. Y. Jackson, D. L. Wren, G. F. Cooper, *J. Org. Chem.* **1991**, *56*, 1083-1088.

[6] a) I. V. Hartung, B. Niess, L. O. Haustedt, H. M. R. Hoffmann, *Org. Lett.* **2002**, *4*, 3239–3242; b) I. V. Hartung, *Stereoselective Synthesis of the Northern and Southern Half of the Antimitotic Agent Disorazole A1 and Studies on its Biosynthesis*, PhD Thesis, Gottfried Wilhelm Leibniz Universität Hannover **2002** (German).

[7] G. Stork, K. Zhao, *Tetrahedron Lett.* **1989**, *30*, 2173–2174.

[8] E. A. Crane, T. P. Zabawa, R. L. Farmer, K. A. Scheidt, *Angew. Chem. Int. Ed.* **2011**, *50*, 9112–9115.

[9] A. N. Rai, A. Basu, *Tetrahedron Lett.* **2003**, *44*, 2267–2269.

[10] P. Wipf, T. H. Graham, *J. Am. Chem. Soc.* **2004**, *126*, 15346-15347.

[11] Identified using the Waters database at: https://www.waters.com/webassets/cms/support/docs/bkgrnd_ion_mstr_list.pdf

[12] Identified using the MaConDa mass spectrometry contaminants database at: http://www.maconda.bham.ac.uk/index.php

**Page**	**^{1}H and ^{13}C spectra for:**
S25-S34	Synthesis of oxazole-containing acid **28**
S35-S45	Synthesis of β-hydroxy ketone fragment **5**
S46-S50	Evans-Tishchenko and fragment coupling to give bis-alkynes **3** and **29**
S51-S55	Self-assembly metathesis reaction to give di-lactones **2**, **34**; PMB deprotection to give **35** and Lindlar hydrogenation to give **1**

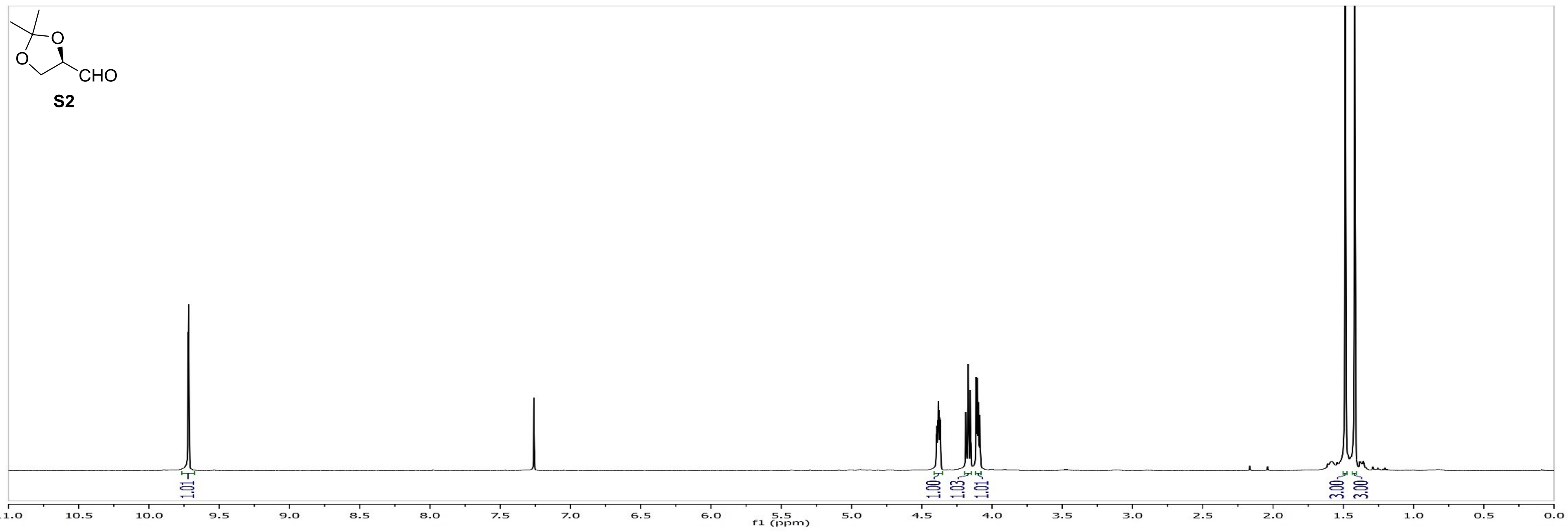
O
O
CHO
S2
1.01
1.00
1.03
1.01
3.00
3.00
11.0 10.5 10.0 9.5 9.0 8.5 8.0 7.5 7.0 6.5 6.0 5.5 5.0 4.5 4.0 3.5 3.0 2.5 2.0 1.5 1.0 0.5 0.0
f1 (ppm)

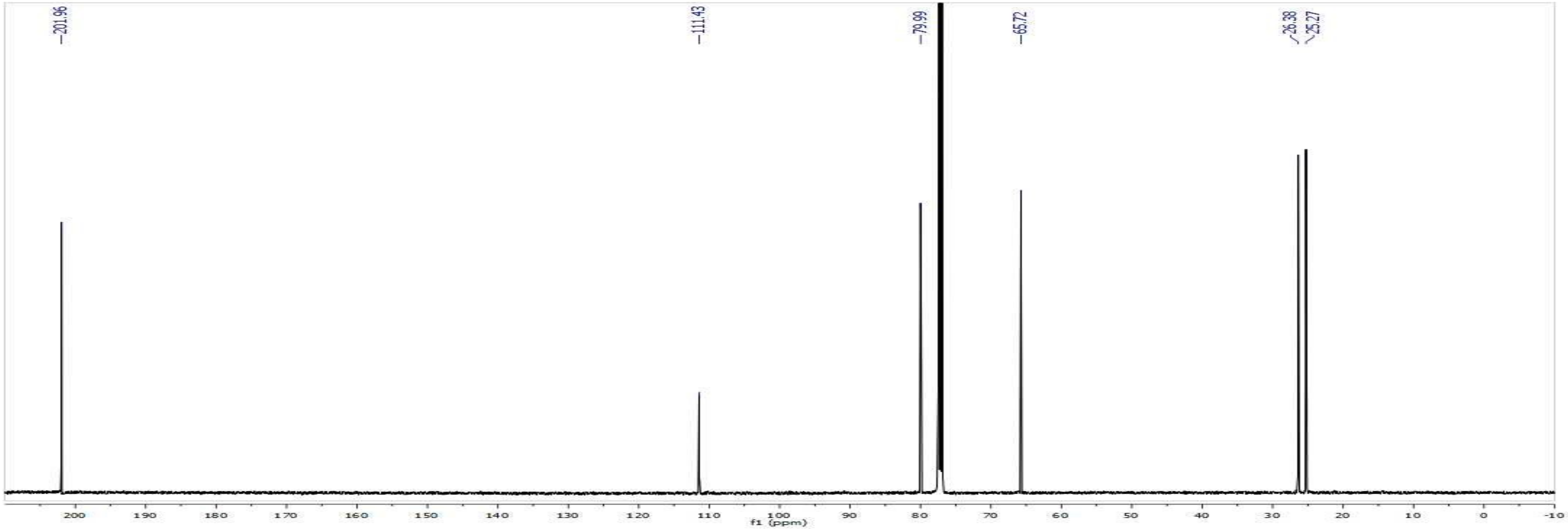
201.96
111.43
79.99
65.72
26.38
25.27
200 190 180 170 160 150 140 130 120 110 100 90 80 70 60 50 40 30 20 10 0 -10
f1 (ppm)

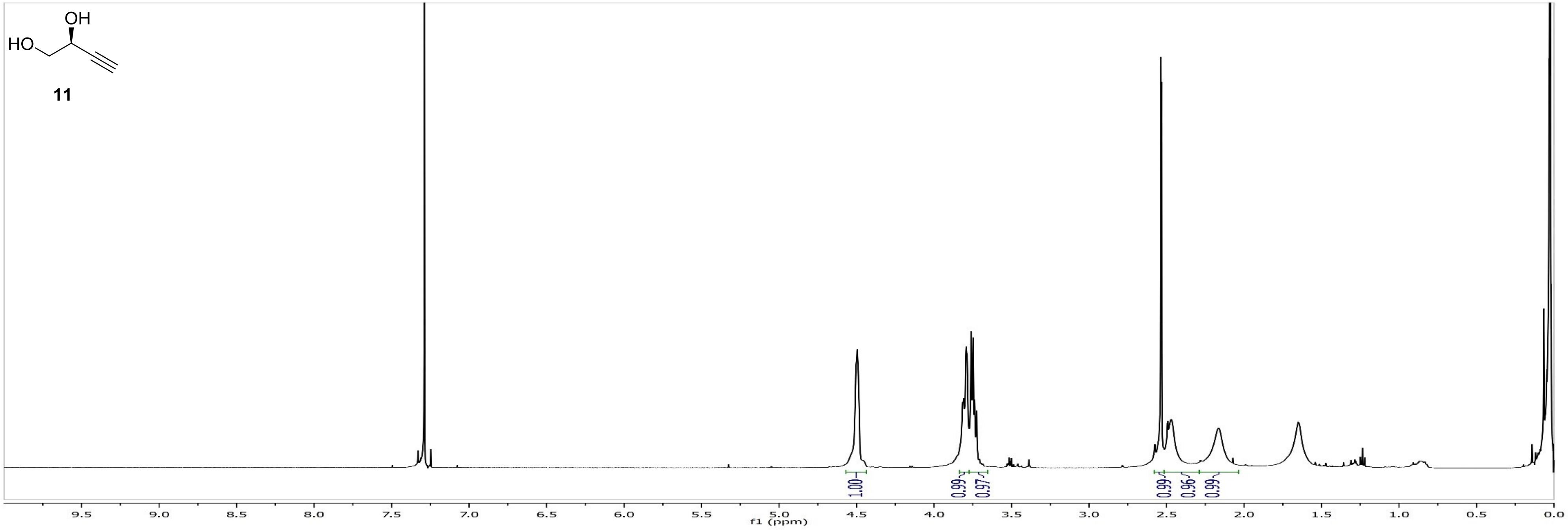
OH
HO
11
1.00
0.99
0.97
0.99
0.96
0.99
9.5 9.0 8.5 8.0 7.5 7.0 6.5 6.0 5.5 5.0 4.5 4.0 3.5 3.0 2.5 2.0 1.5 1.0 0.5 0.0
f1 (ppm)

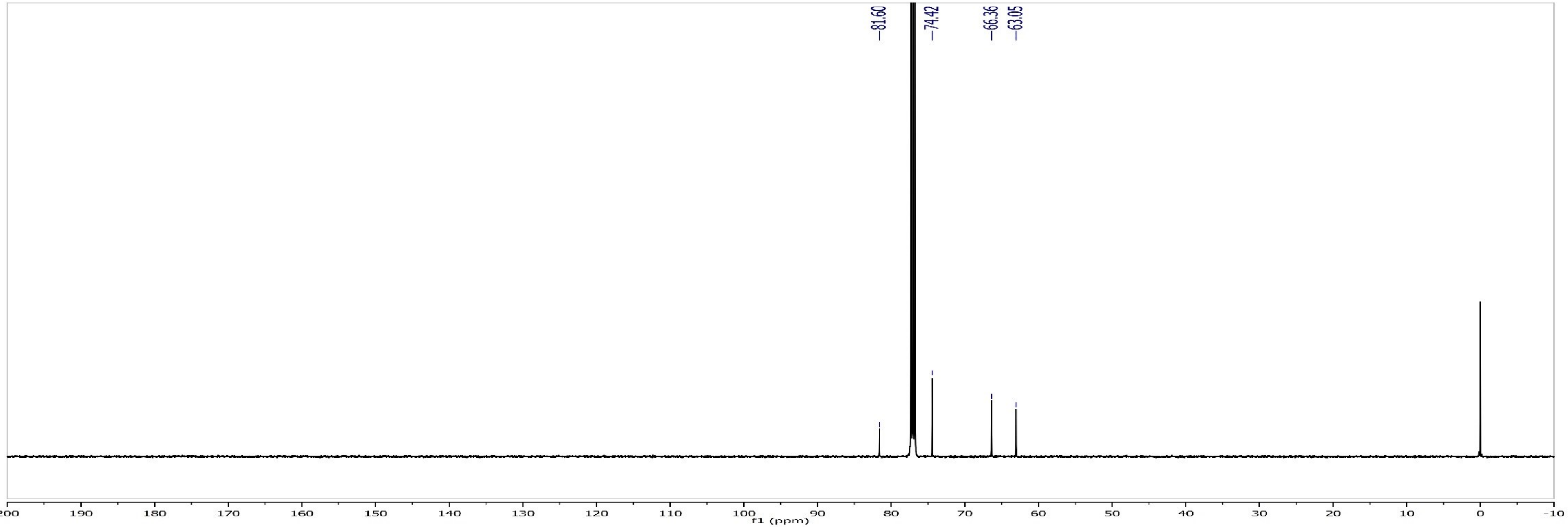
81.60
74.42
66.36
63.05
200 190 180 170 160 150 140 130 120 110 100 90 80 70 60 50 40 30 20 10 0 -10
f1 (ppm)

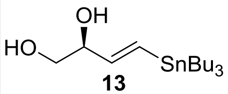
OH
HO
SnBu3

13

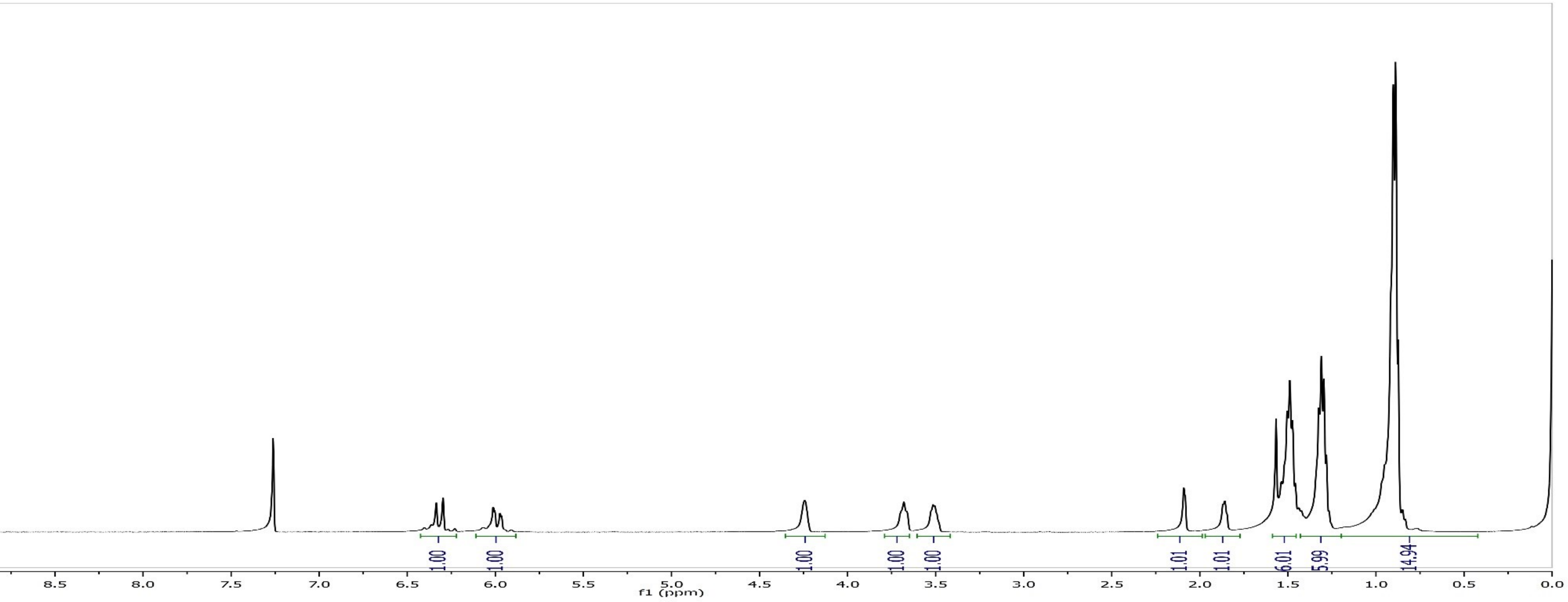
1.00
1.00
1.00
1.00
1.00
1.01
1.01
6.01
5.99
14.94
10.0 9.5 9.0 8.5 8.0 7.5 7.0 6.5 6.0 5.5 5.0 4.5 4.0 3.5 3.0 2.5 2.0 1.5 1.0 0.5 0.0
f1 (ppm)

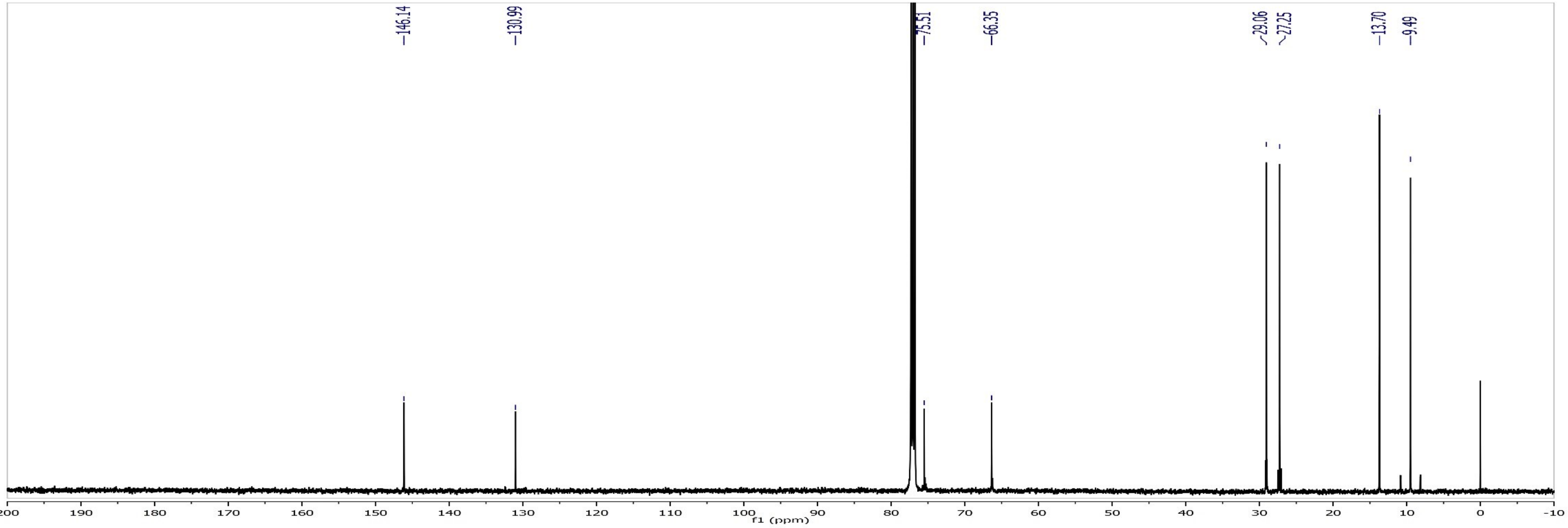
146.14
130.99
75.51
66.35
29.06
27.25
13.70
9.49
200 190 180 170 160 150 140 130 120 110 100 90 80 70 60 50 40 30 20 10 0 -10
f1 (ppm)

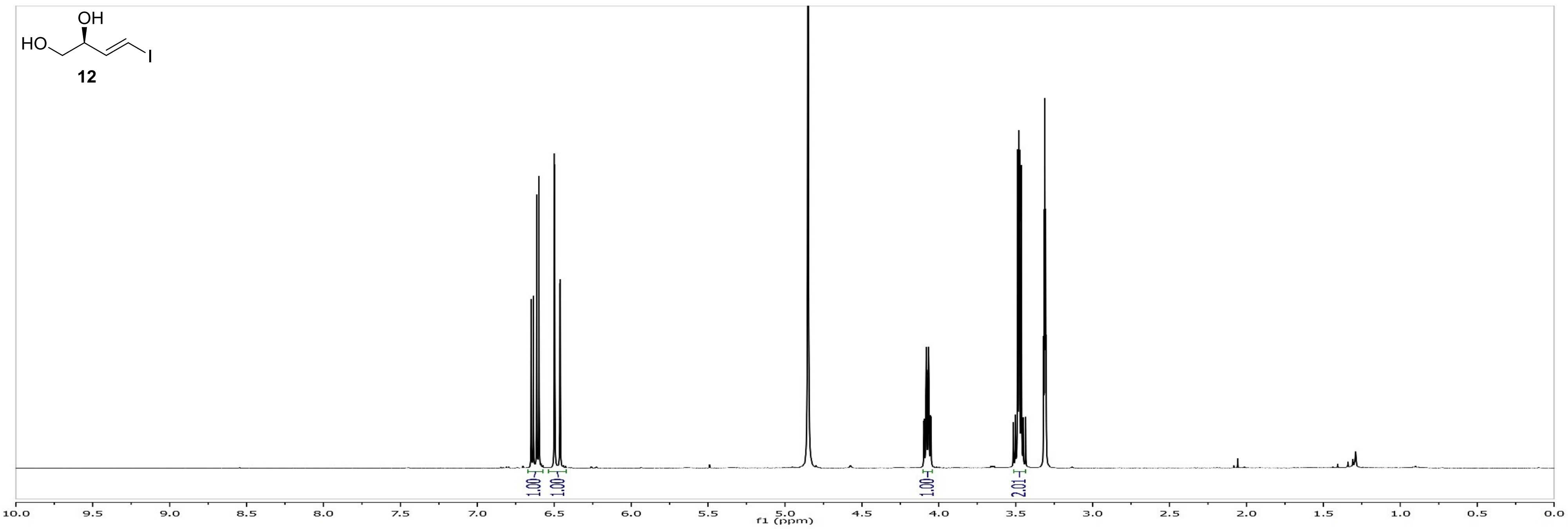
OH
HO
I
12
1.00
1.00
1.00
2.01
10.0
9.5
9.0
8.5
8.0
7.5
7.0
6.5
6.0
5.5
5.0
4.5
4.0
3.5
3.0
2.5
2.0
1.5
1.0
0.5
0.0
f1 (ppm)

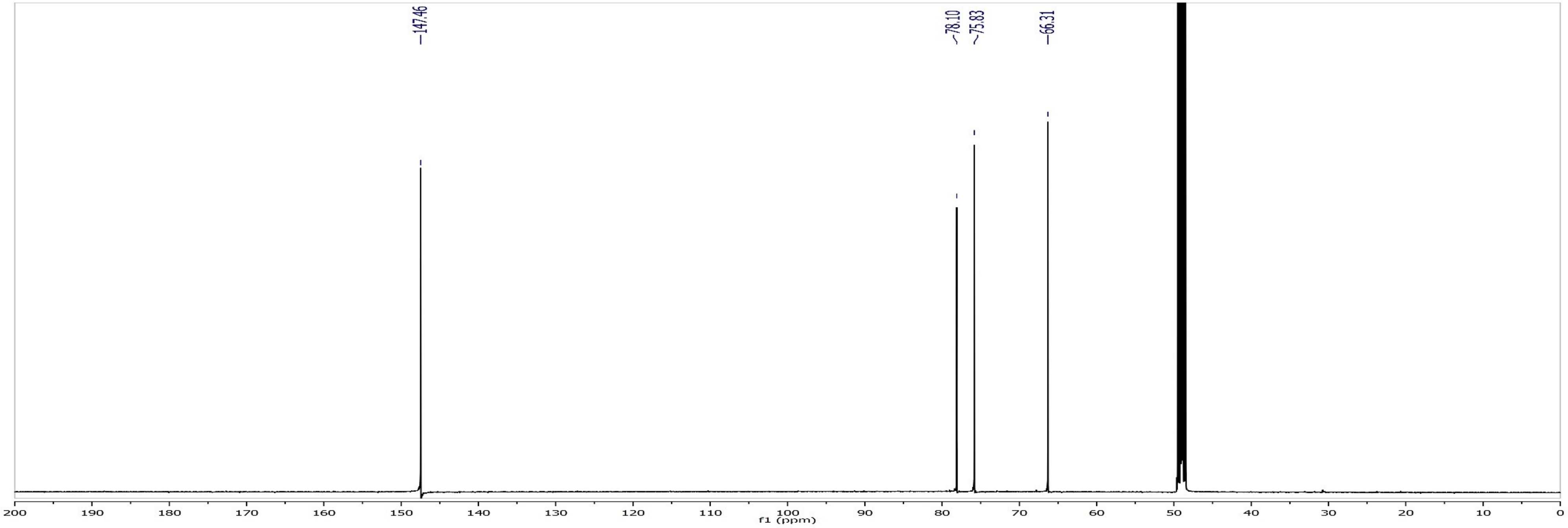
147.46
78.10
75.83
66.31
200
190
180
170
160
150
140
130
120
110
100
90
80
70
60
50
40
30
20
10
0
f1 (ppm)

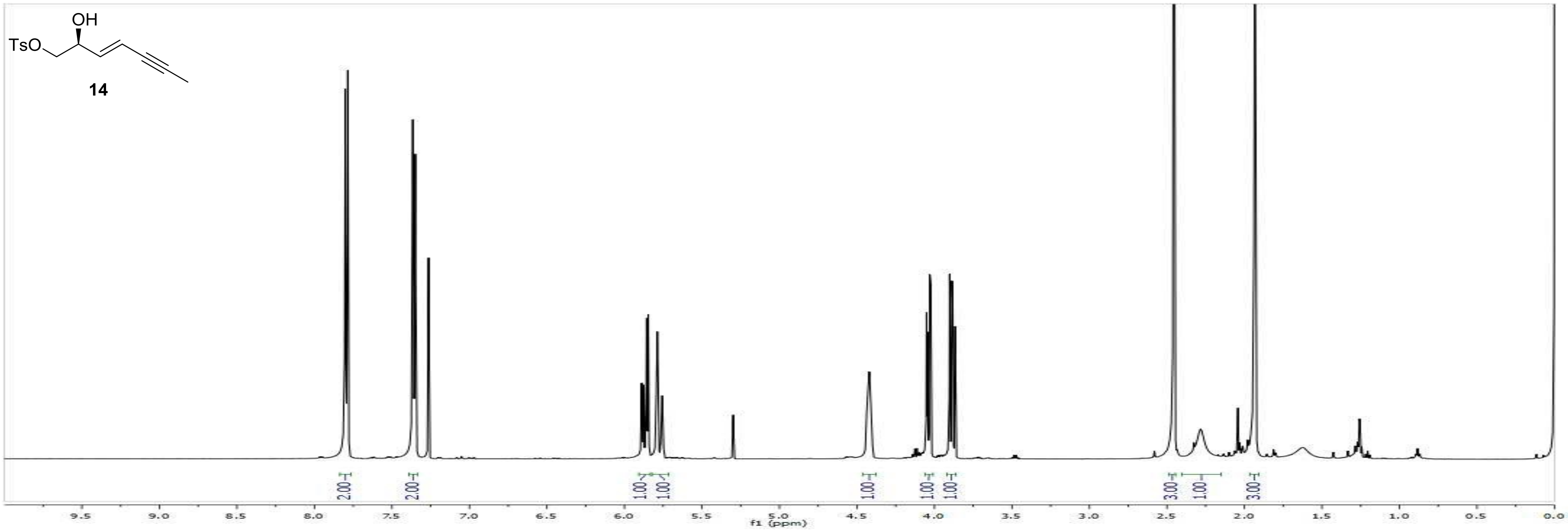
OH
TsO
14
2.00
2.00
1.00
1.00
1.00
1.00
1.00
3.00
1.00
3.00
9.5 9.0 8.5 8.0 7.5 7.0 6.5 6.0 5.5 5.0 4.5 4.0 3.5 3.0 2.5 2.0 1.5 1.0 0.5 0.0
f1 (ppm)

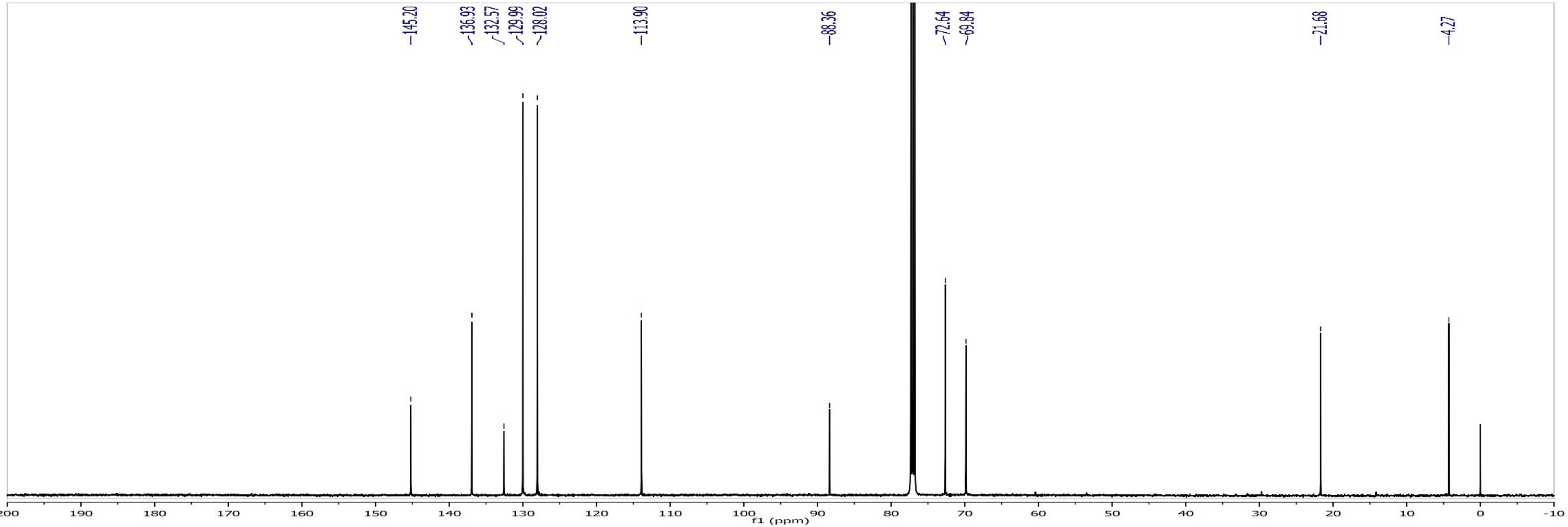
145.20
136.93
132.57
129.99
128.02
113.90
88.36
72.64
69.84
21.68
4.27
200 190 180 170 160 150 140 130 120 110 100 90 80 70 60 50 40 30 20 10 0 -10
f1 (ppm)

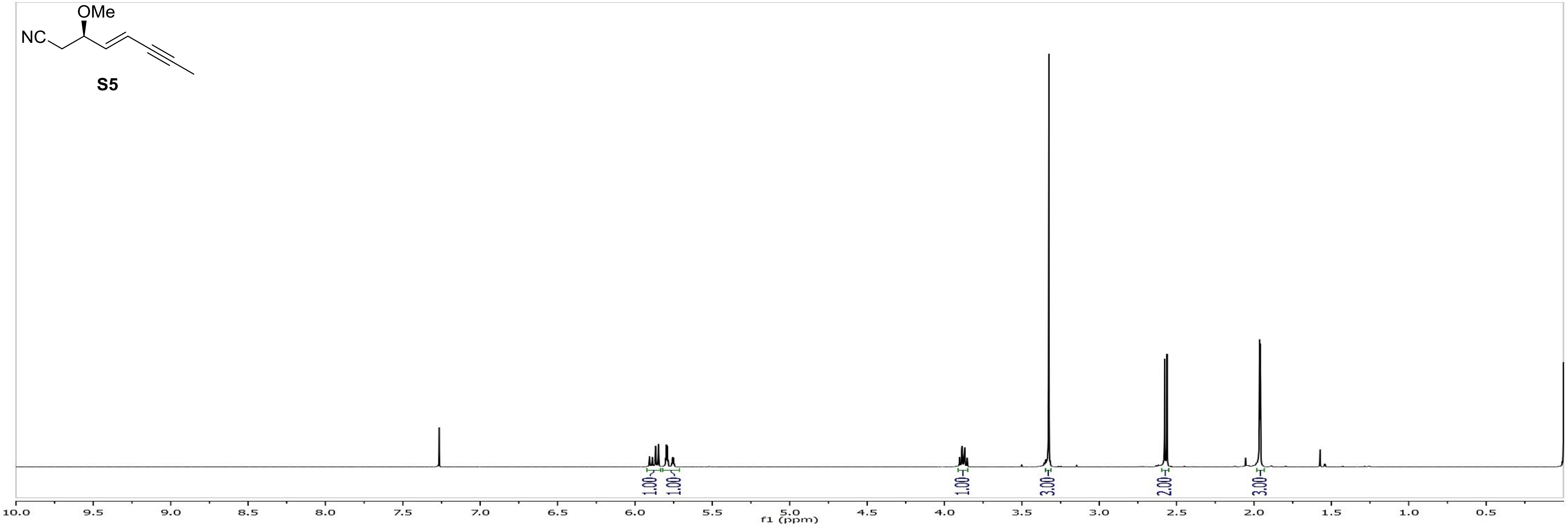
OMe
NC
S5
1.00
1.00
1.00
3.00
2.00
3.00
10.0
9.5
9.0
8.5
8.0
7.5
7.0
6.5
6.0
5.5
5.0
f1 (ppm)
4.5
4.0
3.5
3.0
2.5
2.0
1.5
1.0
0.5

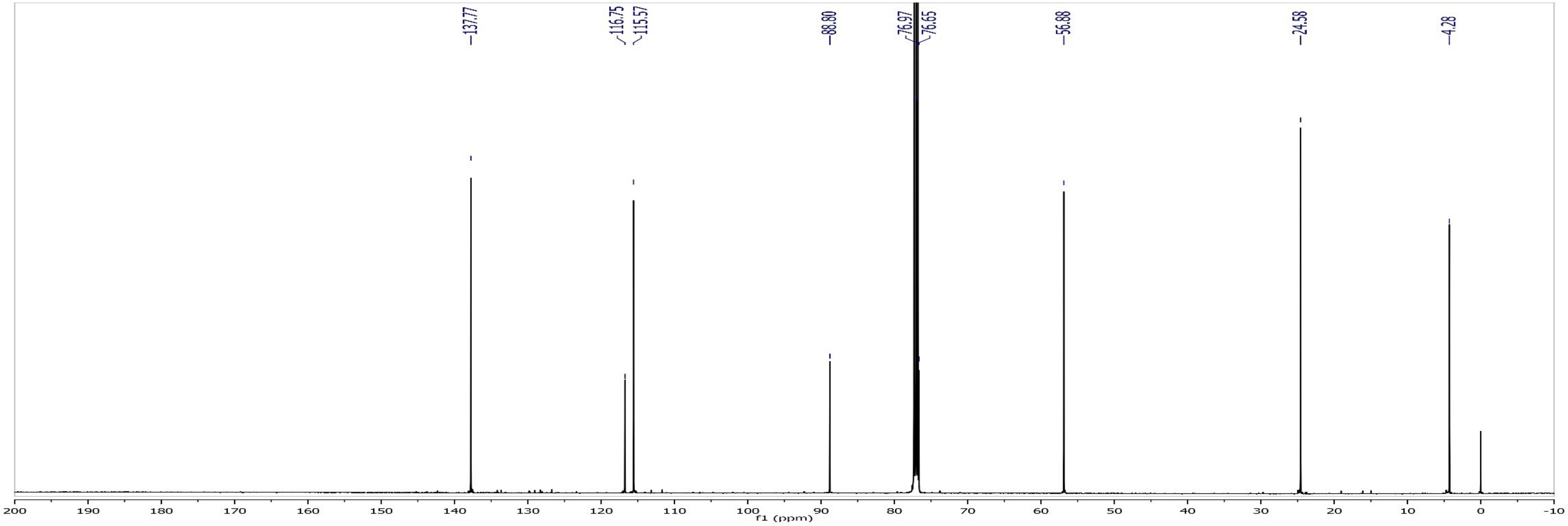
137.77
116.75
115.57
88.80
76.97
76.65
56.88
24.58
4.28
200
190
180
170
160
150
140
130
120
110
100
f1 (ppm)
90
80
70
60
50
40
30
20
10
0
-10

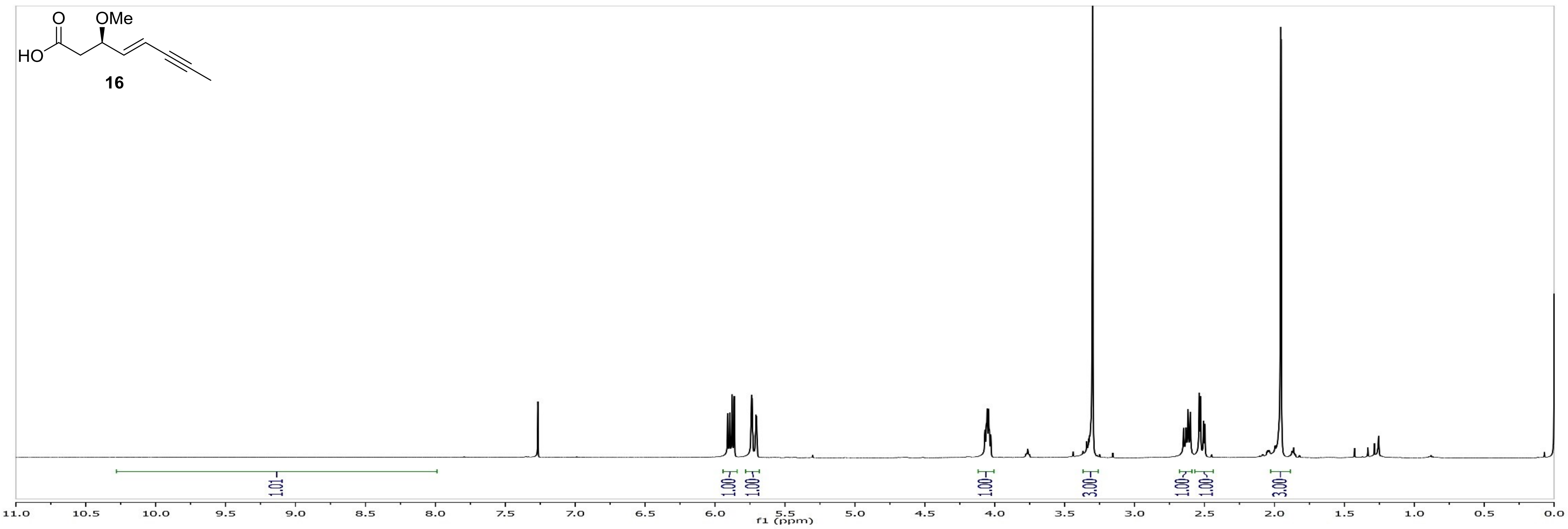

O
OMe
HO
16
1.01
1.00
1.00
1.00
3.00
1.00
1.00
3.00
f1 (ppm)

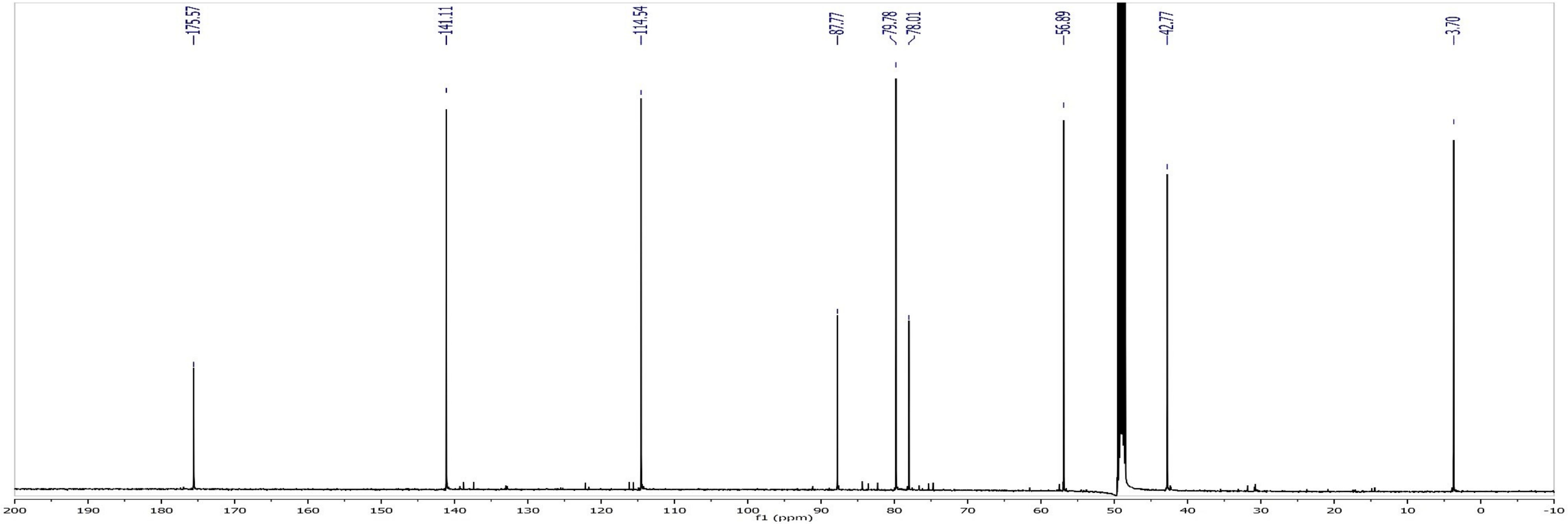

175.57
141.11
114.54
87.77
79.78
78.01
56.89
42.77
3.70
f1 (ppm)

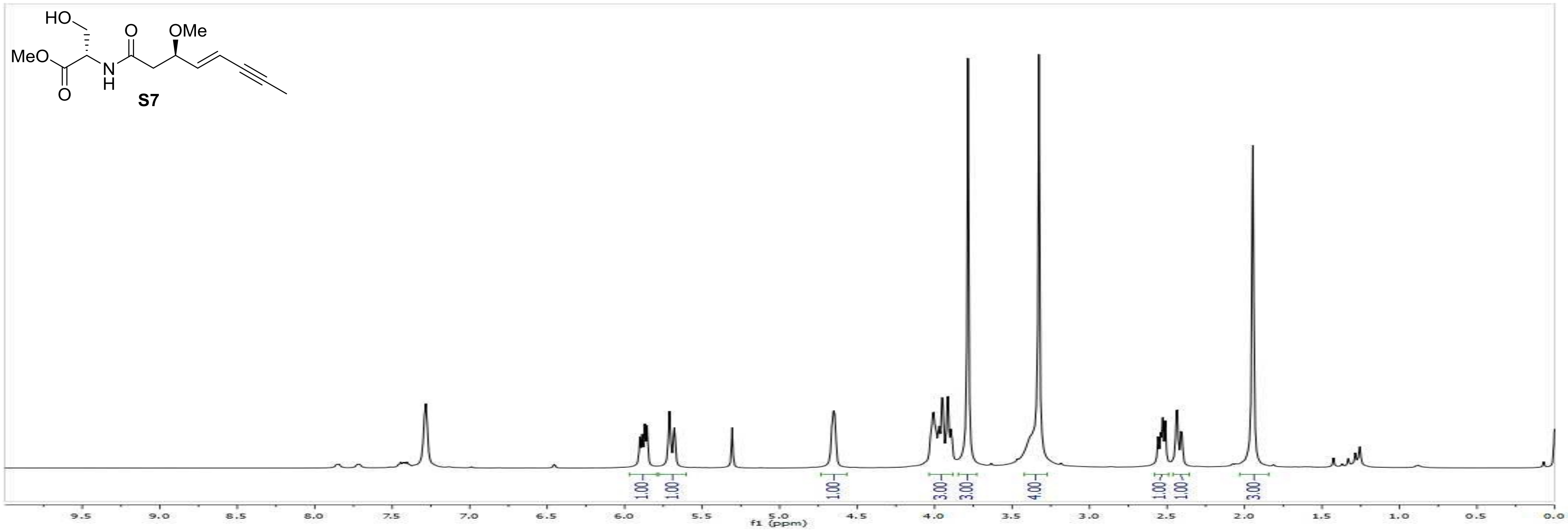
HO
MeO
O
N
H
O
OMe
S7
1.00
1.00
1.00
3.00
3.00
4.00
1.00
1.00
3.00
f1 (ppm)

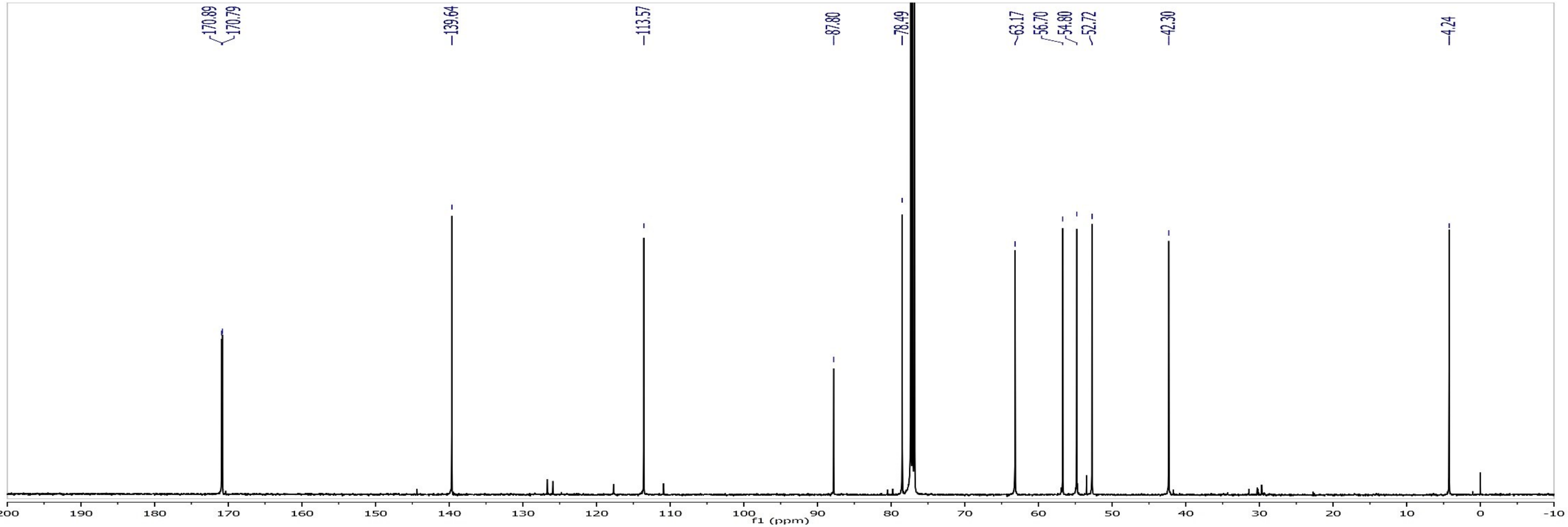
170.89
170.79
139.64
113.57
87.80
78.49
63.17
56.70
54.80
52.72
42.30
4.24
f1 (ppm)

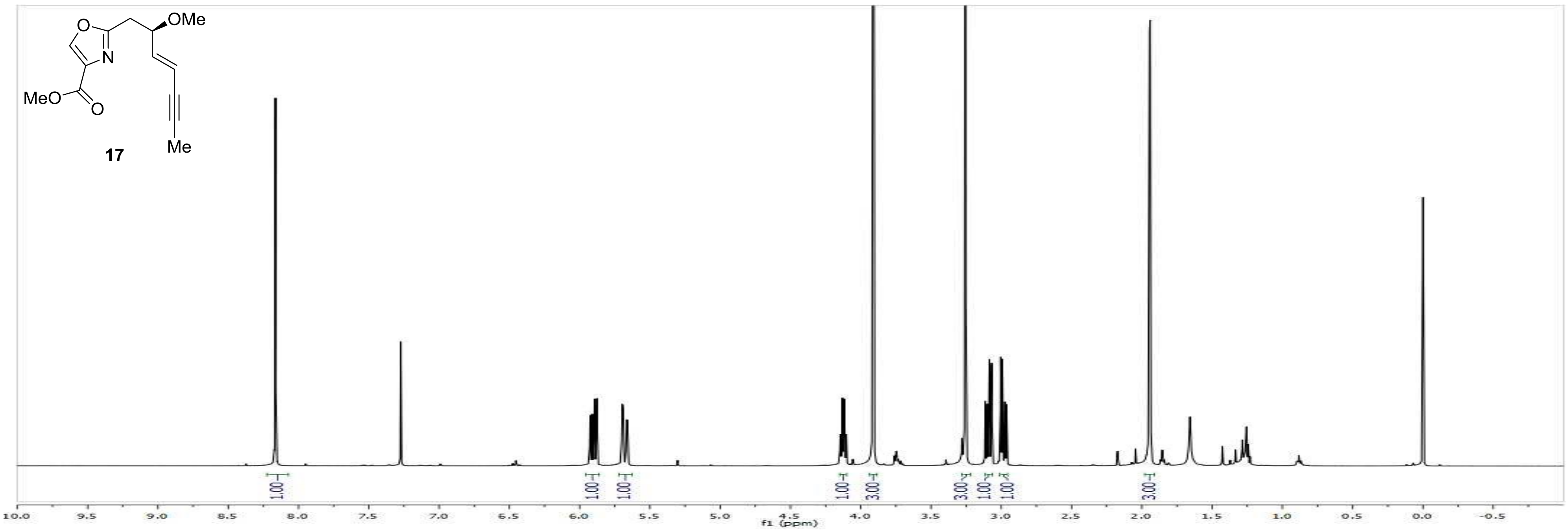
OMe
MeO
O
N
17
Me
1.00
1.00
1.00
1.00
3.00
3.00
1.00
1.00
3.00
f1 (ppm)

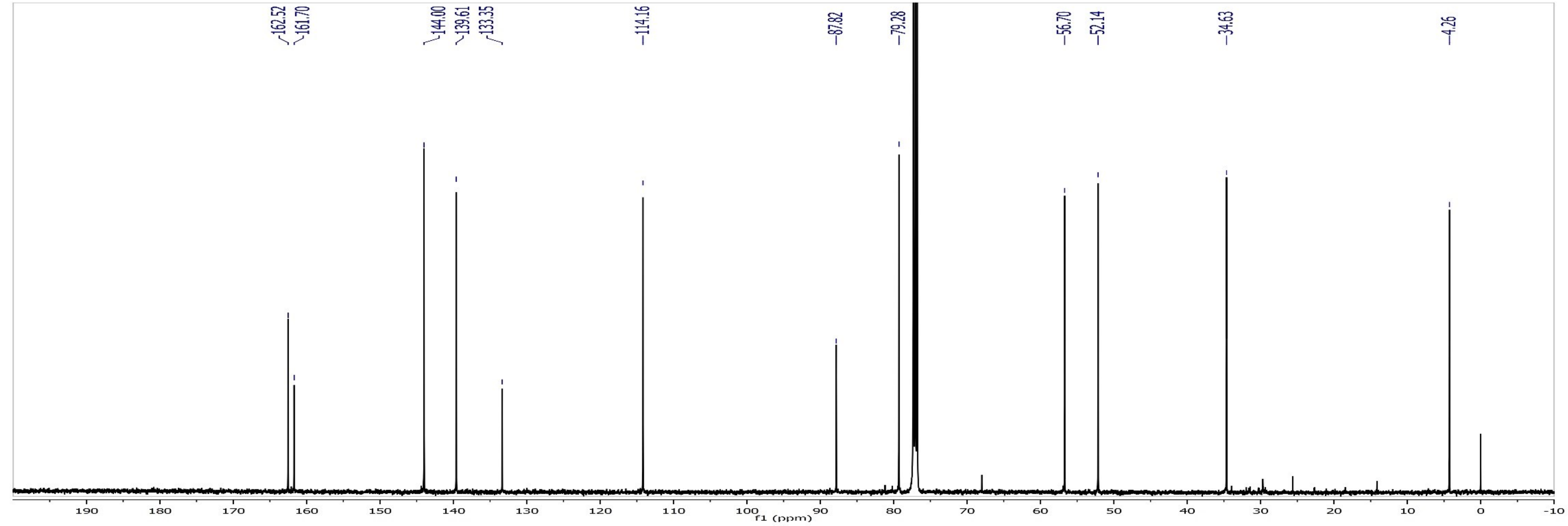
162.52
161.70
144.00
139.61
133.35
114.16
87.82
79.28
56.70
52.14
34.63
4.26
f1 (ppm)

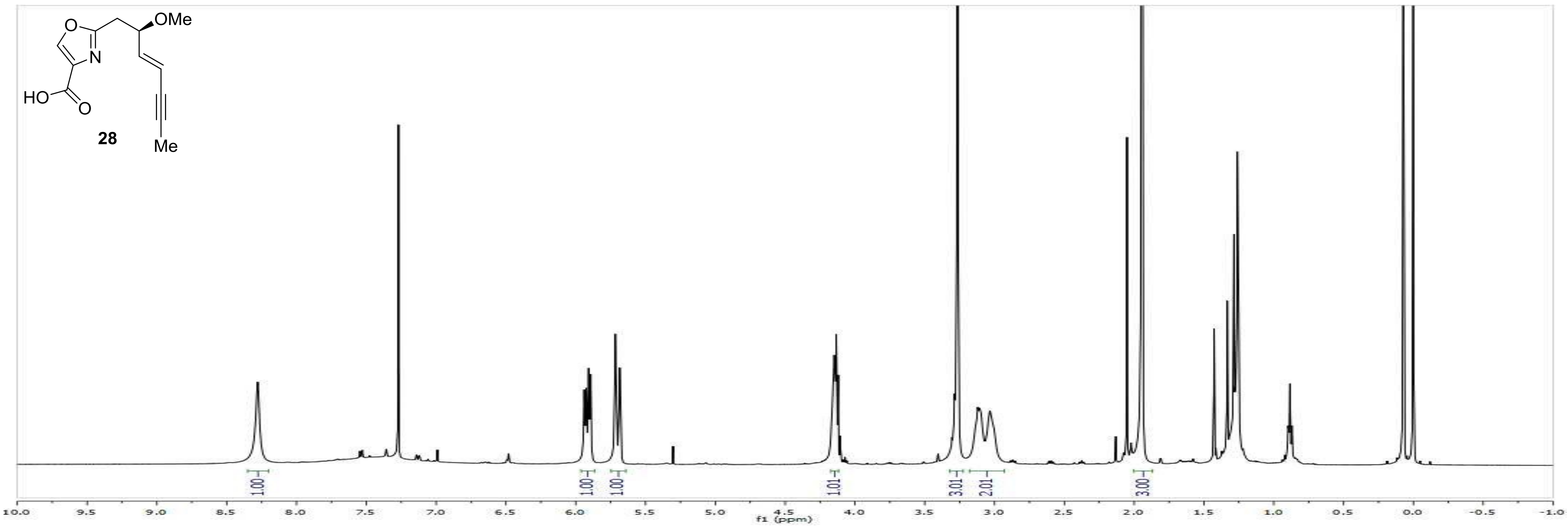
OMe
N
HO
O
28
Me
1.00
1.00
1.00
1.01
3.01
2.01
3.00
f1 (ppm)

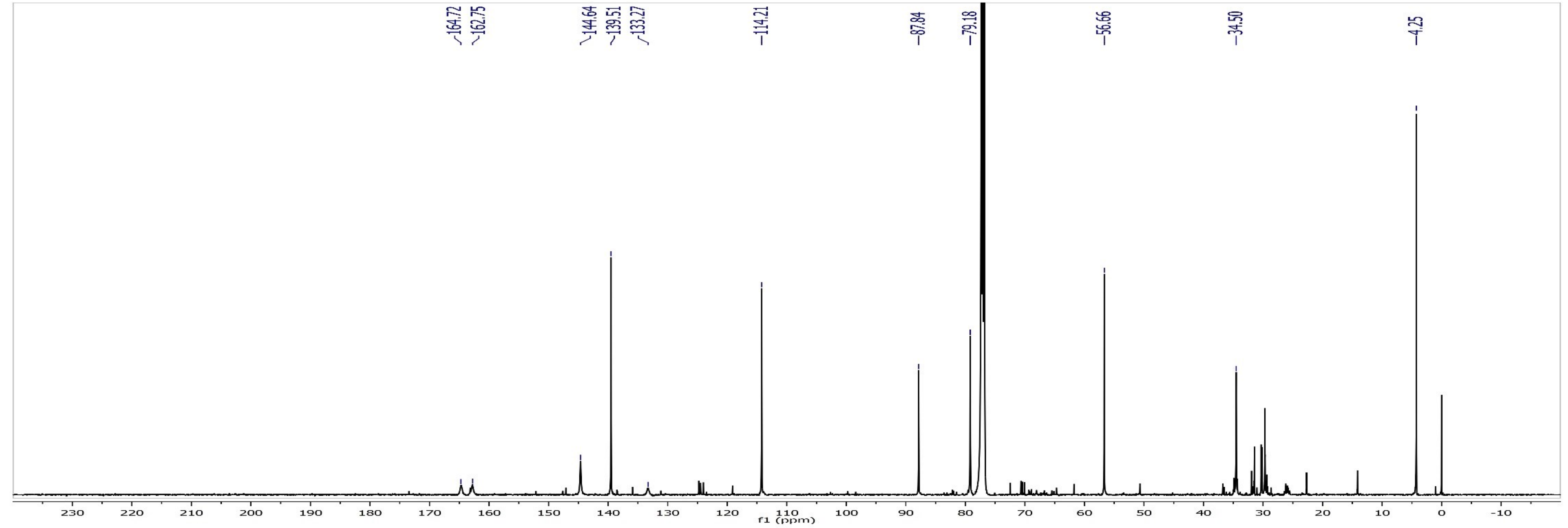
164.72
162.75
144.64
139.51
133.27
114.21
87.84
79.18
56.66
34.50
4.25
f1 (ppm)

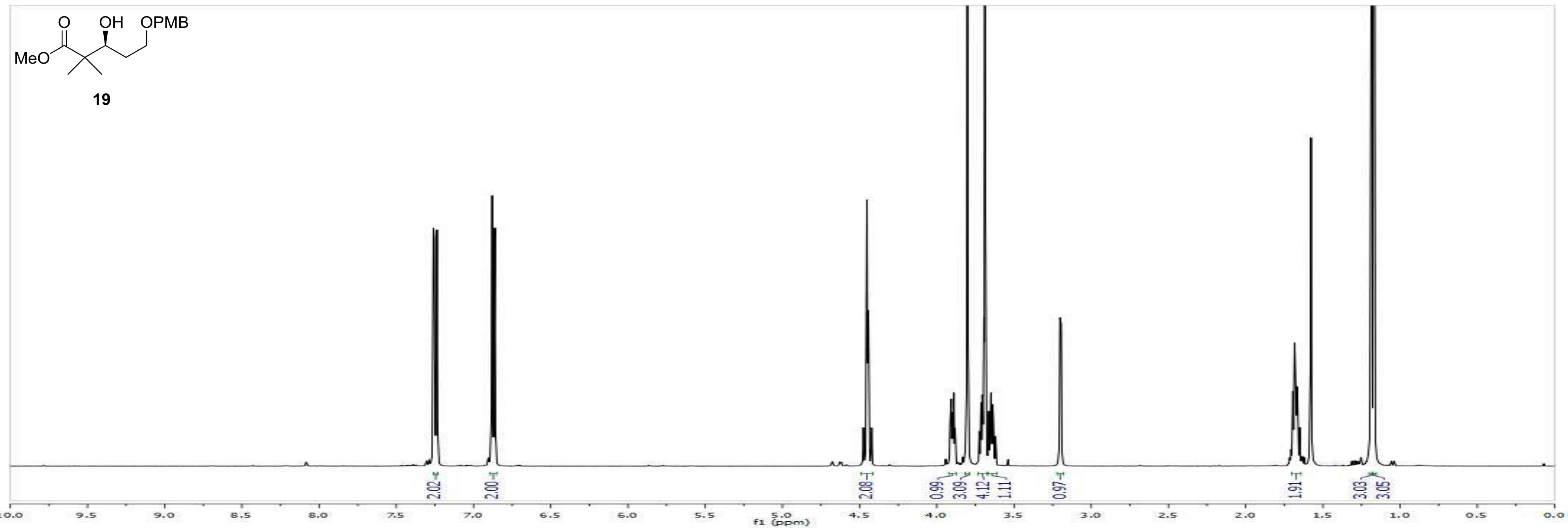
O
OH
OPMB
MeO
19
2.02
2.00
2.08
0.99
3.09
4.12
1.11
0.97
1.91
3.03
3.05
10.0 9.5 9.0 8.5 8.0 7.5 7.0 6.5 6.0 5.5 5.0 4.5 4.0 3.5 3.0 2.5 2.0 1.5 1.0 0.5 0.0
f1 (ppm)

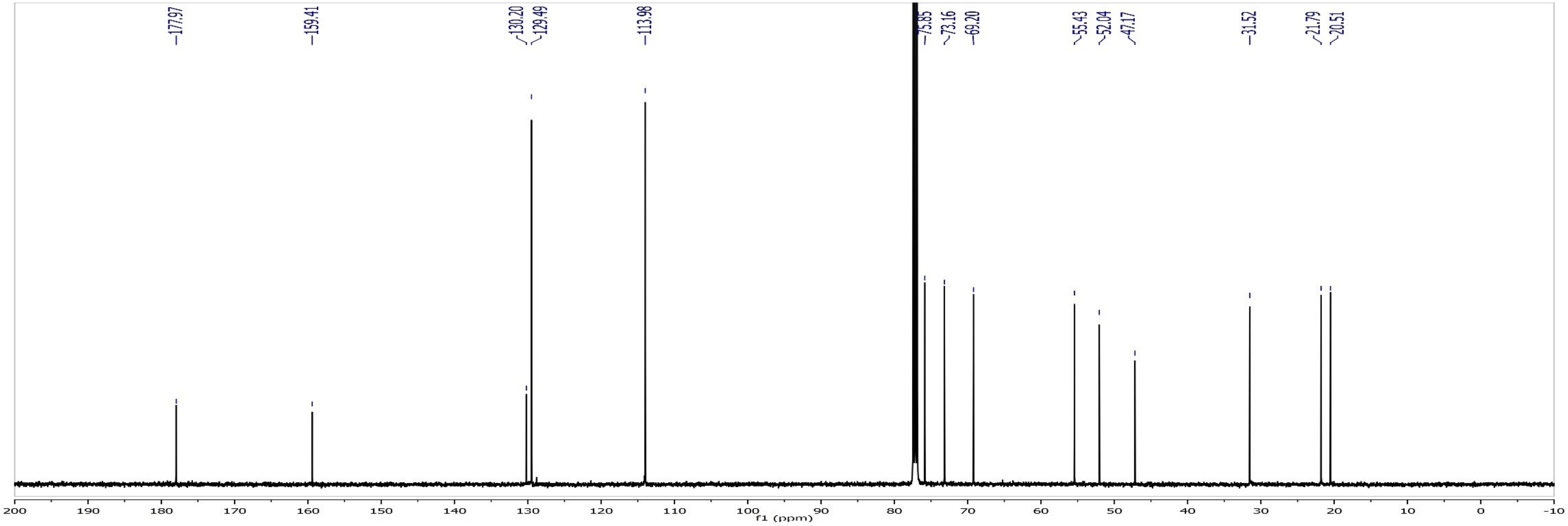
177.97
159.41
130.20
129.49
113.98
75.85
73.16
69.20
55.43
52.04
47.17
31.52
21.79
20.51
200 190 180 170 160 150 140 130 120 110 100 90 80 70 60 50 40 30 20 10 0 -10
f1 (ppm)

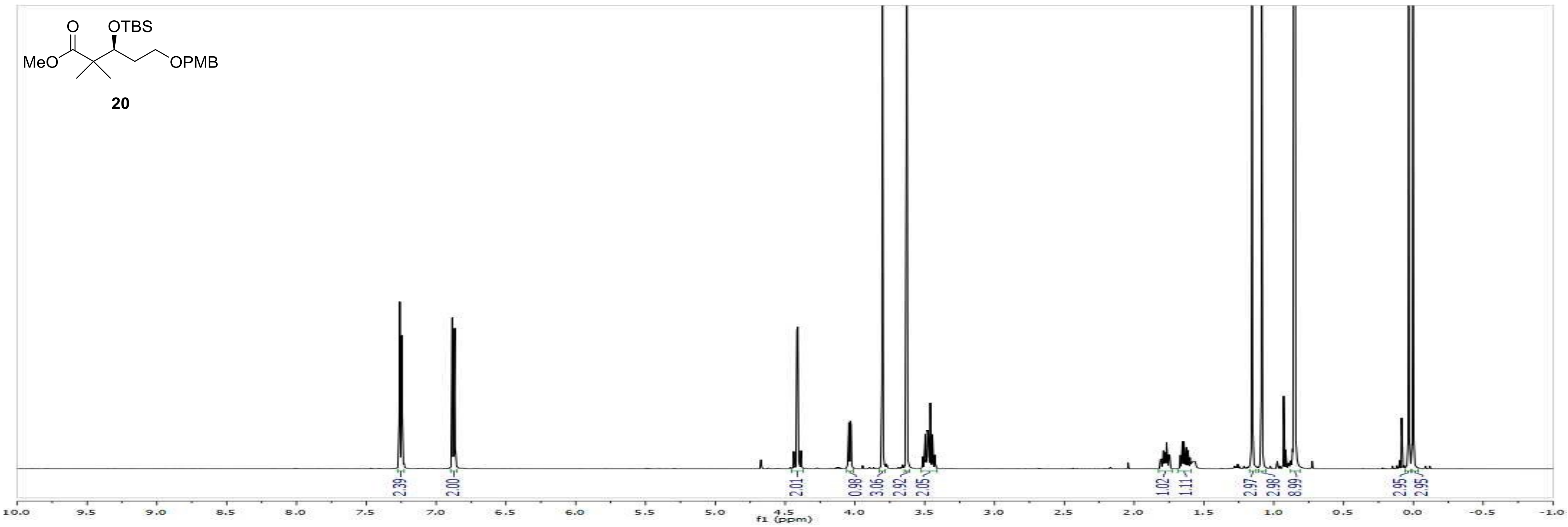
OTBS
MeO
OPMB
20
f1 (ppm)

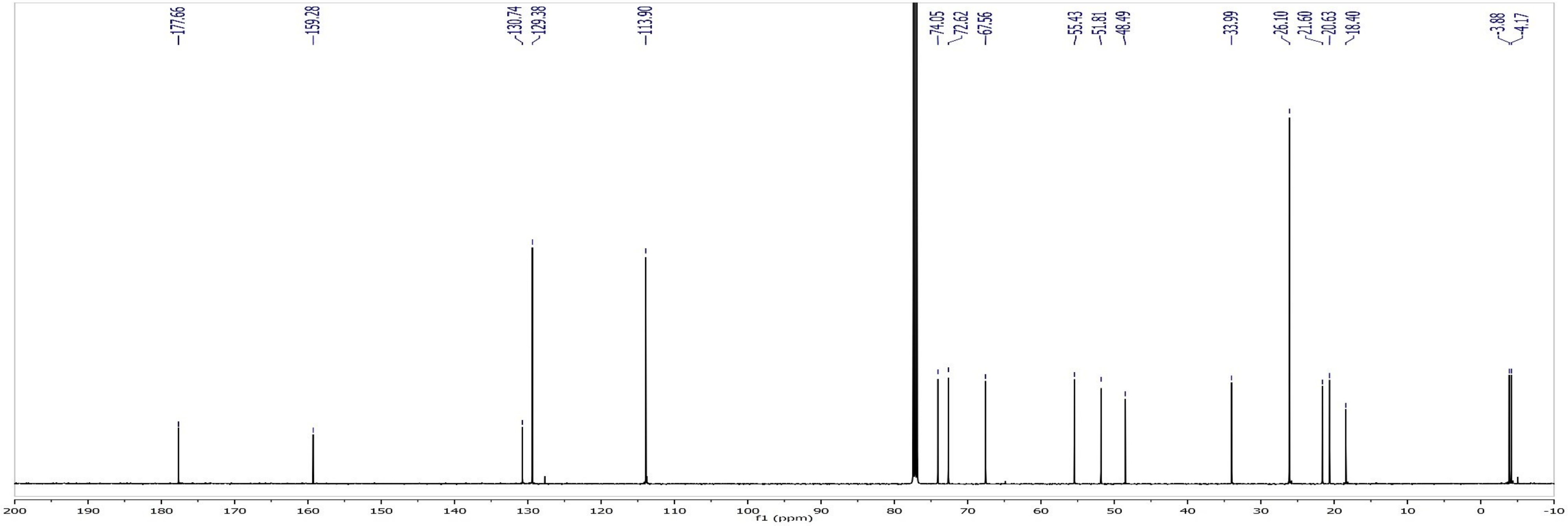
177.66
159.28
130.74
129.38
113.90
74.05
72.62
67.56
55.43
51.81
48.49
33.99
26.10
21.60
20.63
18.40
-3.88
-4.17
f1 (ppm)

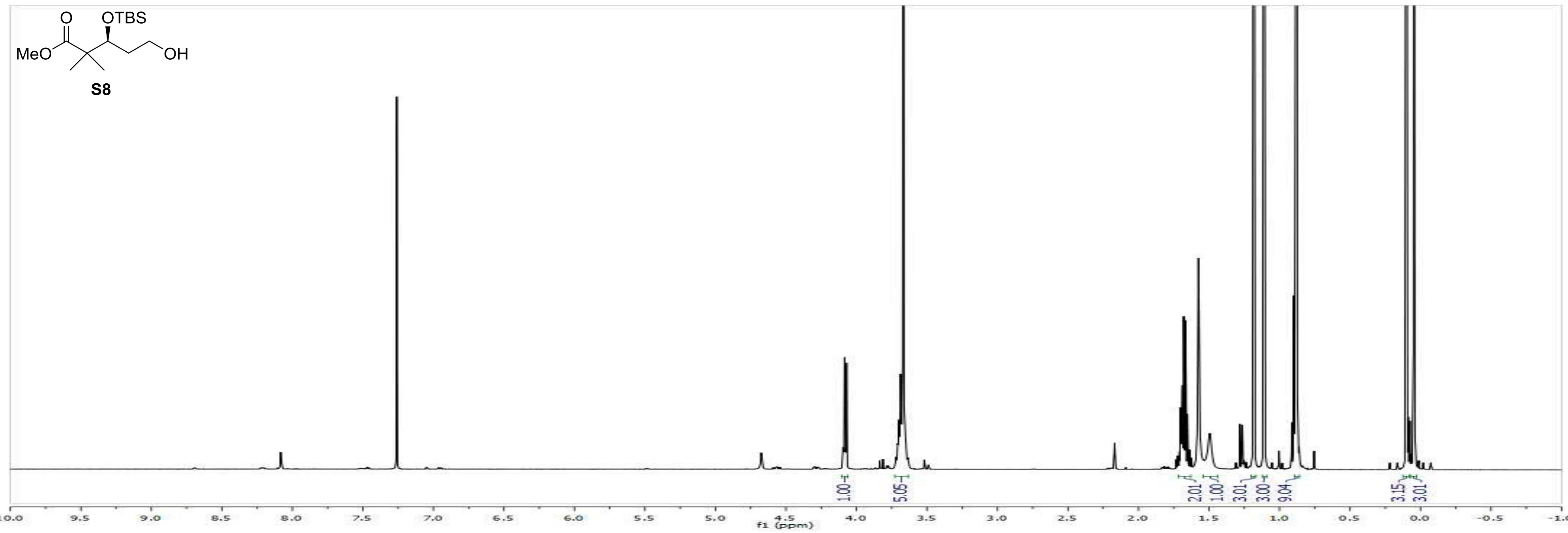

O
OTBS
MeO
OH
S8
1.00
5.05
2.01
1.00
3.01
3.00
9.04
3.15
3.01
10.0 9.5 9.0 8.5 8.0 7.5 7.0 6.5 6.0 5.5 5.0 4.5 4.0 3.5 3.0 2.5 2.0 1.5 1.0 0.5 0.0 -0.5 -1.0
f1 (ppm)

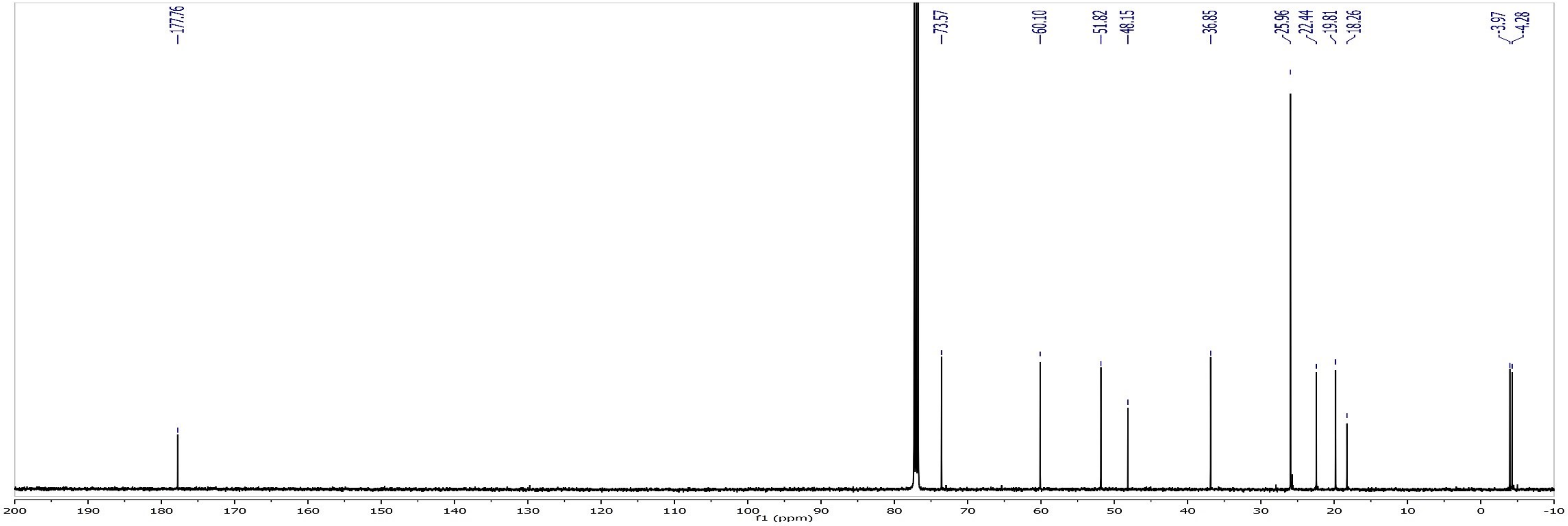

177.76
73.57
60.10
51.82
48.15
36.85
25.96
22.44
19.81
18.26
-3.97
-4.28
200 190 180 170 160 150 140 130 120 110 100 90 80 70 60 50 40 30 20 10 0 -10
f1 (ppm)

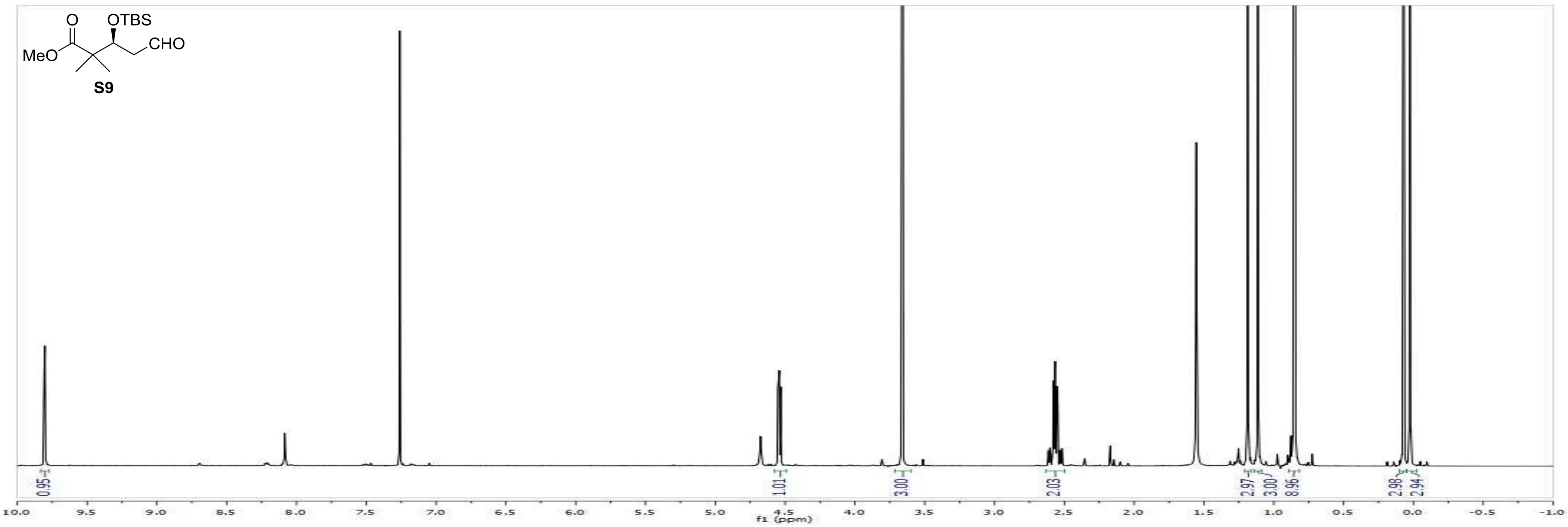
O
OTBS
MeO
CHO
S9
0.95
1.01
3.00
2.03
2.97
3.00
8.96
2.98
2.94
f1 (ppm)

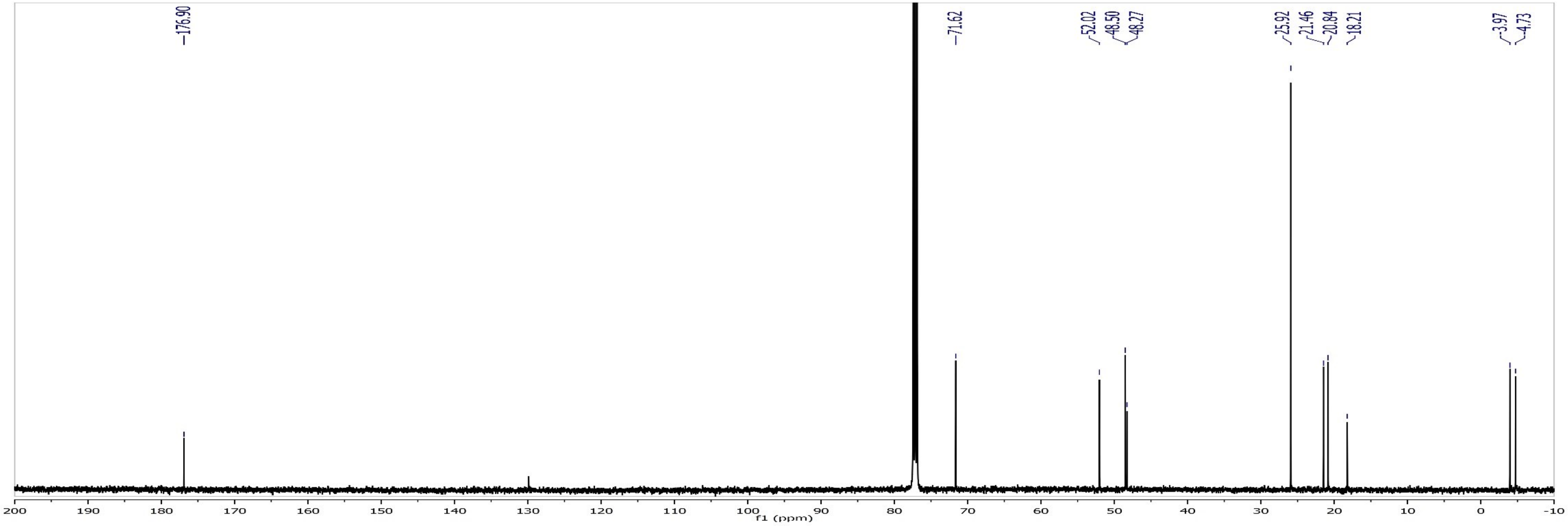
176.90
71.62
52.02
48.50
48.27
25.92
21.46
20.84
18.21
-3.97
-4.73
f1 (ppm)

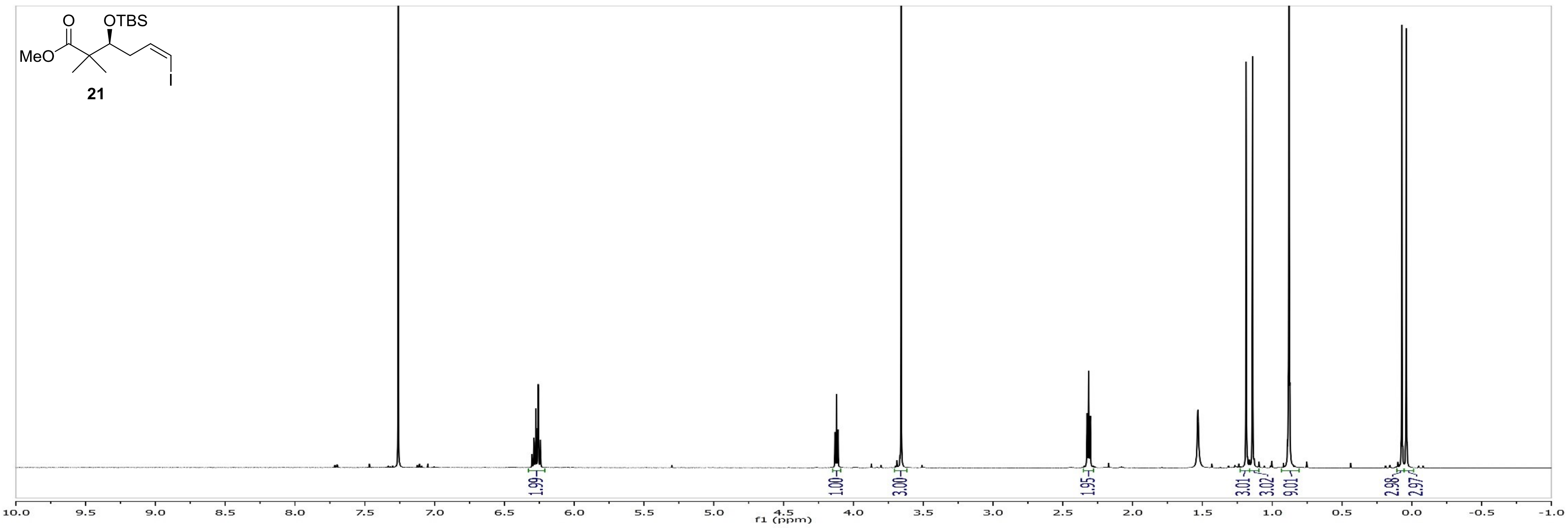
O
OTBS
MeO
I
21
1.99
1.00
3.00
1.95
3.01
3.02
9.01
2.98
2.97
f1 (ppm)

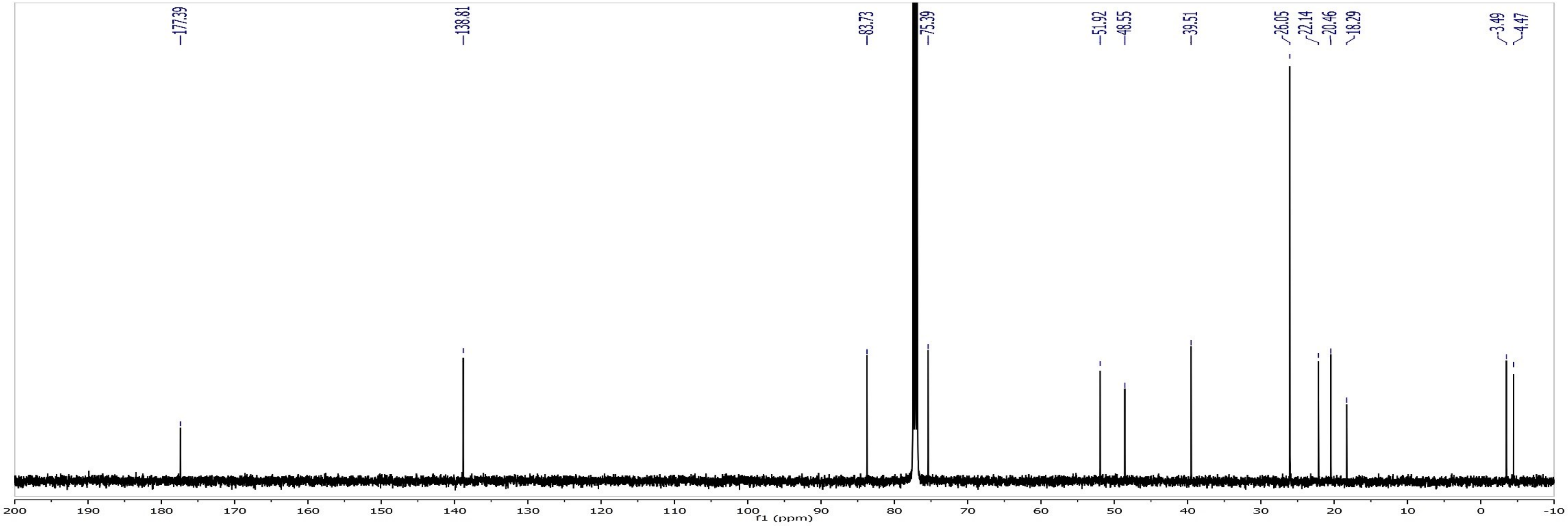
177.39
138.81
83.73
75.39
51.92
48.55
39.51
26.05
22.14
20.46
18.29
-3.49
-4.47
f1 (ppm)

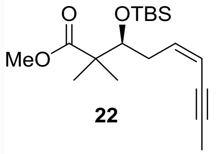

22

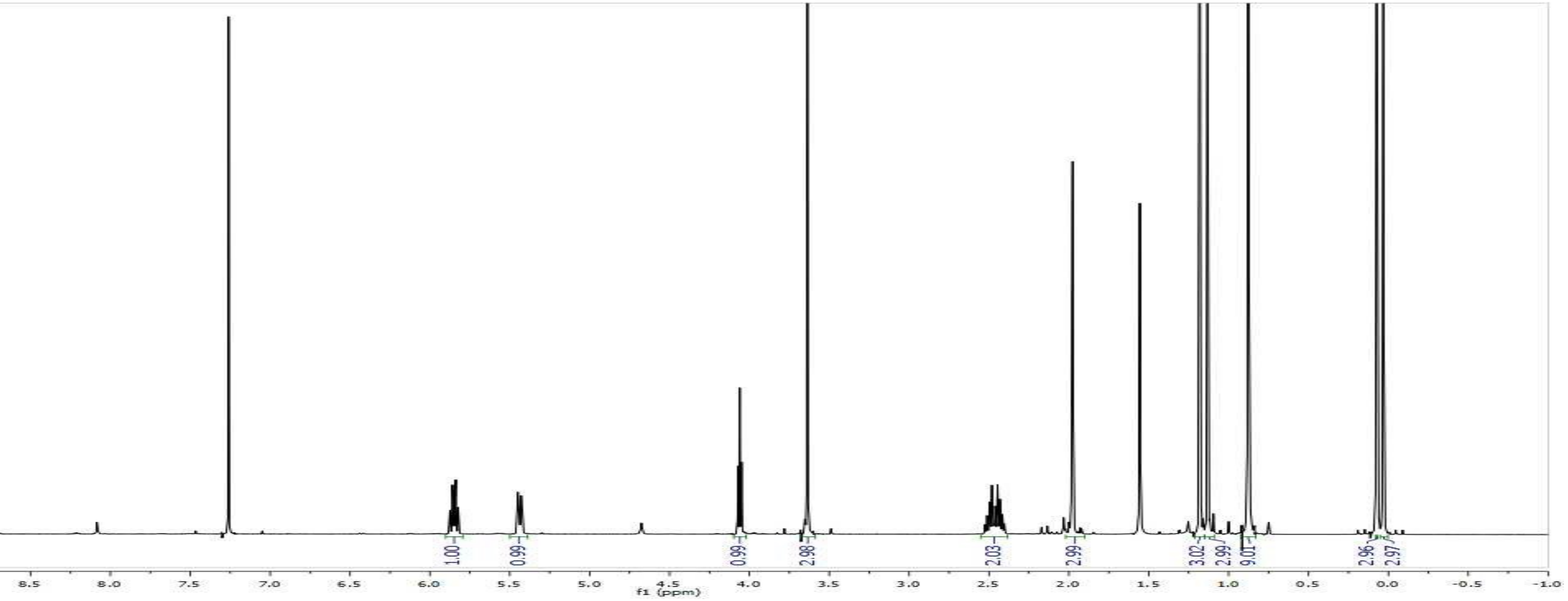
1.00
0.99
0.99
2.98
2.03
2.99
3.02
2.99
9.01
2.96
2.97
f1 (ppm)

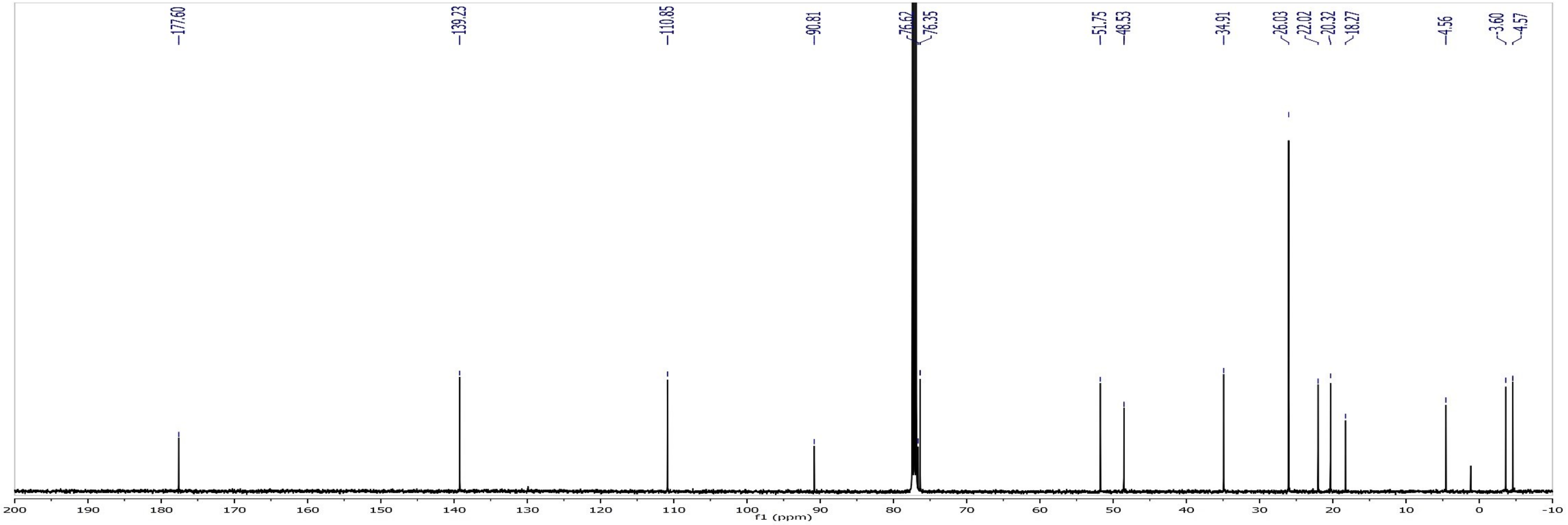
177.60
139.23
110.85
90.81
76.62
76.35
51.75
48.53
34.91
26.03
22.02
20.32
18.27
-4.56
-3.60
-4.57
f1 (ppm)

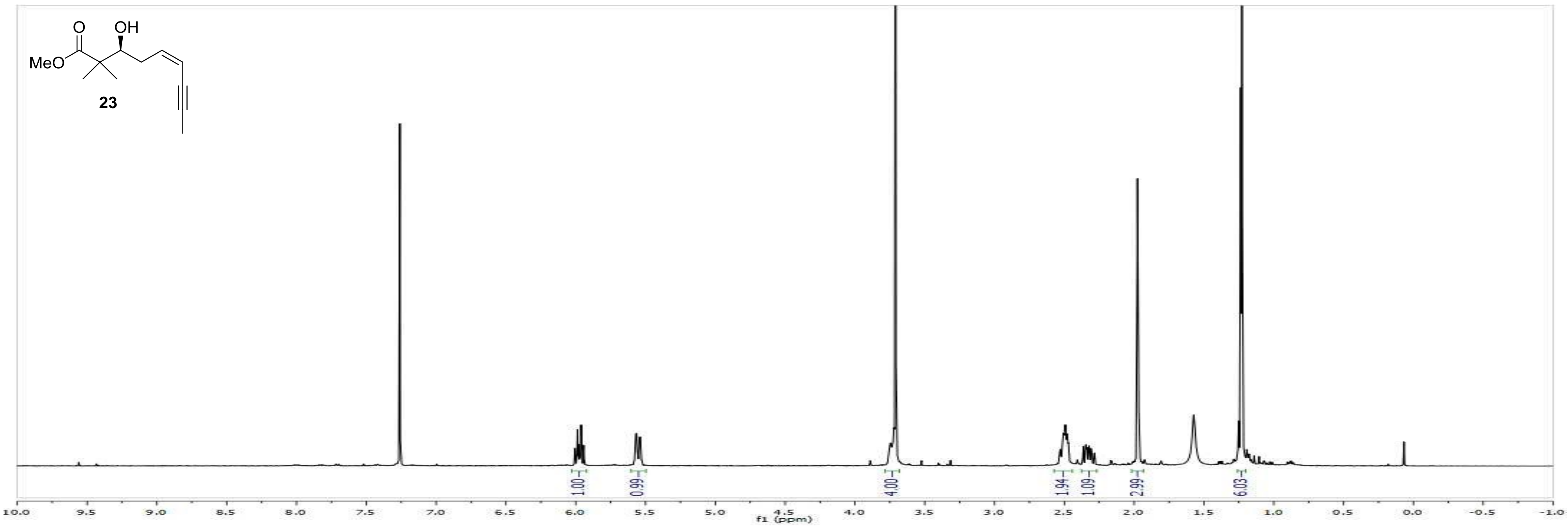

23
1.00
0.99
4.00
1.94
1.09
2.99
6.03
10.0 9.5 9.0 8.5 8.0 7.5 7.0 6.5 6.0 5.5 5.0 4.5 4.0 3.5 3.0 2.5 2.0 1.5 1.0 0.5 0.0 -0.5 -1.0
f1 (ppm)

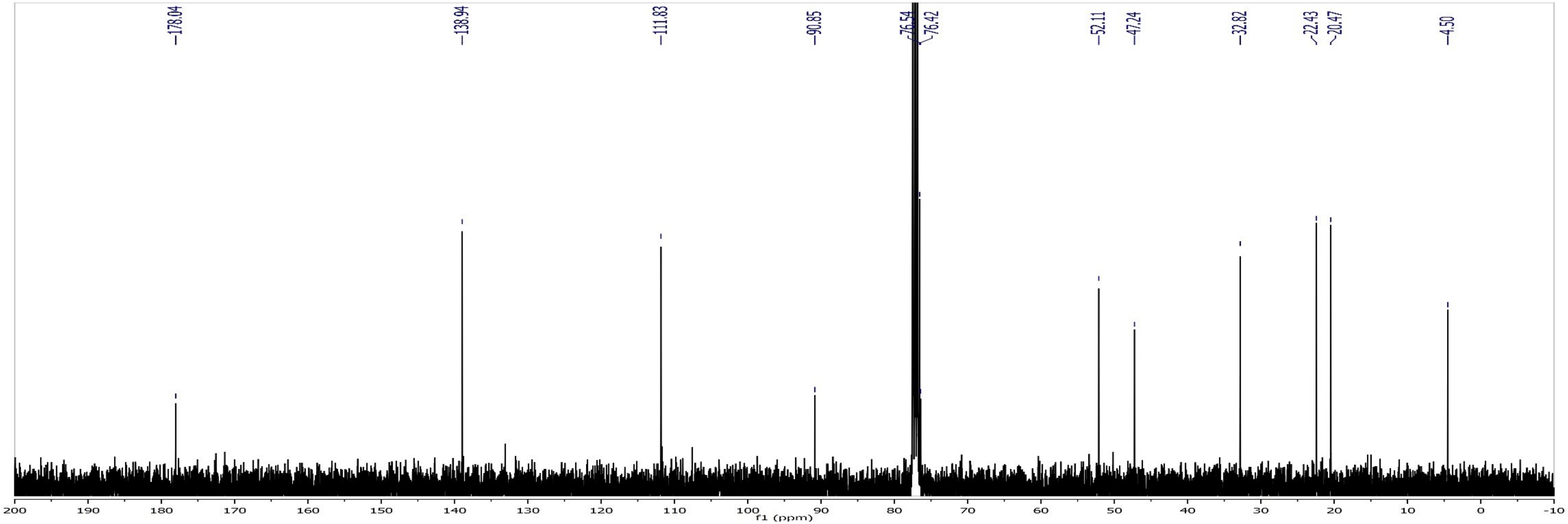

178.04
138.94
111.83
90.85
76.54
76.42
52.11
47.24
32.82
22.43
20.47
4.50
200 190 180 170 160 150 140 130 120 110 100 90 80 70 60 50 40 30 20 10 0 -10
f1 (ppm)

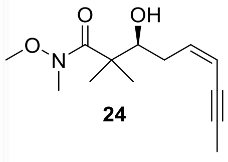

24

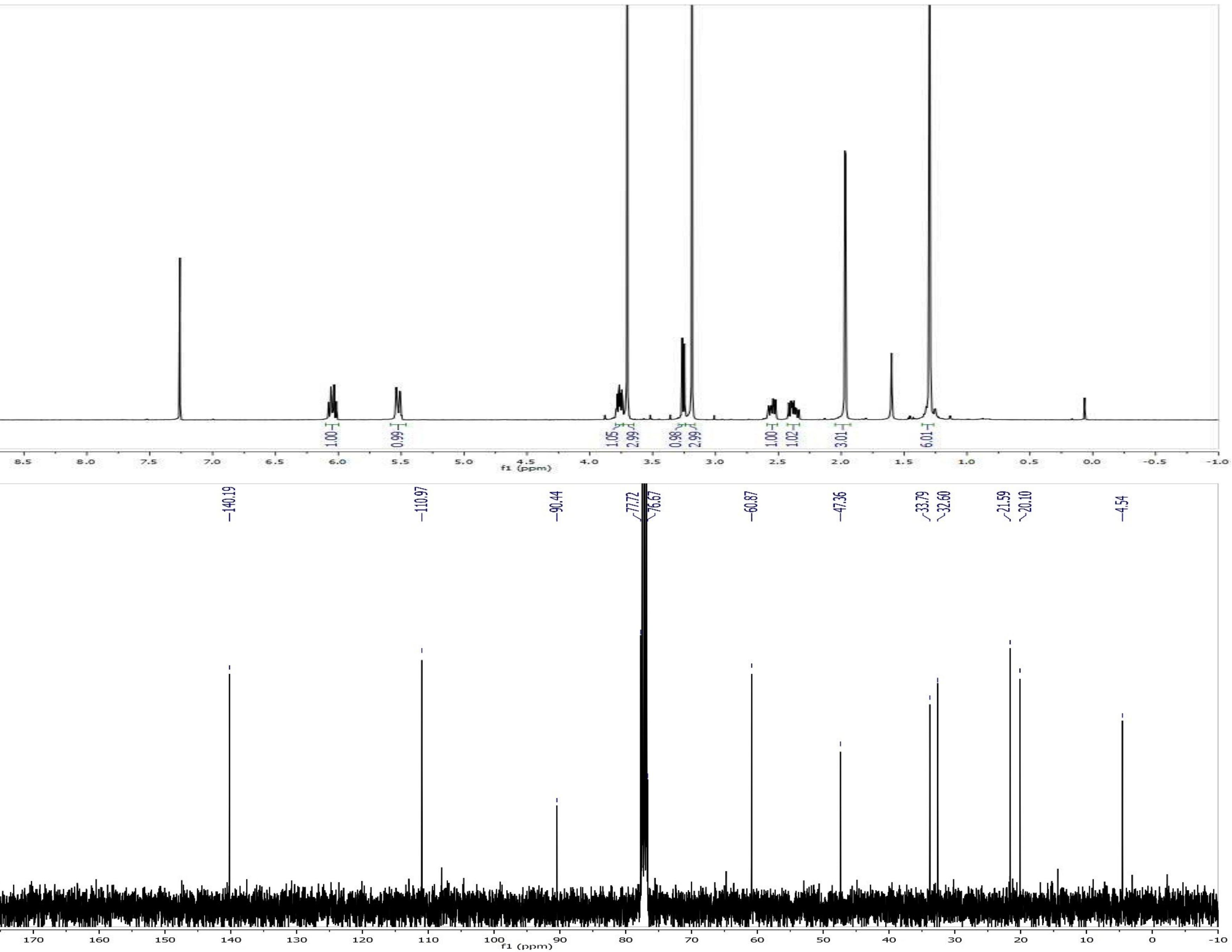
1.00
0.99
1.05
2.99
0.98
2.99
1.00
1.02
3.01
6.01
f1 (ppm)
178.64
140.19
110.97
90.44
77.72
76.67
60.87
47.36
33.79
32.60
21.59
20.10
4.54
f1 (ppm)

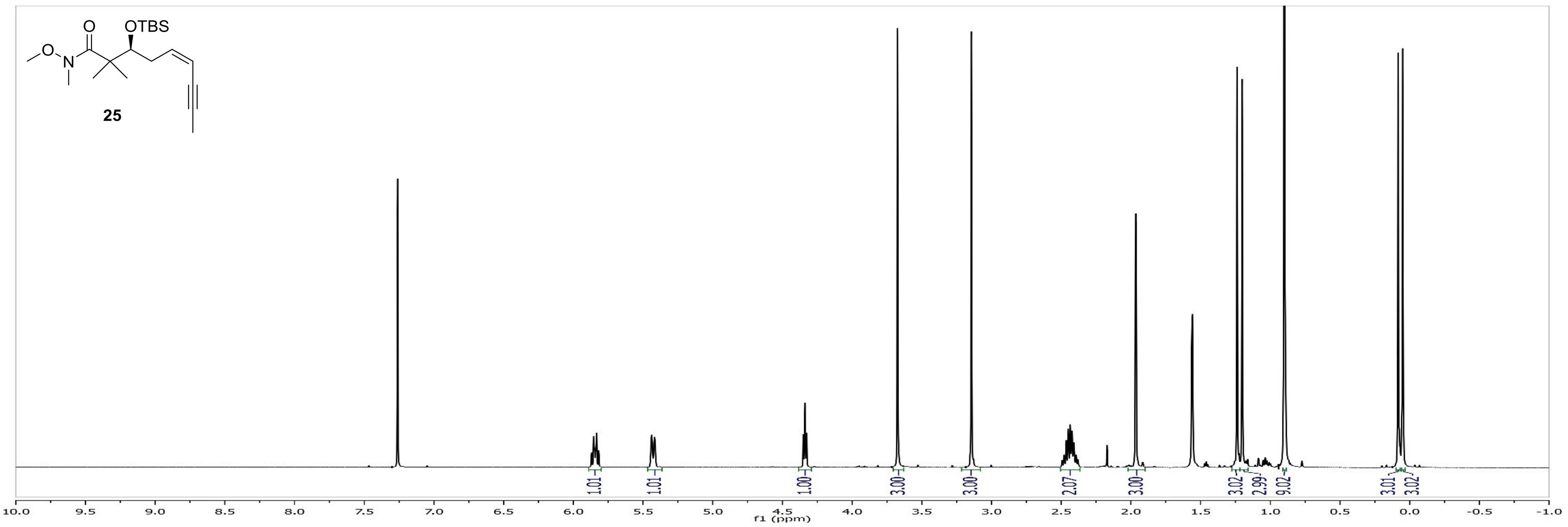

O
OTBS
N
O
25
1.01
1.01
1.00
3.00
3.00
2.07
3.00
3.02
2.99
9.02
3.01
3.02
f1 (ppm)

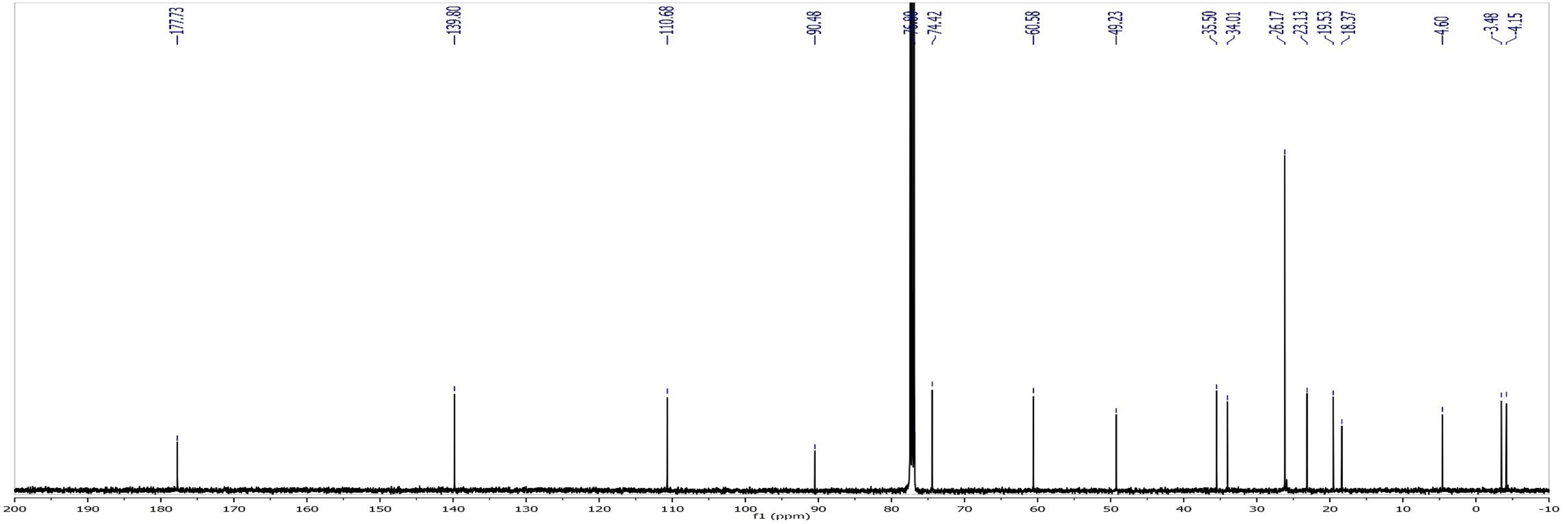

177.73
139.80
110.68
90.48
74.42
60.58
49.23
35.50
34.01
26.17
23.13
19.53
18.37
4.60
-3.48
-4.15
f1 (ppm)

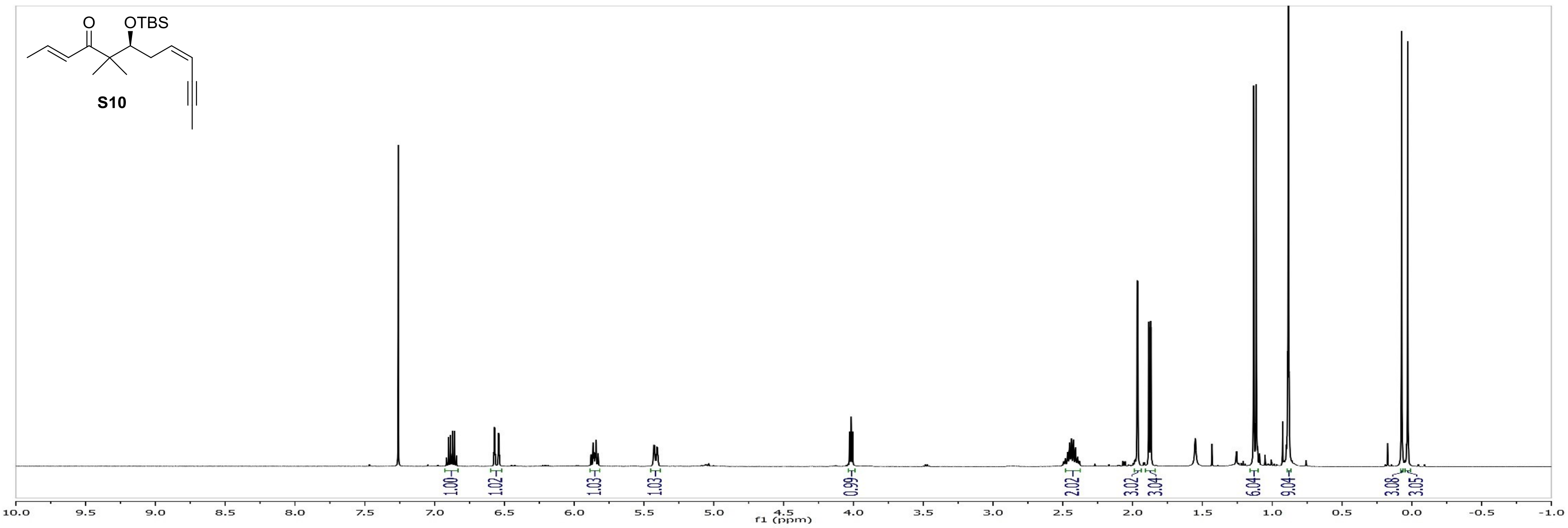
O
OTBS
S10
1.00
1.02
1.03
1.03
0.99
2.02
3.02
3.04
6.04
9.04
3.08
3.05
10.0 9.5 9.0 8.5 8.0 7.5 7.0 6.5 6.0 5.5 5.0 4.5 4.0 3.5 3.0 2.5 2.0 1.5 1.0 0.5 0.0 -0.5 -1.0
f1 (ppm)

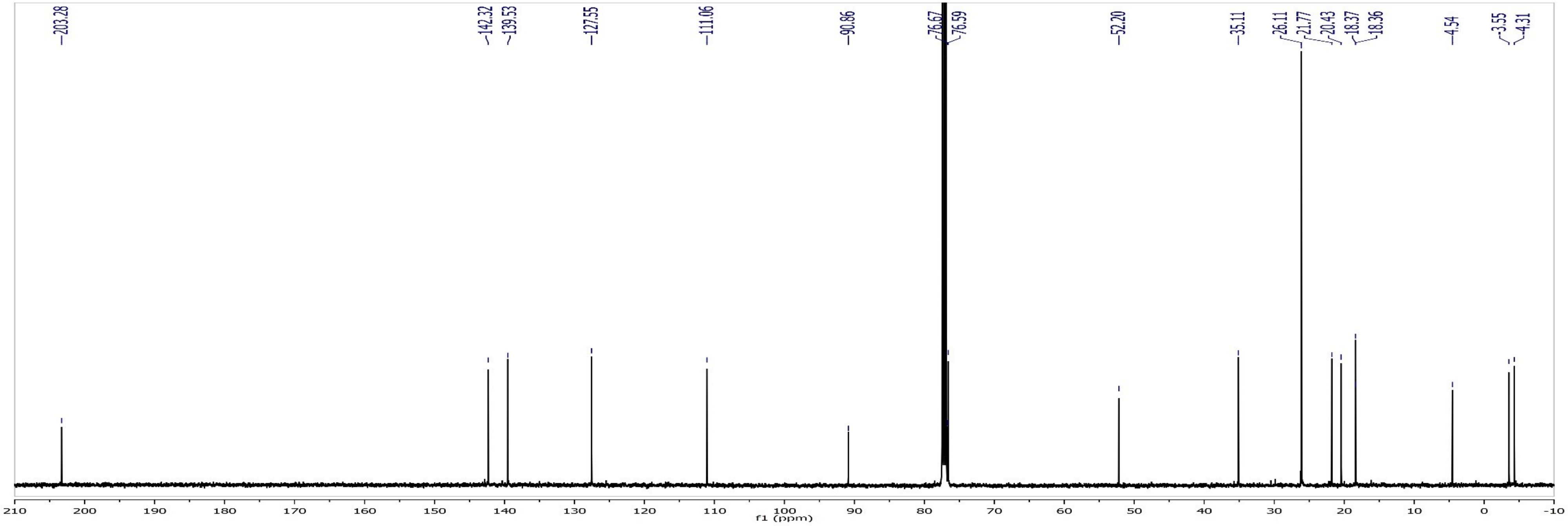
203.28
142.32
139.53
127.55
111.06
90.86
76.67
76.59
52.20
35.11
26.11
21.77
20.43
18.37
18.36
4.54
-3.55
-4.31
210 200 190 180 170 160 150 140 130 120 110 100 90 80 70 60 50 40 30 20 10 0 -10
f1 (ppm)

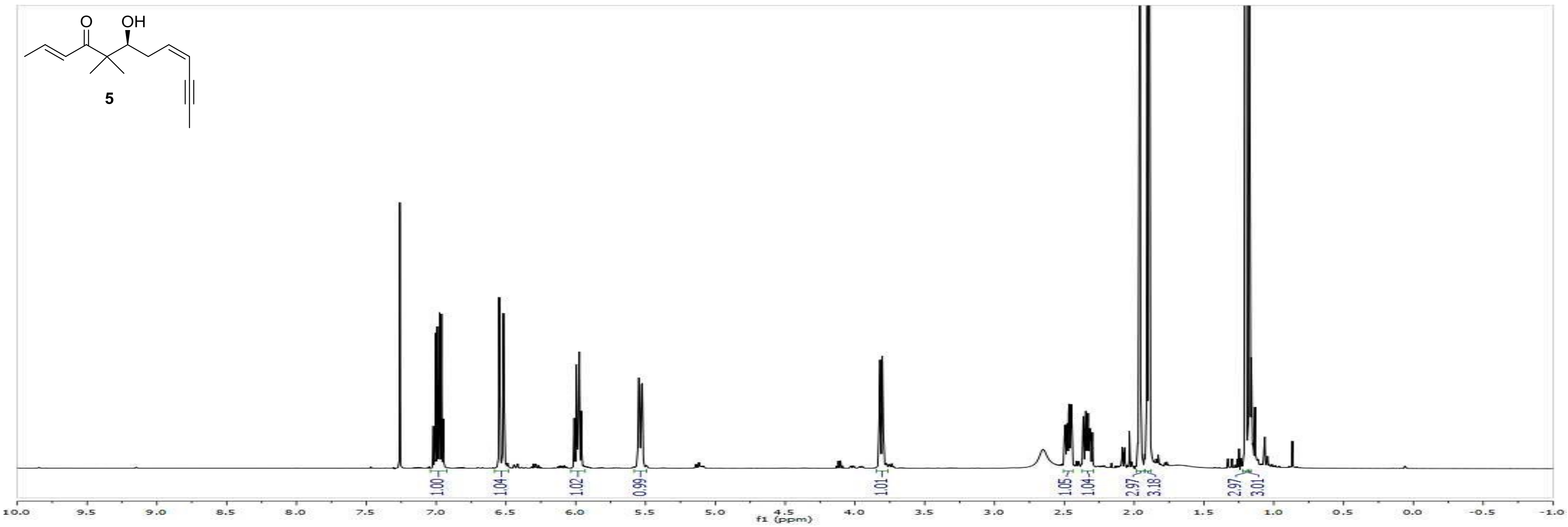
OH
O
5
1.00
1.04
1.02
0.99
1.01
1.05
1.04
2.97
3.18
2.97
3.01
10.0 9.5 9.0 8.5 8.0 7.5 7.0 6.5 6.0 5.5 5.0 4.5 4.0 3.5 3.0 2.5 2.0 1.5 1.0 0.5 0.0 -0.5 -1.0
f1 (ppm)

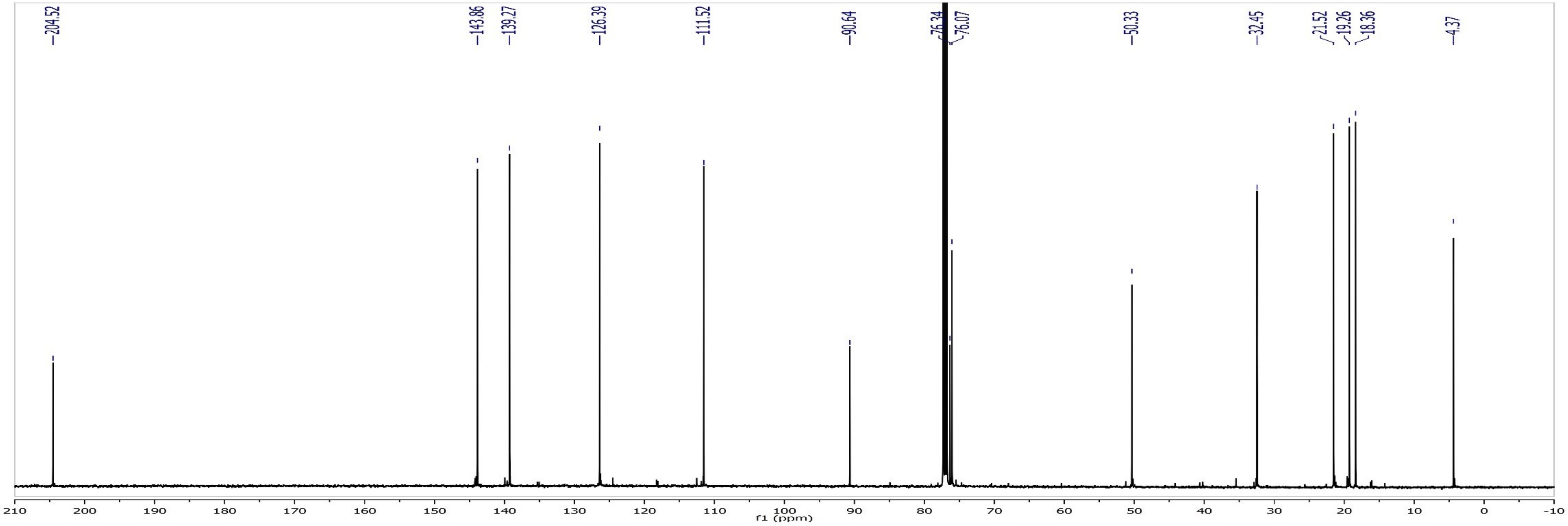
204.52
143.86
139.27
126.39
111.52
90.64
76.34
76.07
50.33
32.45
21.52
19.26
18.36
4.37
210 200 190 180 170 160 150 140 130 120 110 100 90 80 70 60 50 40 30 20 10 0 -10
f1 (ppm)

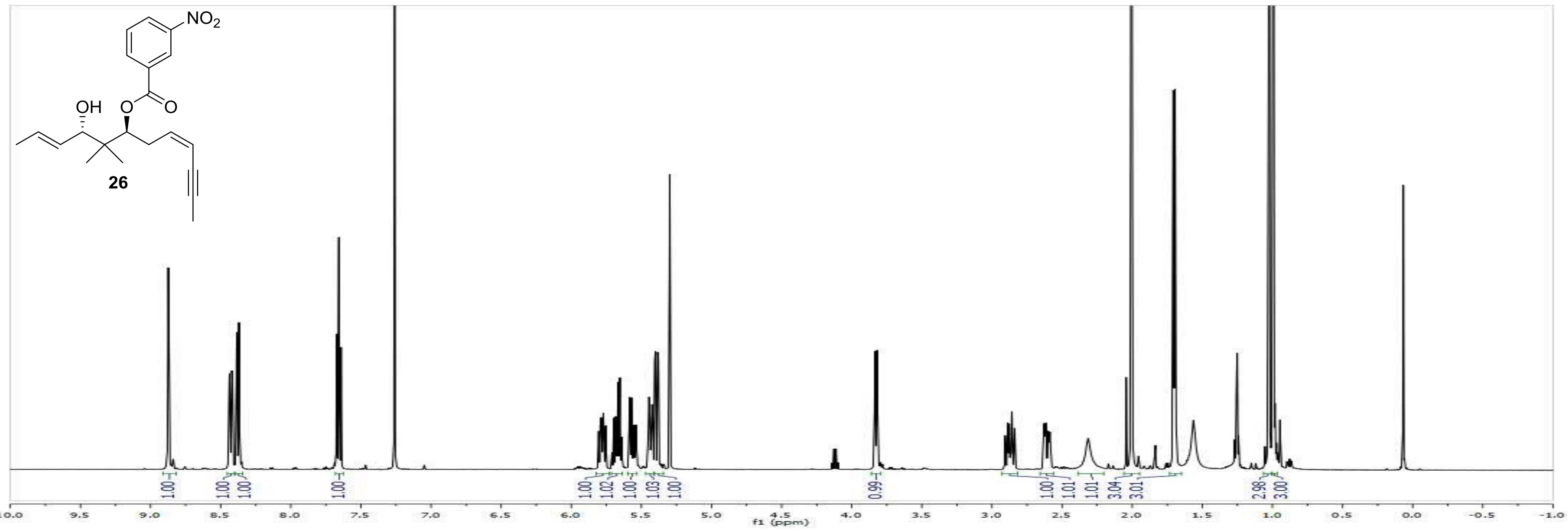
NO2
OH
26
1.00
1.00
1.00
1.00
1.00
1.02
1.00
1.03
1.00
0.99
1.00
1.01
1.01
3.04
3.01
2.98
3.00
f1 (ppm)

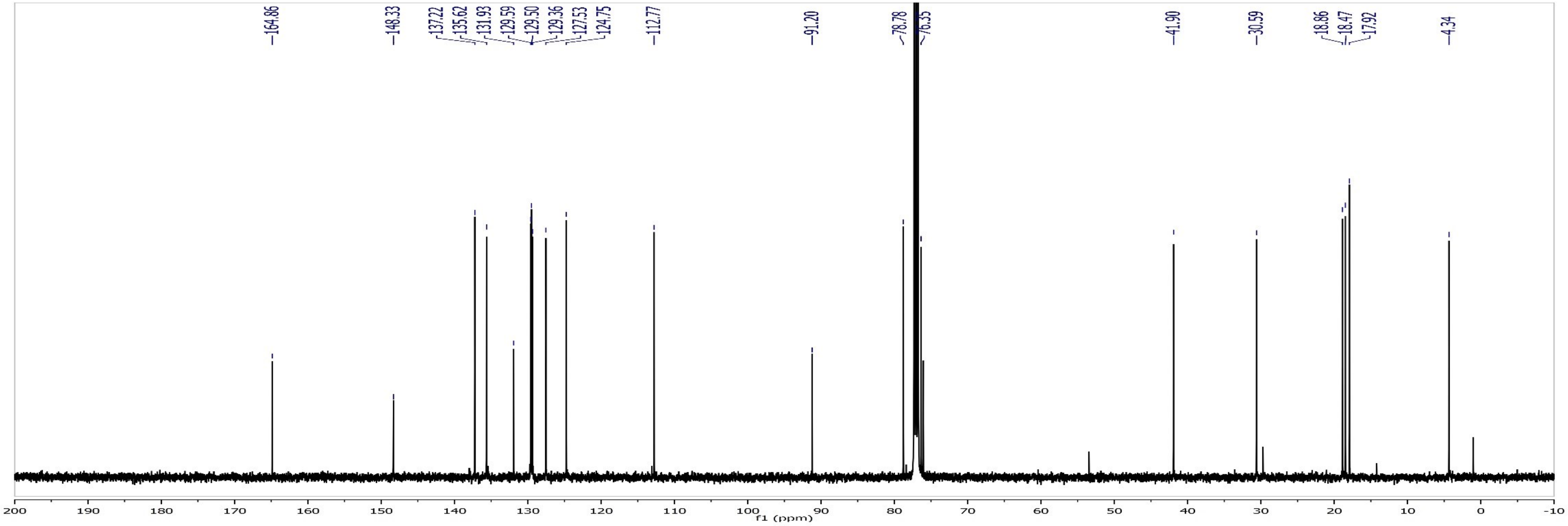
164.86
148.33
137.22
135.62
131.93
129.59
129.50
129.36
127.53
124.75
112.77
91.20
78.78
76.35
41.90
30.59
18.86
18.47
17.92
4.34
f1 (ppm)

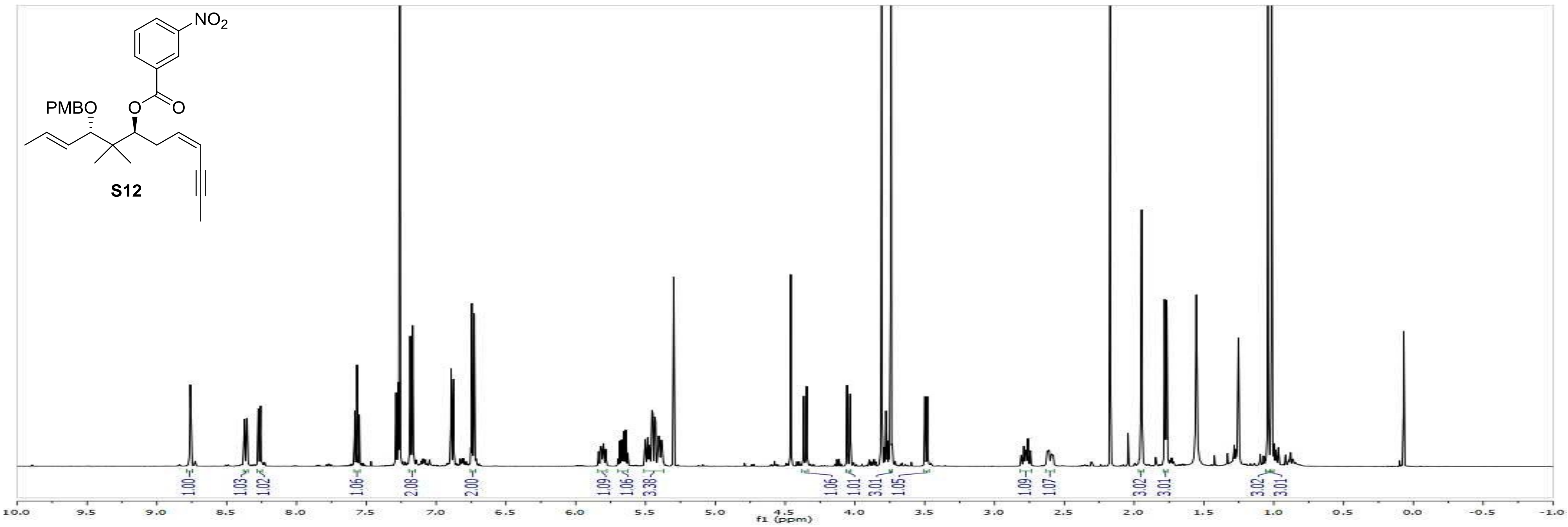
NO2
PMBO
O
O
O
S12
1.00
1.03
1.02
1.06
2.08
2.00
1.09
1.06
3.38
1.06
1.01
3.01
1.05
1.09
1.07
3.02
3.01
3.02
3.01
f1 (ppm)

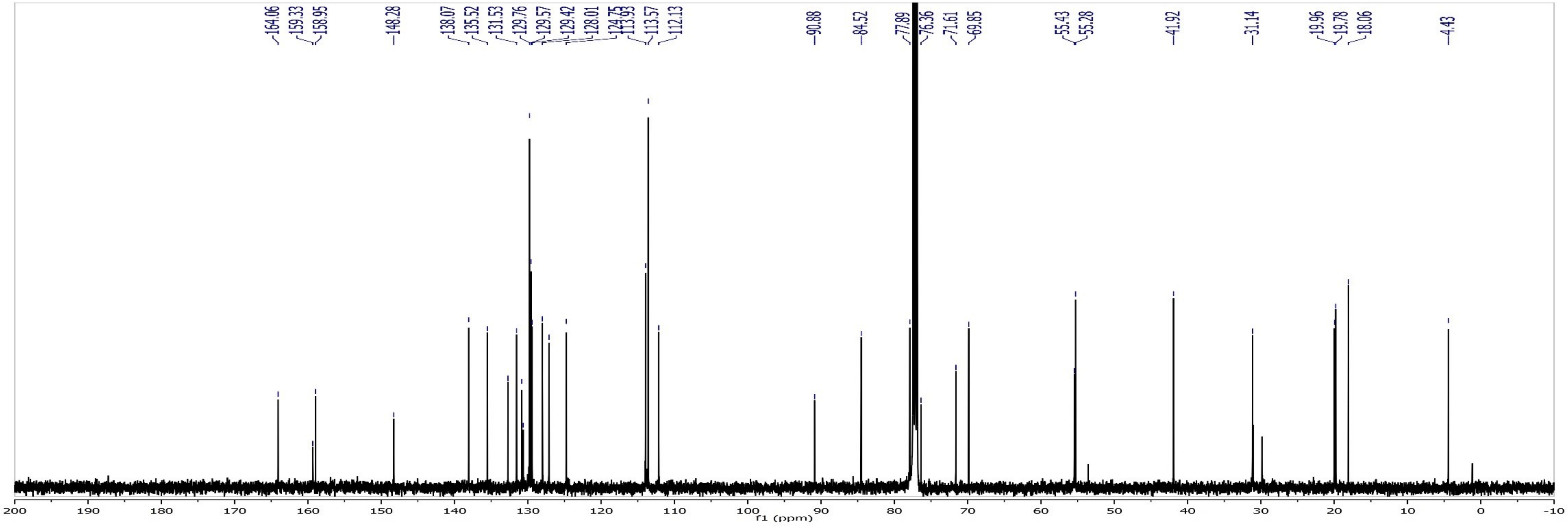
164.06
159.33
158.95
148.28
138.07
135.52
131.53
129.76
129.57
129.42
128.01
124.75
113.93
113.57
112.13
90.88
84.52
77.89
76.36
71.61
69.85
55.43
55.28
41.92
31.14
19.96
19.78
18.06
4.43
f1 (ppm)

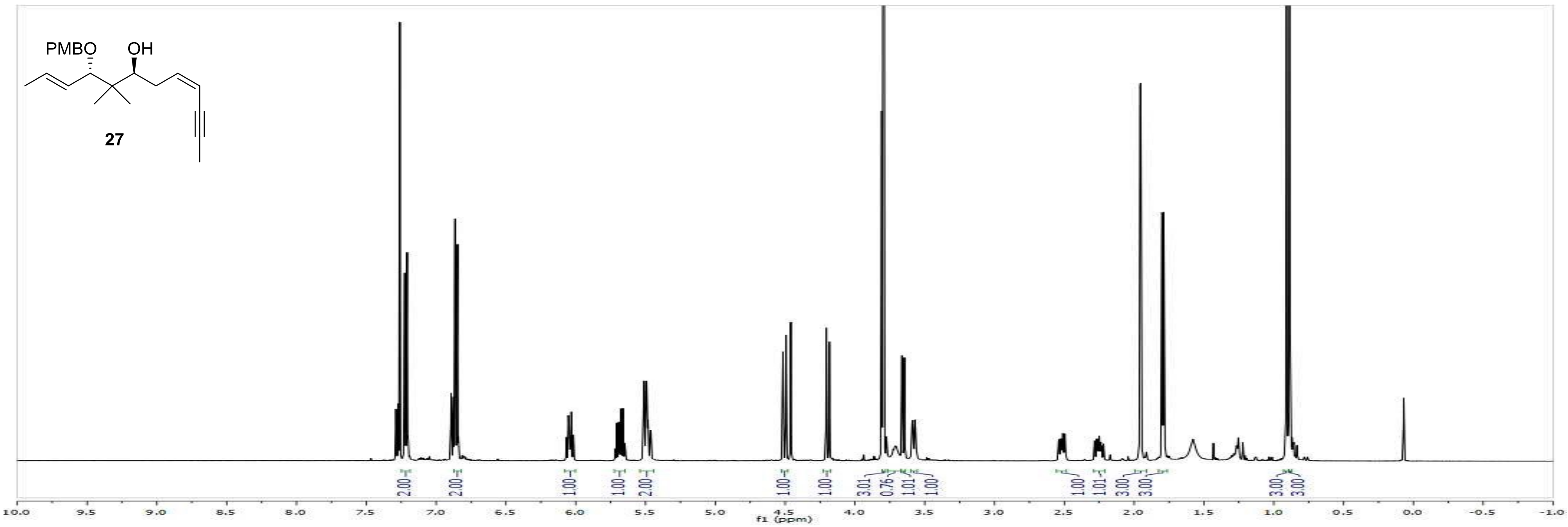
PMBO
OH
27
2.00
2.00
1.00
1.00
2.00
1.00
1.00
3.01
0.76
1.01
1.00
1.00
1.01
3.00
3.00
3.00
3.00
f1 (ppm)

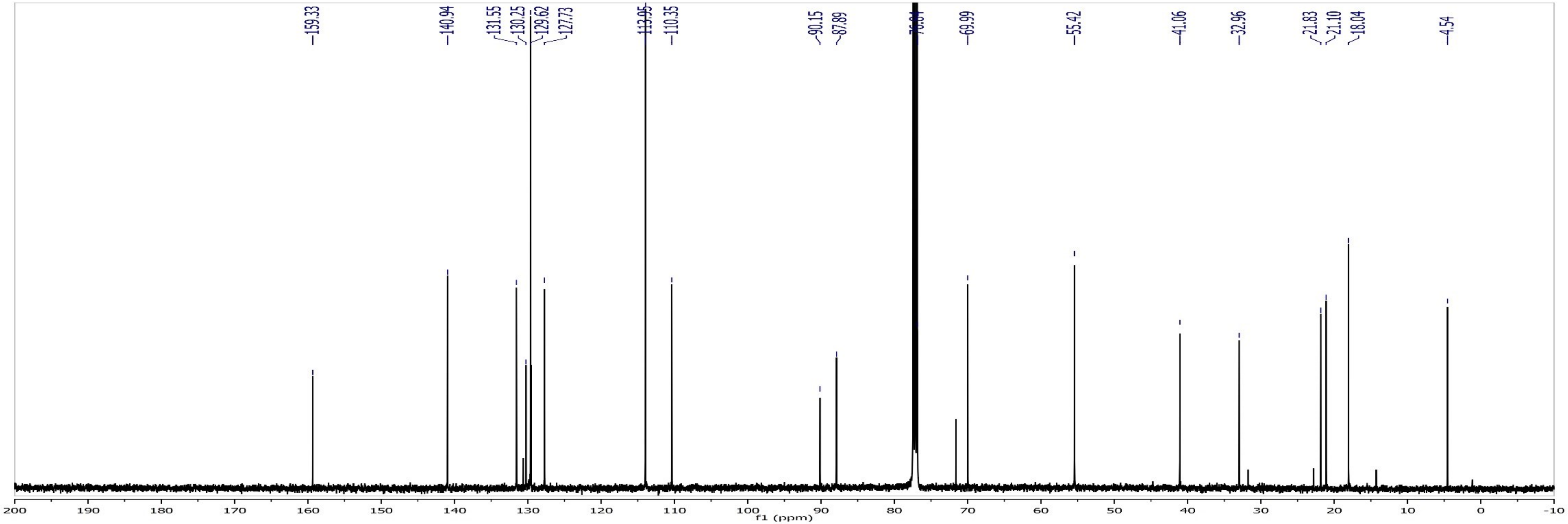
159.33
140.94
131.55
130.25
129.62
127.73
113.95
110.35
90.15
87.89
69.99
55.42
41.06
32.96
21.83
21.10
18.04
4.54
f1 (ppm)

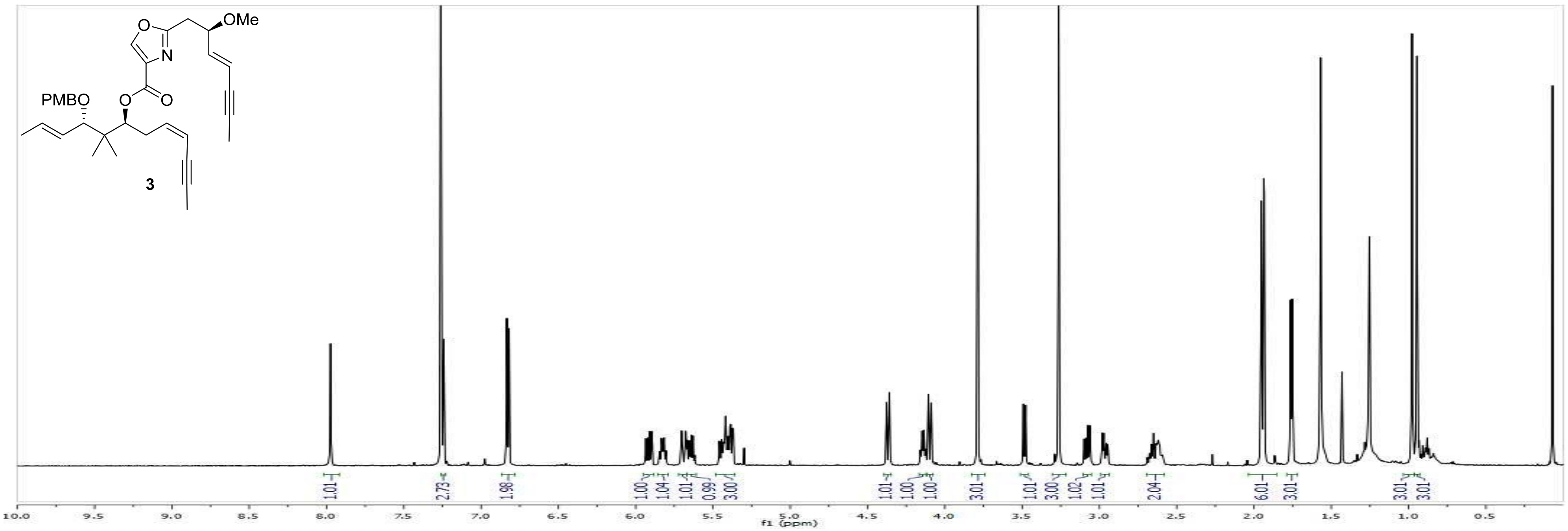
OMe
O
N
PMBO
O
O
3
1.01
2.73
1.98
1.00
1.04
1.01
0.99
3.00
1.01
1.00
1.00
3.01
1.01
3.00
1.02
1.01
2.04
6.01
3.01
3.01
3.01
10.0 9.5 9.0 8.5 8.0 7.5 7.0 6.5 6.0 5.5 5.0 4.5 4.0 3.5 3.0 2.5 2.0 1.5 1.0 0.5
f1 (ppm)

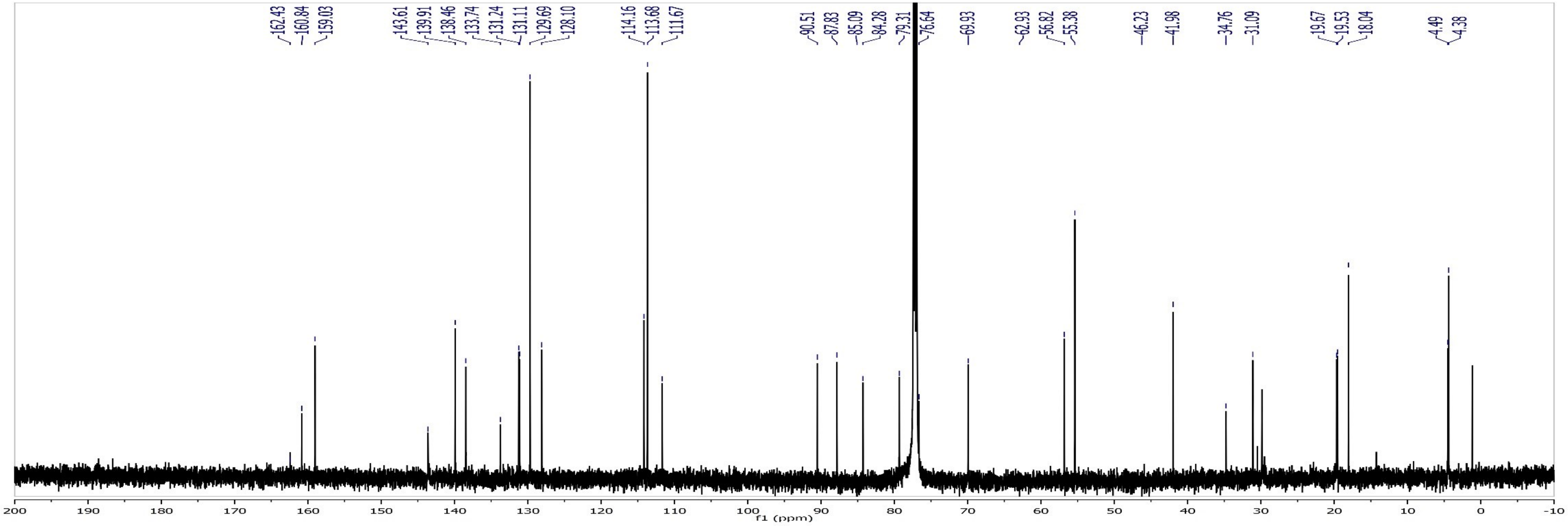
162.43
160.84
159.03
143.61
139.91
138.46
133.74
131.24
131.11
129.69
128.10
114.16
113.68
111.67
90.51
87.83
85.09
84.28
79.31
76.64
69.93
62.93
56.82
55.38
46.23
41.98
34.76
31.09
19.67
19.53
18.04
4.49
4.38
200 190 180 170 160 150 140 130 120 110 100 90 80 70 60 50 40 30 20 10 0 -10
f1 (ppm)

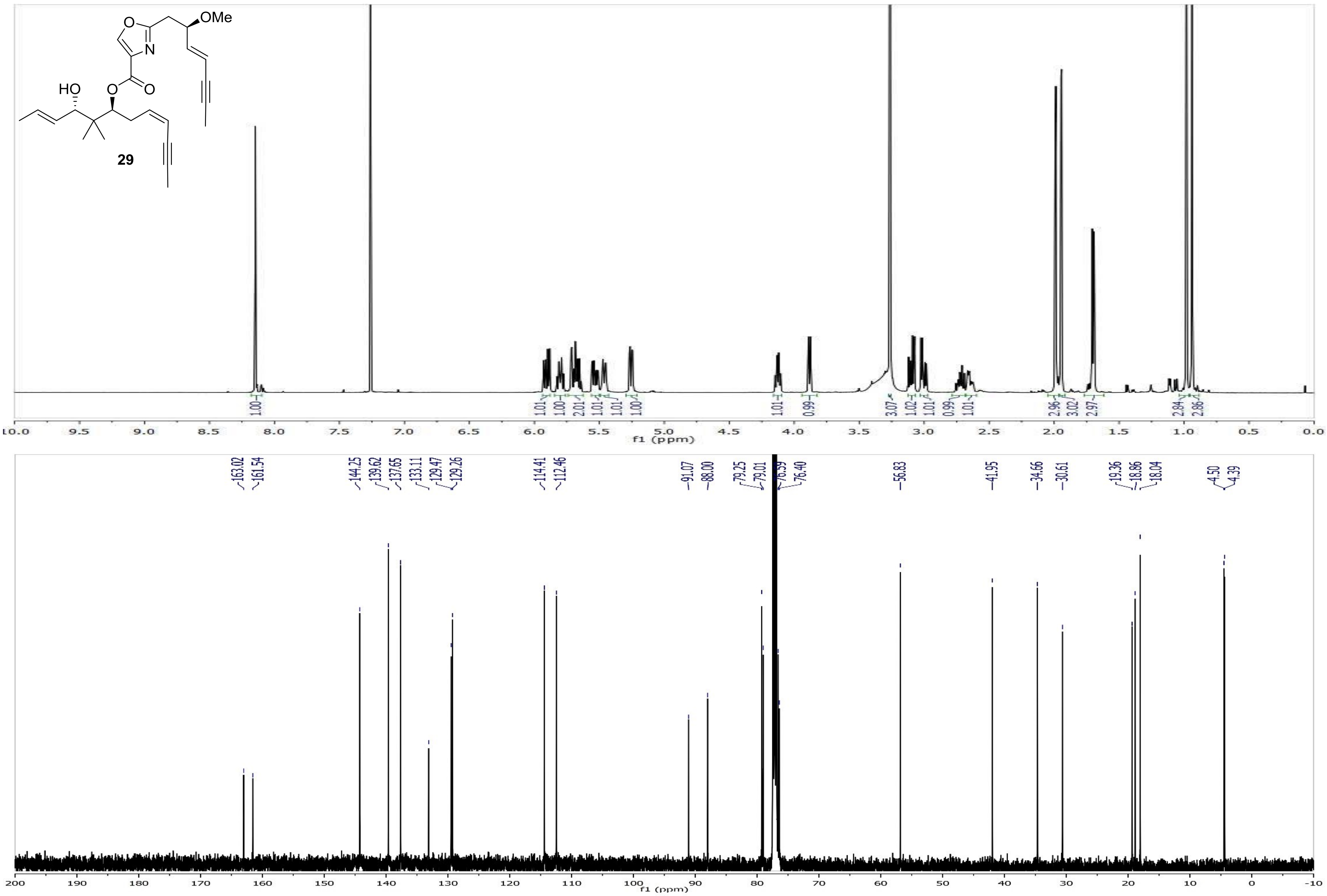
OMe
HO
29
f1 (ppm)

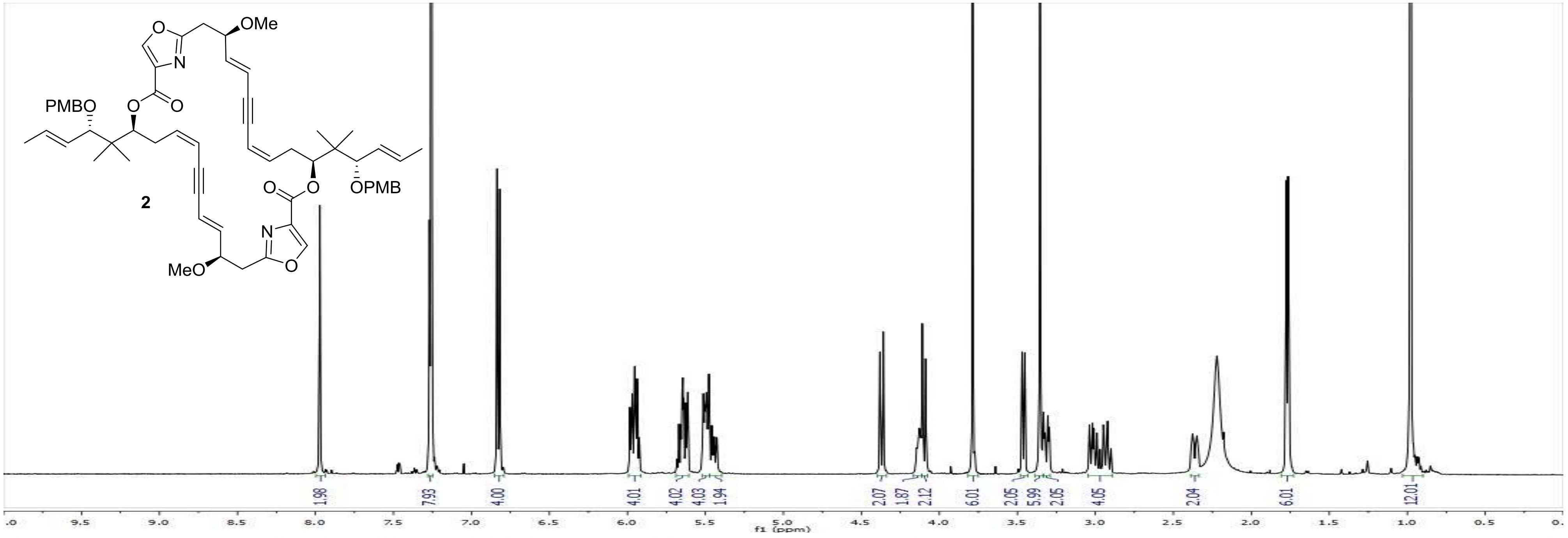
OMe
PMBO
OPMB
MeO
2
1.98
7.93
4.00
4.01
4.02
4.03
1.94
2.07
1.87
2.12
6.01
2.05
5.99
2.05
4.05
2.04
6.01
12.01
f1 (ppm)

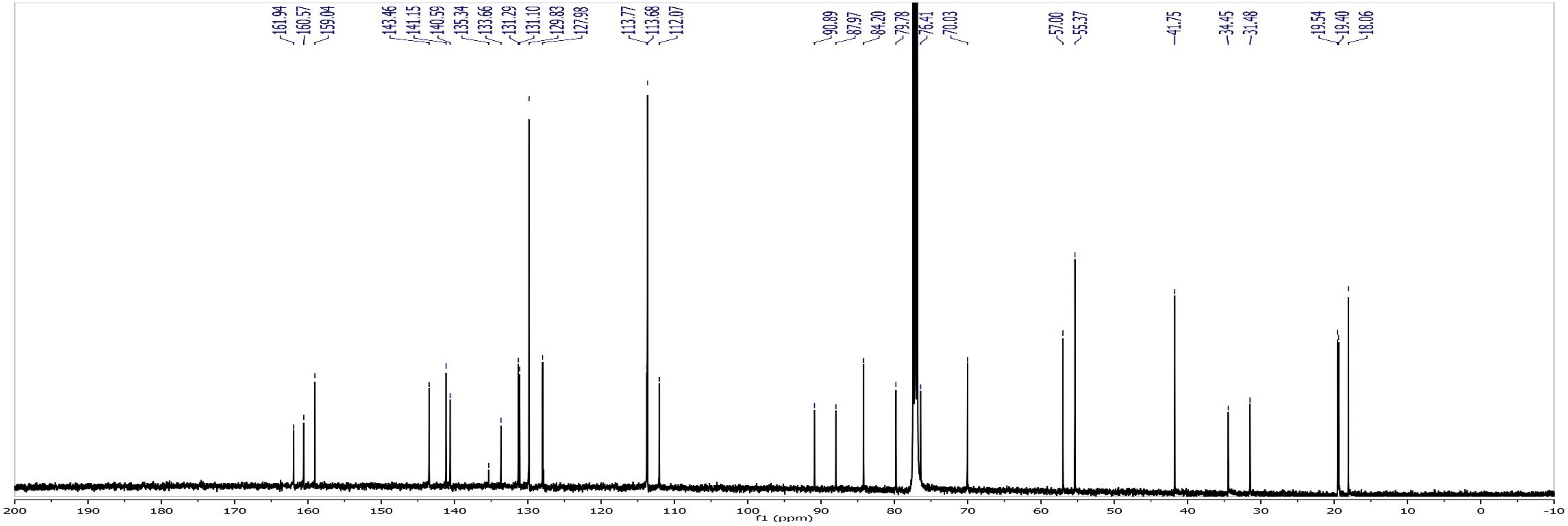
161.94
160.57
159.04
143.46
141.15
140.59
135.34
133.66
131.29
131.10
129.83
127.98
113.77
113.68
112.07
90.89
87.97
84.20
79.78
76.41
70.03
57.00
55.37
41.75
34.45
31.48
19.54
19.40
18.06
f1 (ppm)

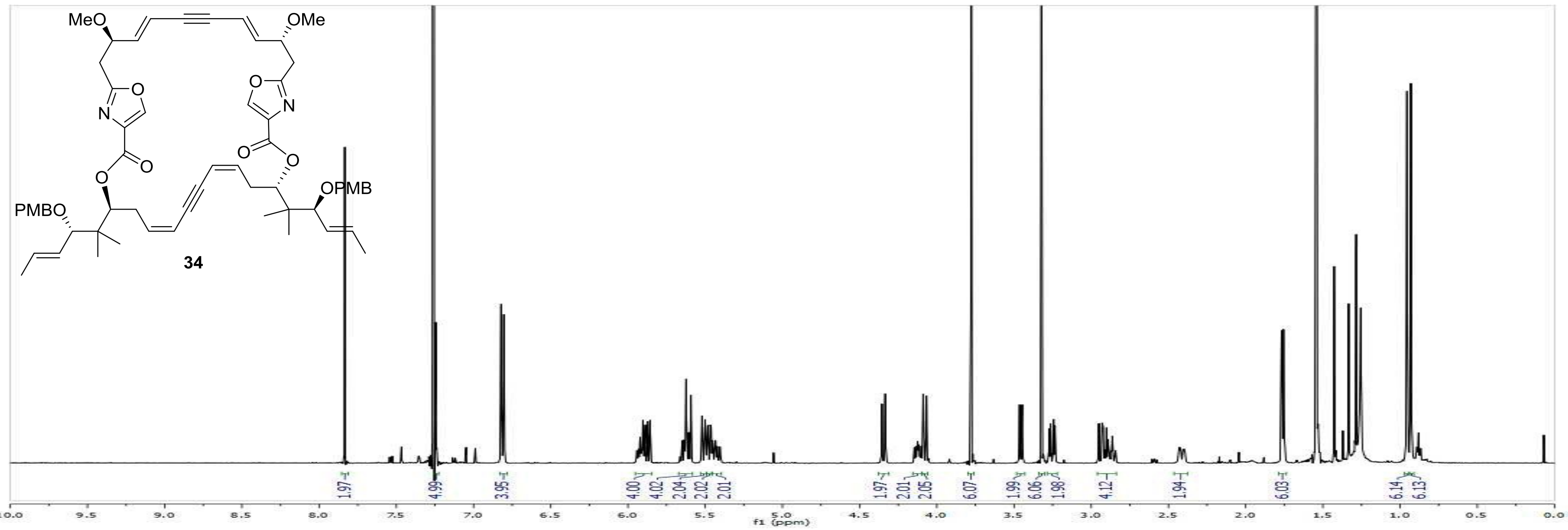
MeO
OMe
PMBO
OPMB
34
1.97
4.99
3.95
4.00
4.02
2.04
2.02
2.01
1.97
2.01
2.05
6.07
1.99
6.06
1.98
4.12
1.94
6.03
6.14
6.13
f1 (ppm)

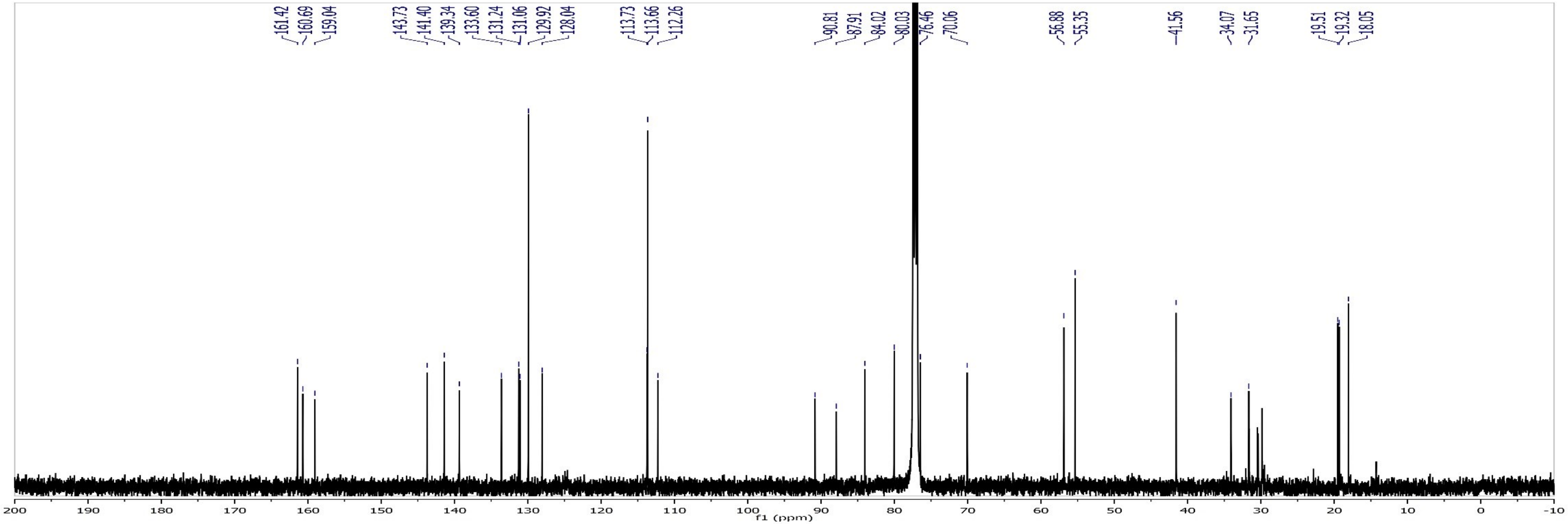
161.42
160.69
159.04
143.73
141.40
139.34
133.60
131.24
131.06
129.92
128.04
113.73
113.66
112.26
90.81
87.91
84.02
80.03
76.46
70.06
56.88
55.35
41.56
34.07
31.65
19.51
19.32
18.05
f1 (ppm)

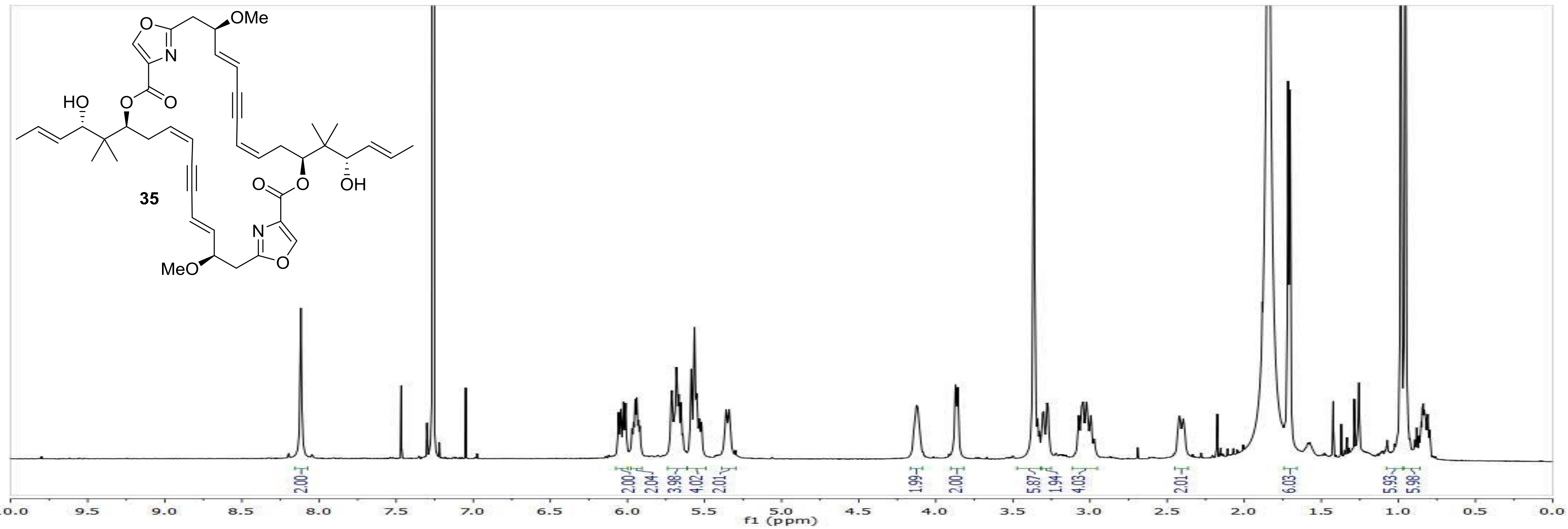
OMe
HO
OH
MeO
35
2.00
2.00
2.04
3.98
4.02
2.01
1.99
2.00
5.87
1.94
4.03
2.01
6.03
5.93
5.98
f1 (ppm)

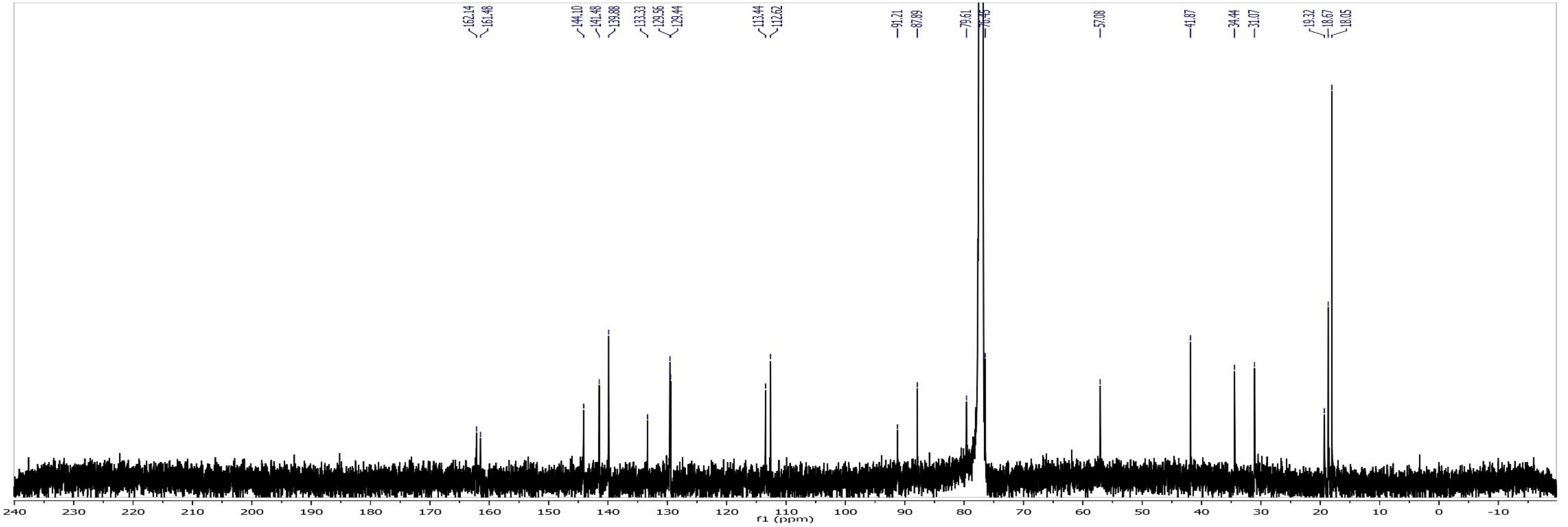
162.14
161.48
144.10
141.48
139.88
133.33
129.56
129.44
113.44
112.62
91.21
87.89
79.61
57.08
41.87
34.44
31.07
19.32
18.67
18.05
f1 (ppm)

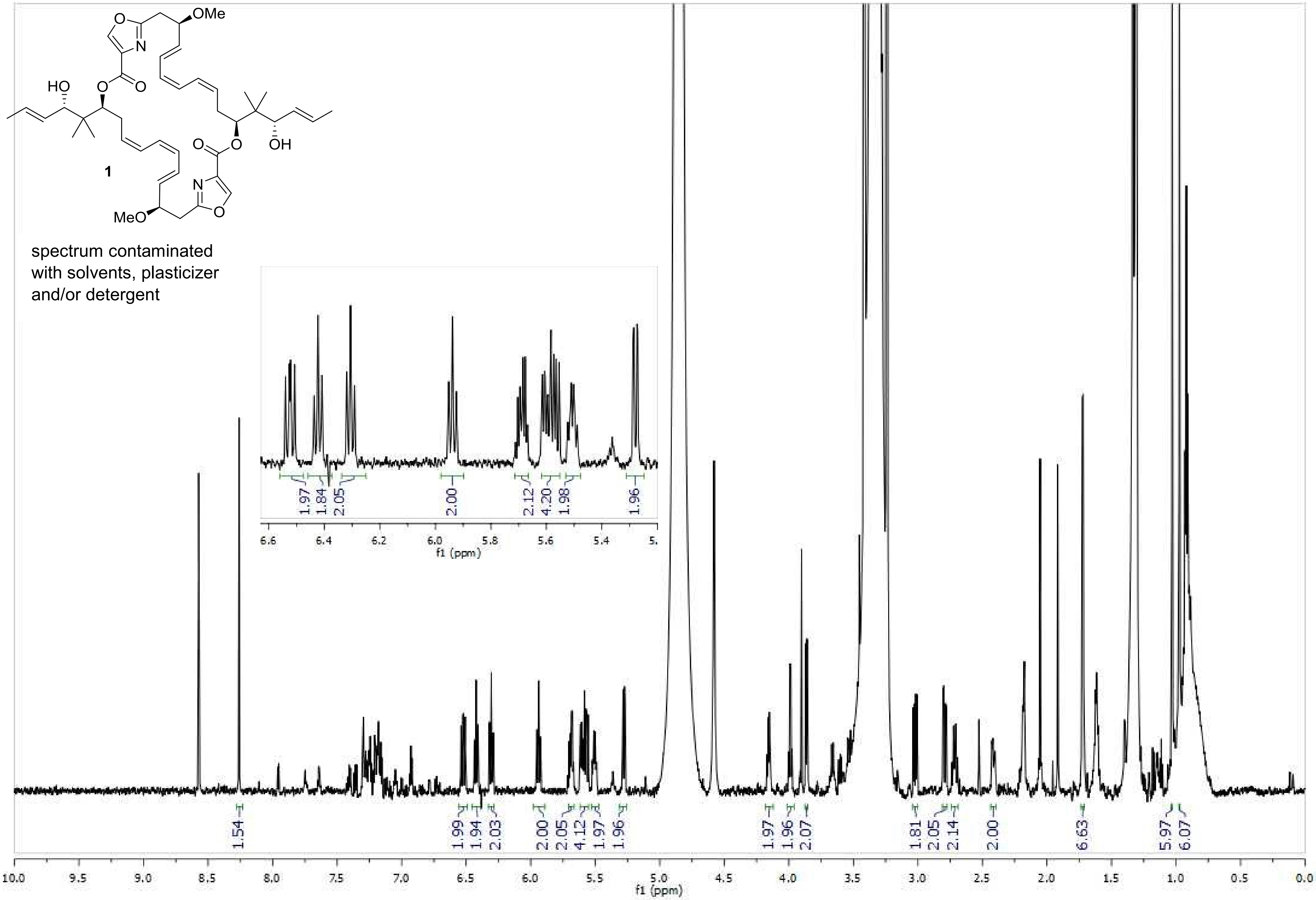
OMe
HO
O
N
OH
MeO
1
spectrum contaminated
with solvents, plasticizer
and/or detergent
1.97
1.84
2.05
2.00
2.12
4.20
1.98
1.96
6.6
6.4
6.2
6.0
5.8
5.6
5.4
f1 (ppm)
1.54
1.99
1.94
2.03
2.00
2.05
4.12
1.97
1.96
1.97
1.96
2.07
1.81
2.05
2.14
2.00
6.63
5.97
6.07
10.0
9.5
9.0
8.5
8.0
7.5
7.0
6.5
6.0
5.5
5.0
4.5
4.0
3.5
3.0
2.5
2.0
1.5
1.0
0.5
0.0
f1 (ppm)

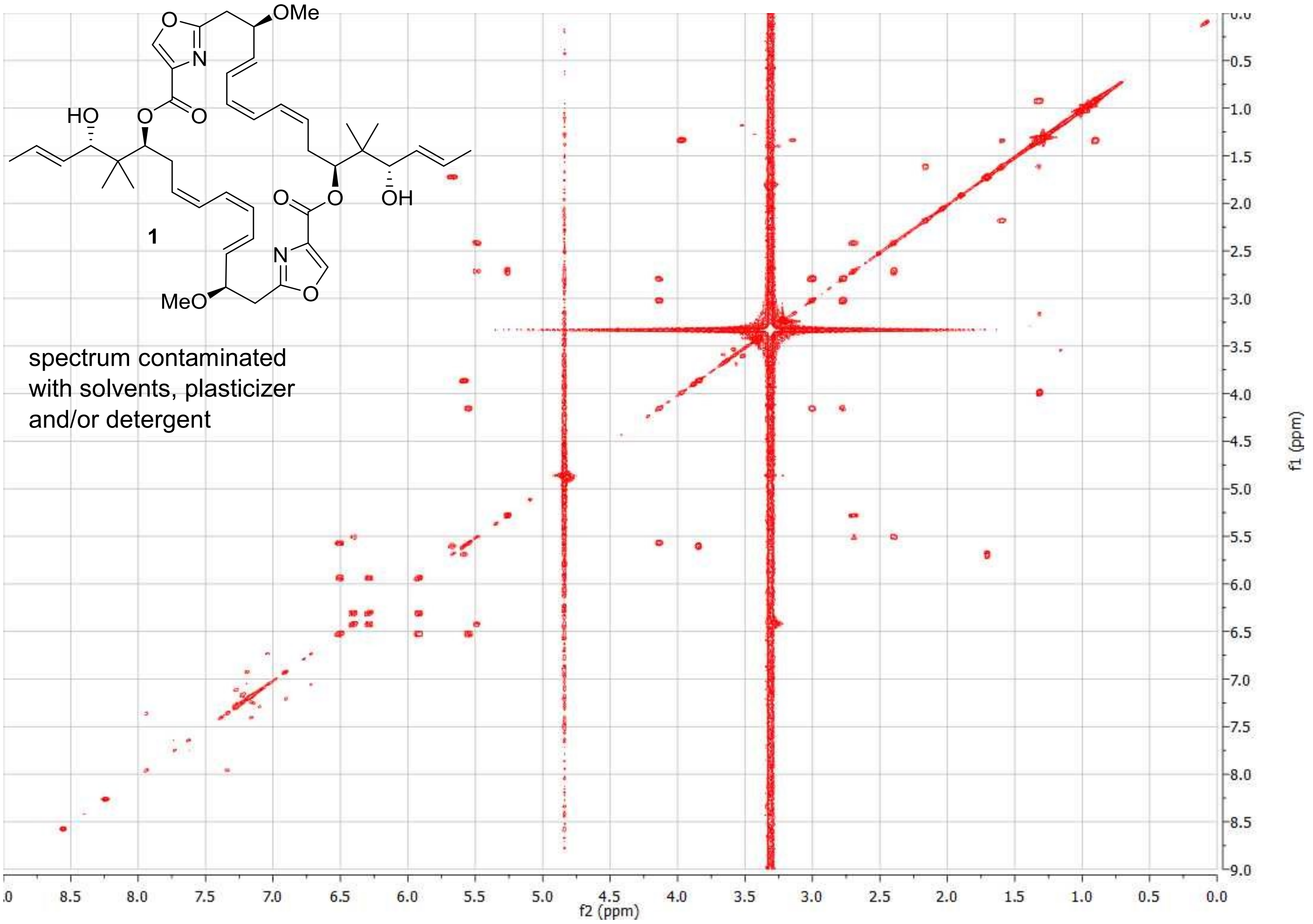
OMe
HO
OH
MeO
1
spectrum contaminated with solvents, plasticizer and/or detergent
f2 (ppm)
f1 (ppm)

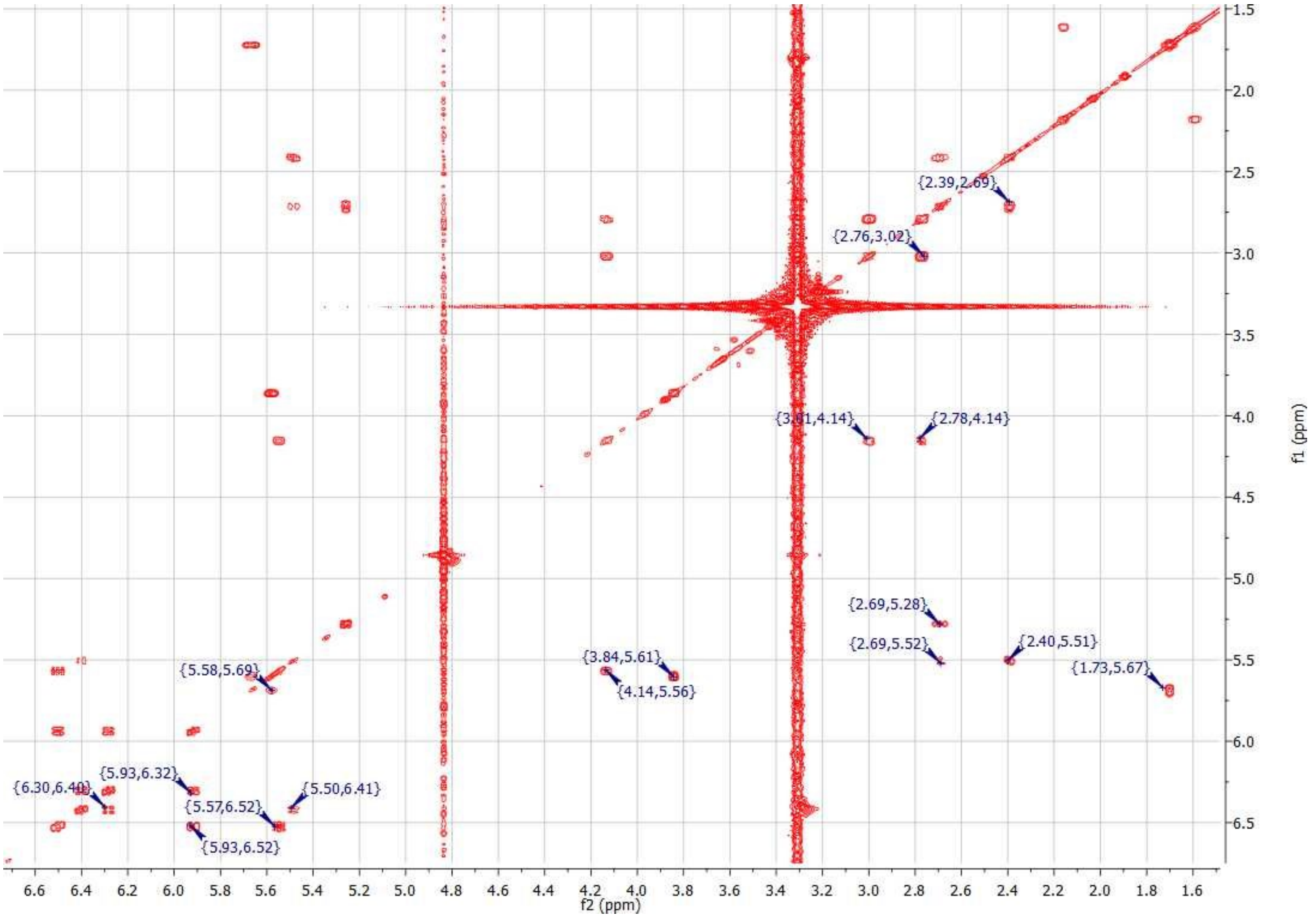
{2.39,2.69}
{2.76,3.02}
{3.01,4.14}
{2.78,4.14}
{2.69,5.28}
{2.69,5.52}
{2.40,5.51}
{1.73,5.67}
{3.84,5.61}
{4.14,5.56}
{5.58,5.69}
{5.93,6.32}
{6.30,6.40}
{5.50,6.41}
{5.57,6.52}
{5.93,6.52}
f2 (ppm)
f1 (ppm)
